# Supplementary figures and images for: An open-source, high-performance tool for automated sleep staging
Source: eLife. 2021 Oct 14;10:e70092. doi: 10.7554/eLife.70092 (PMC8516415; doi:10.7554/eLife.70092)

68f038f2

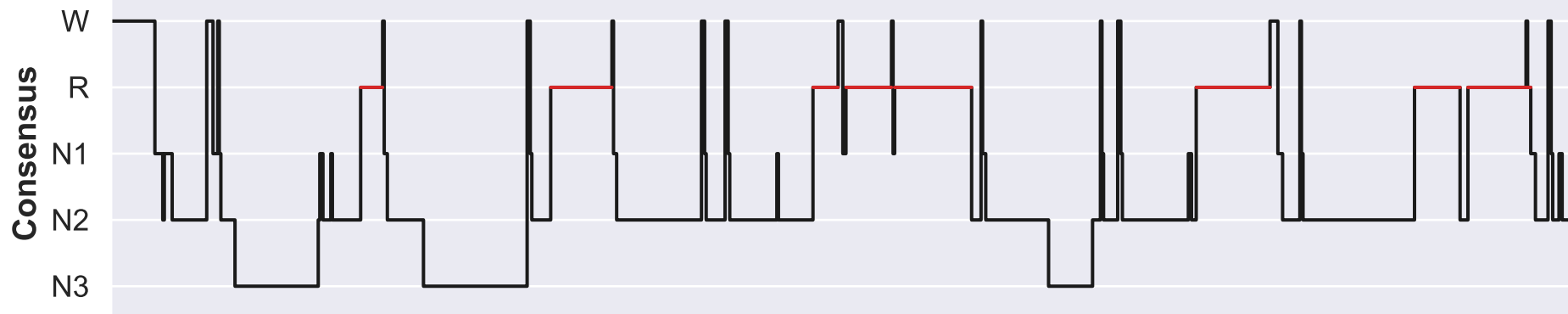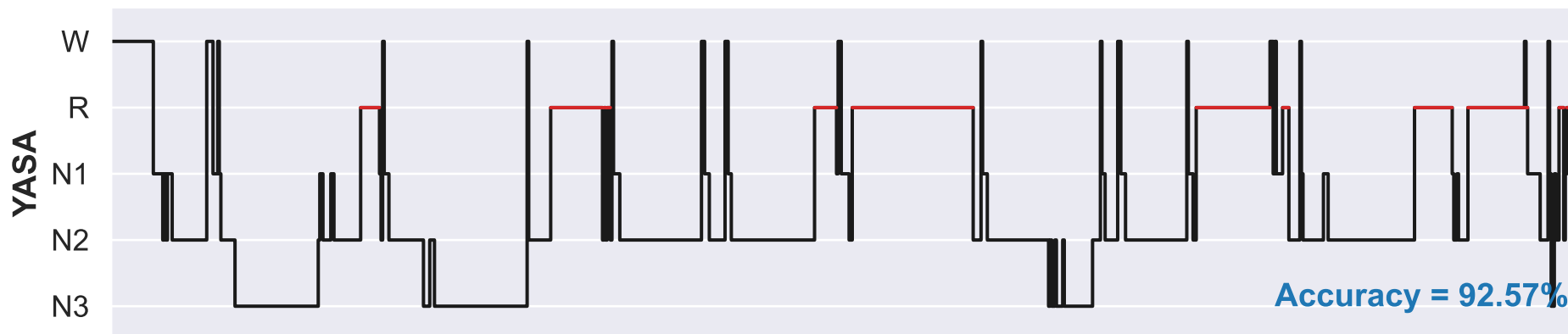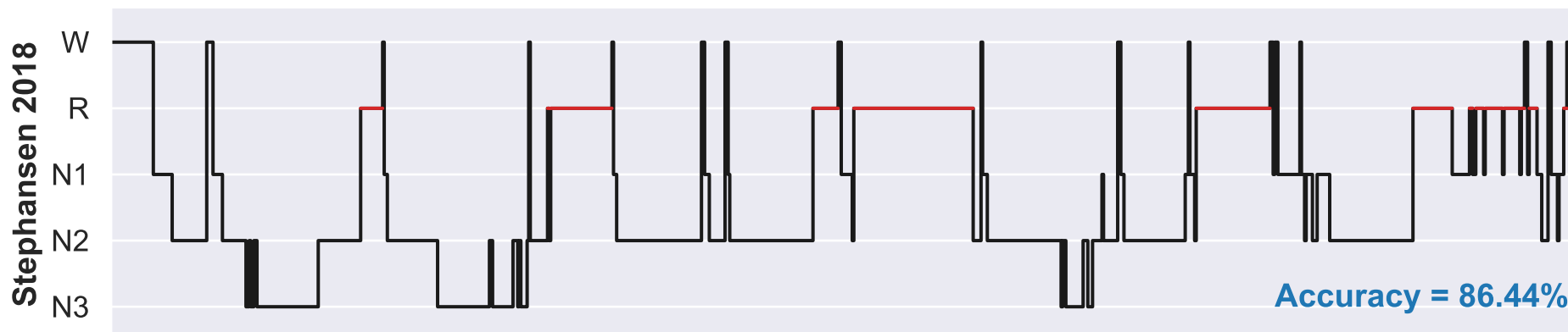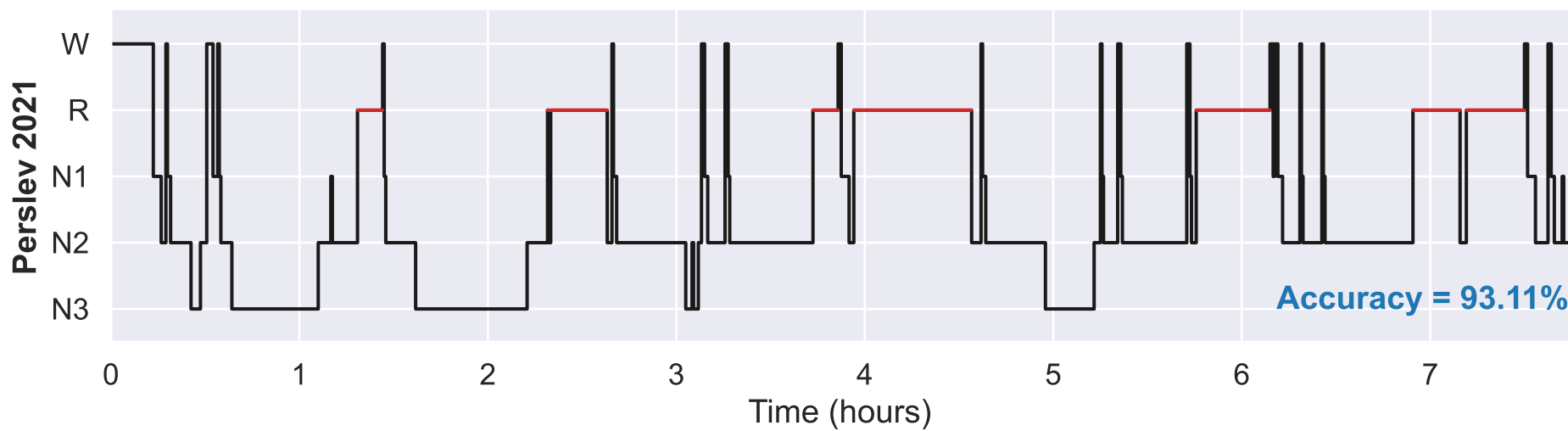

a3b1eed4

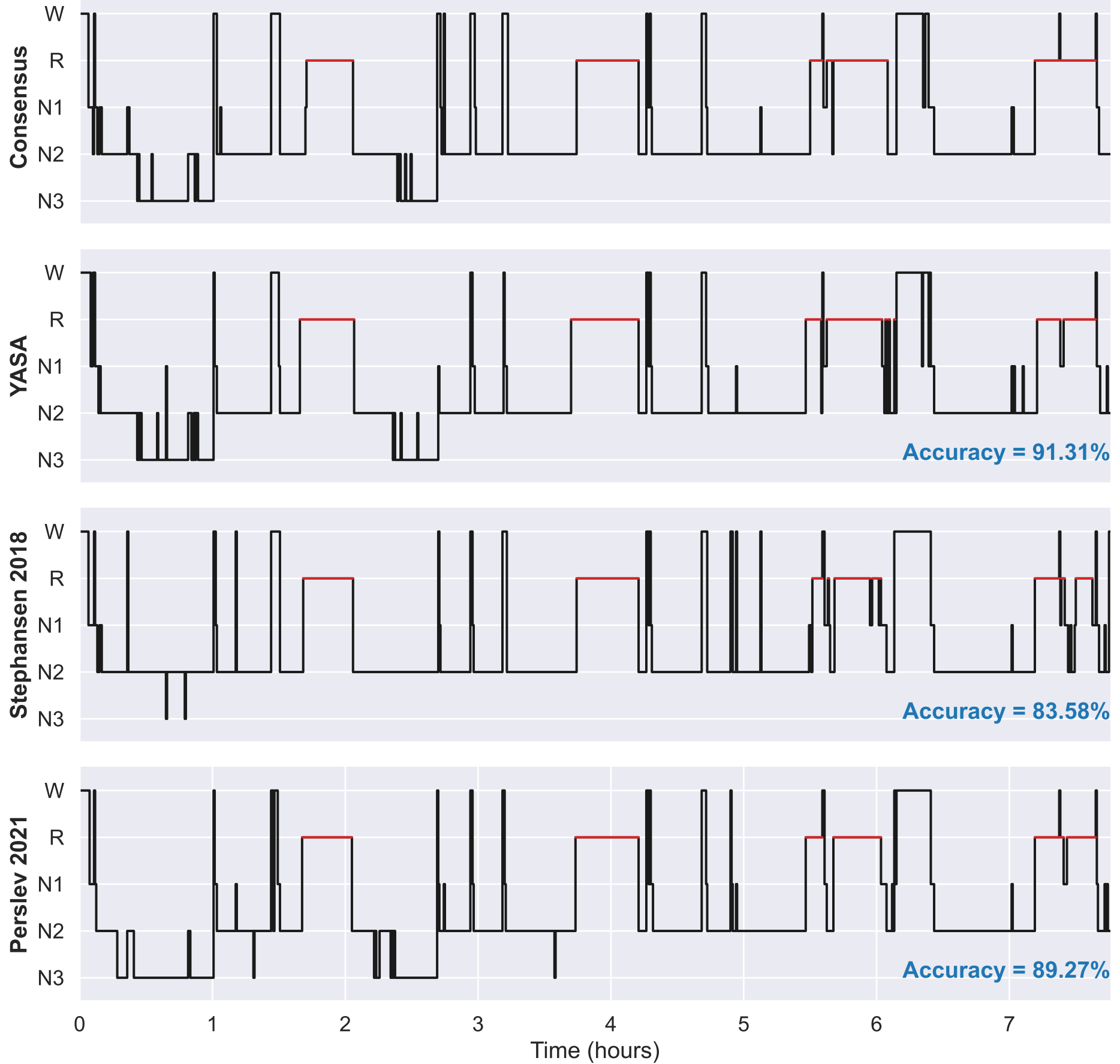

7d06e617

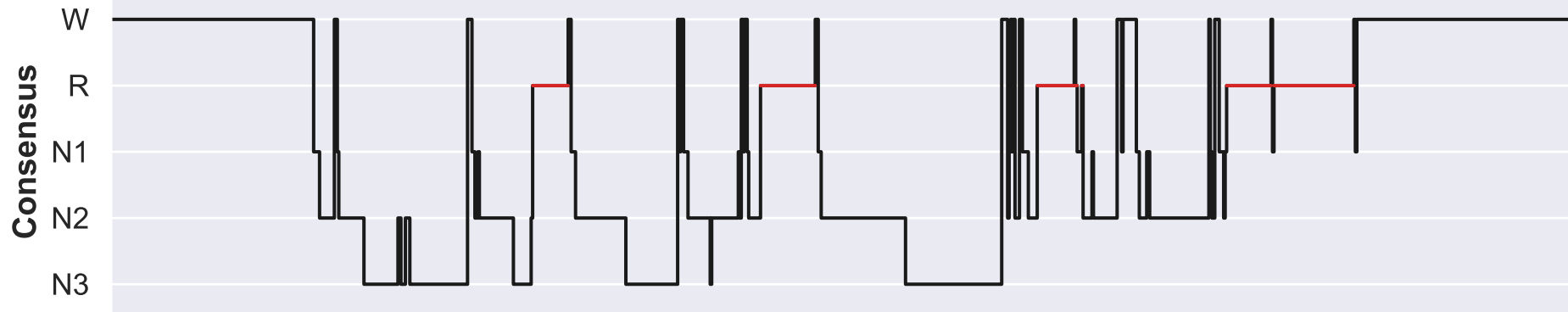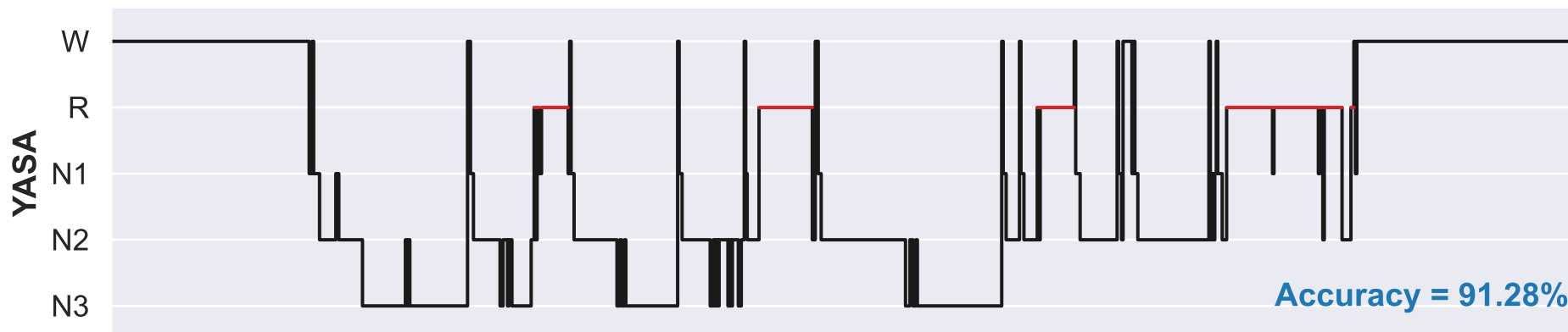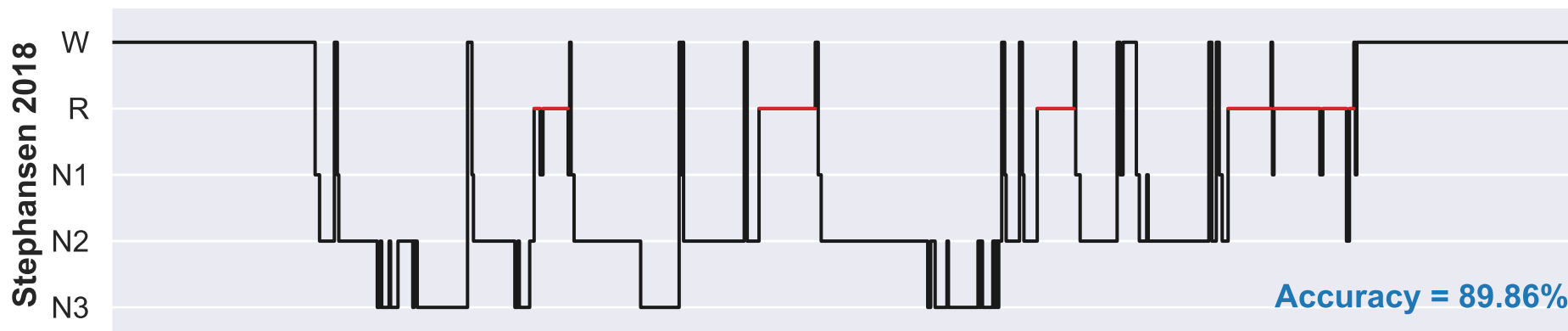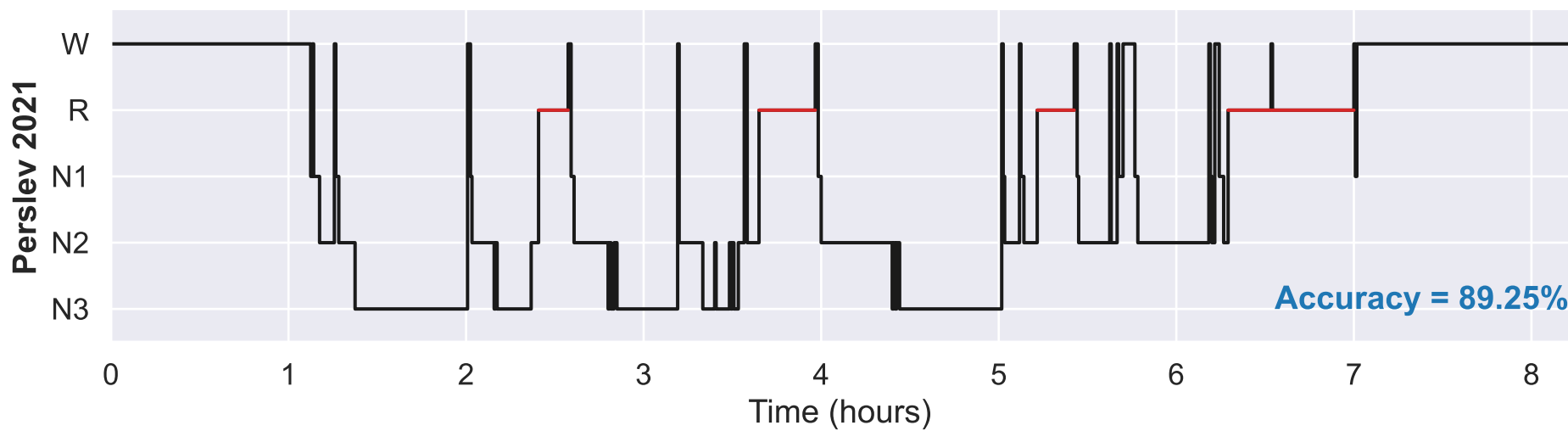

889dcc46

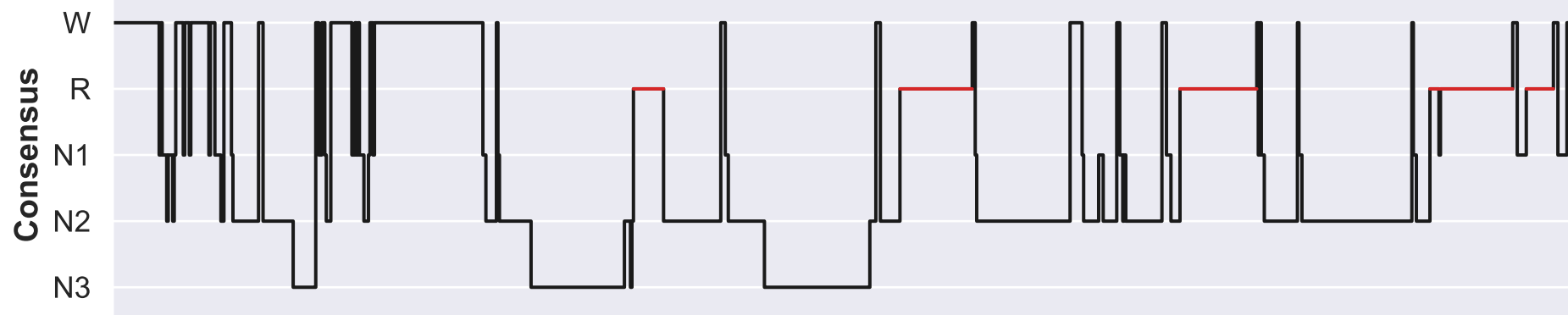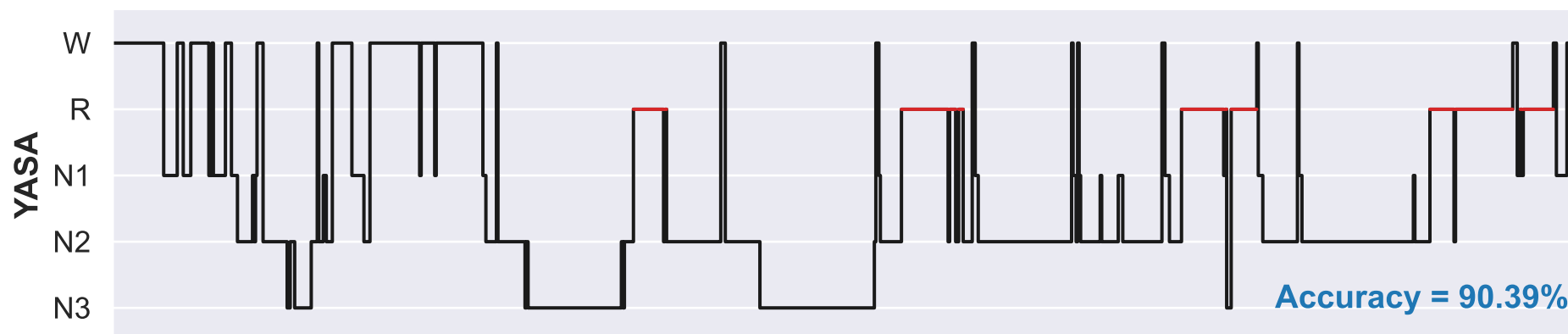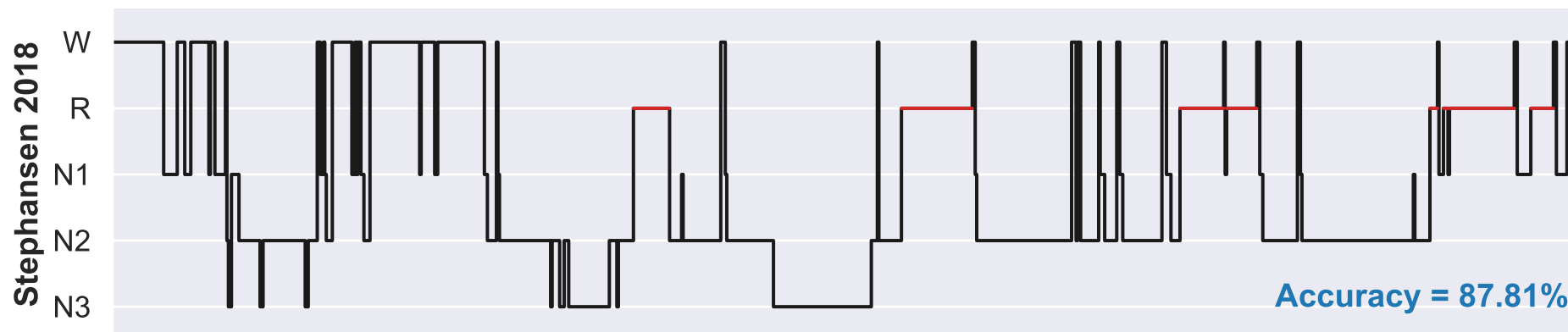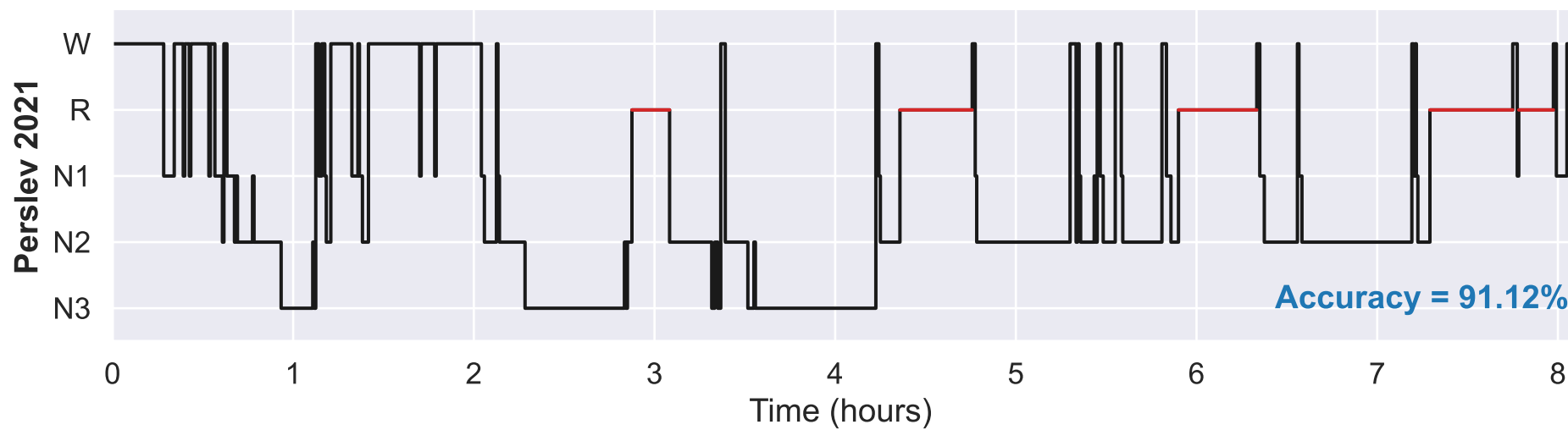

771ca402

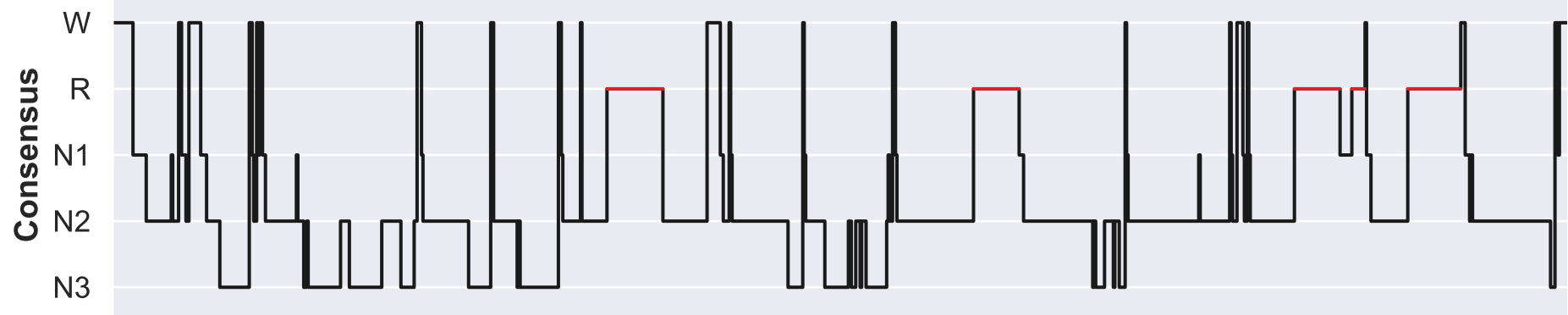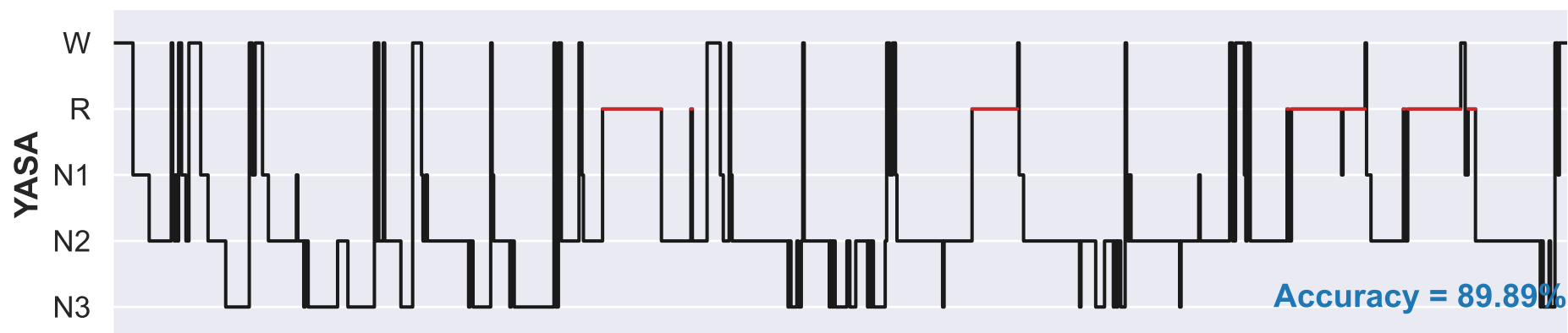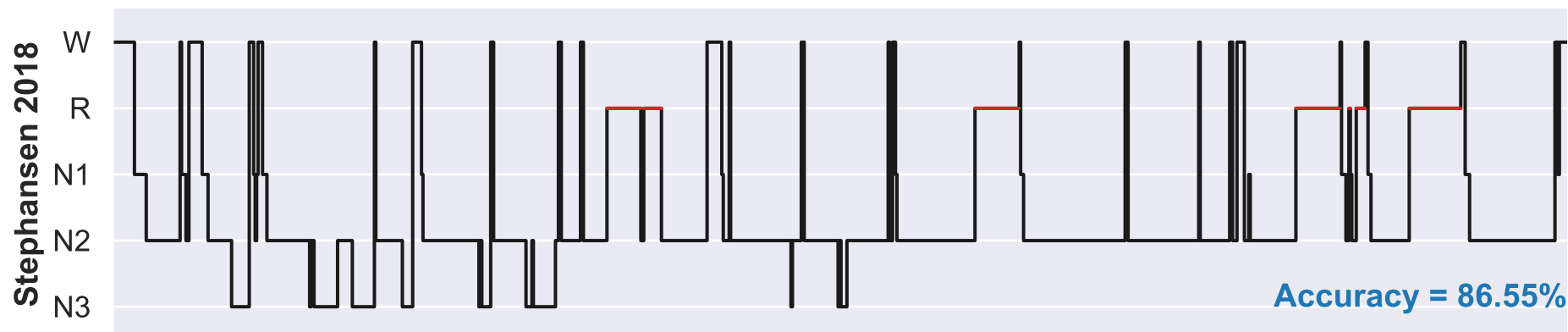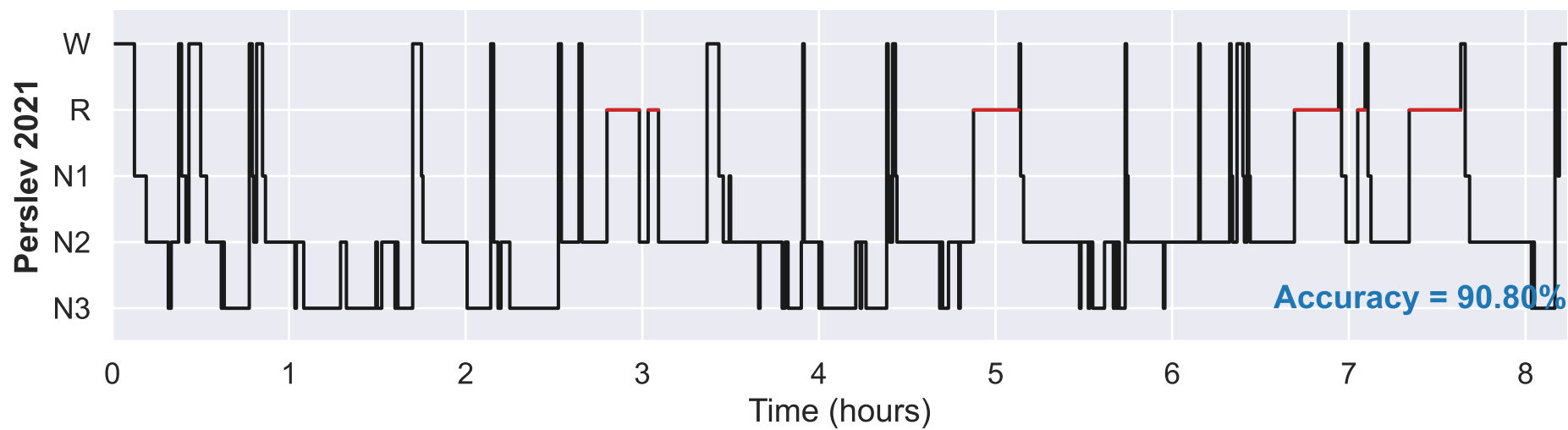

072653fc

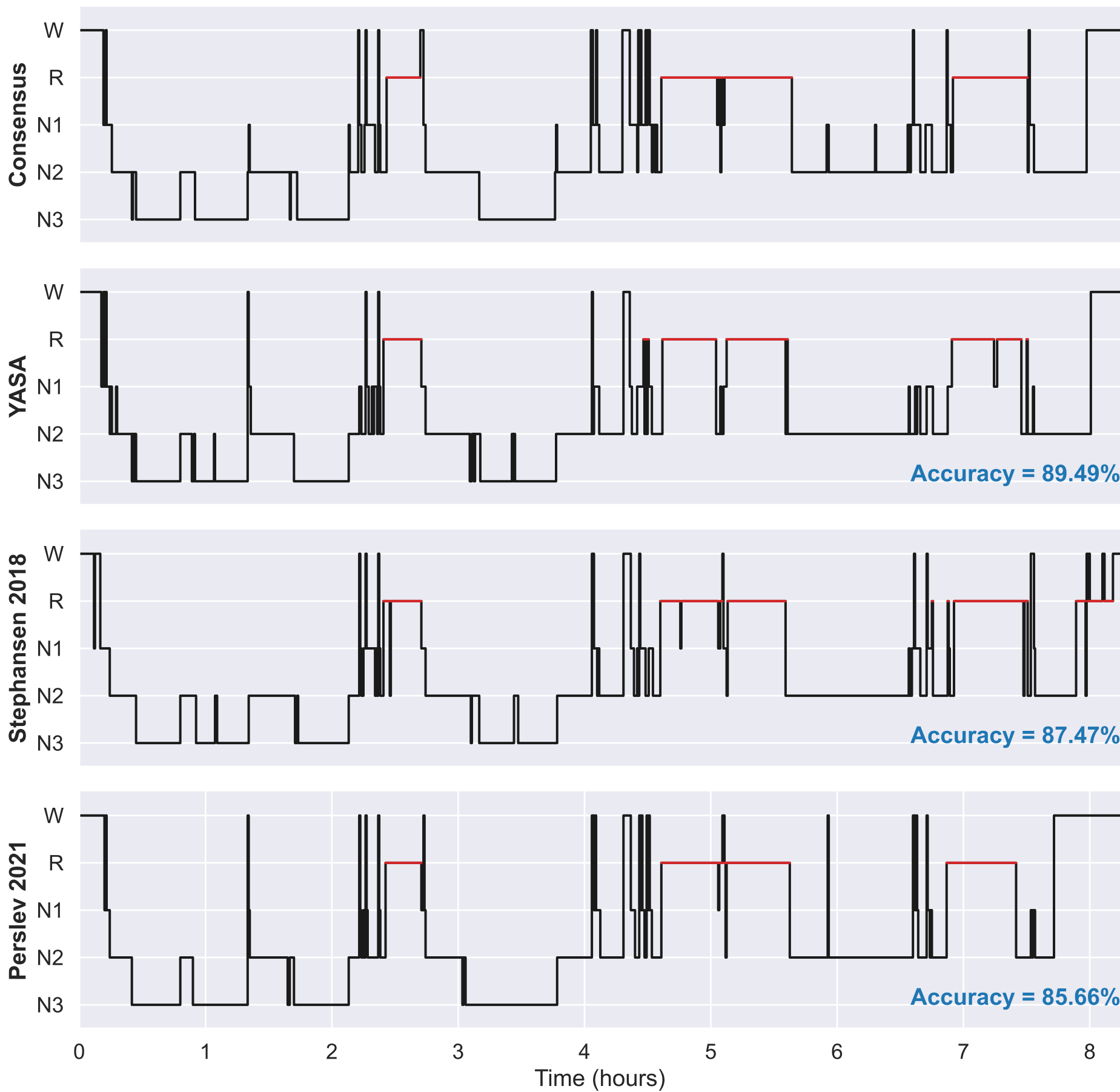

99603fdb

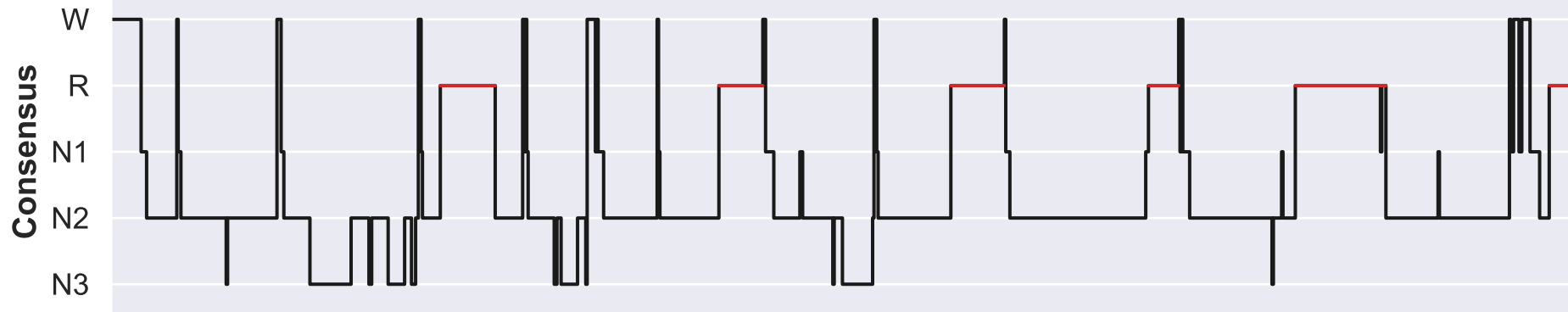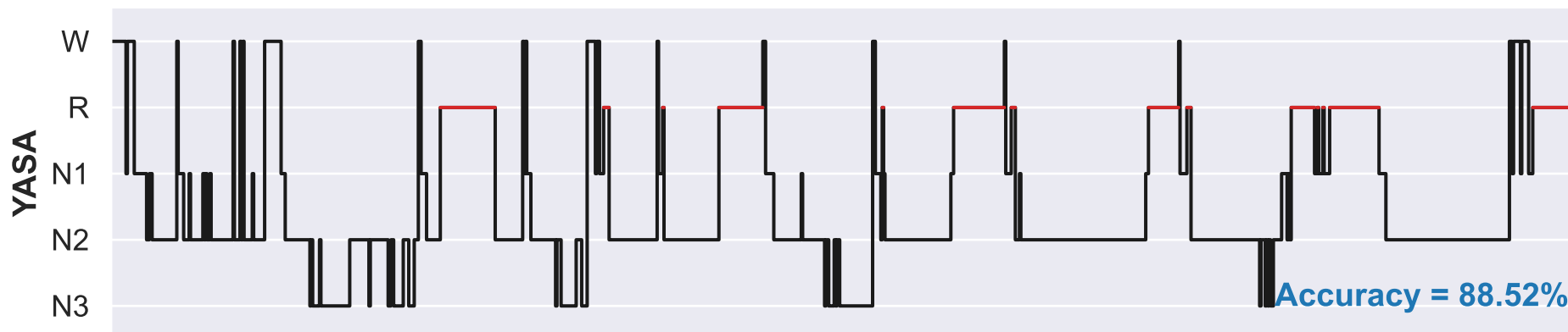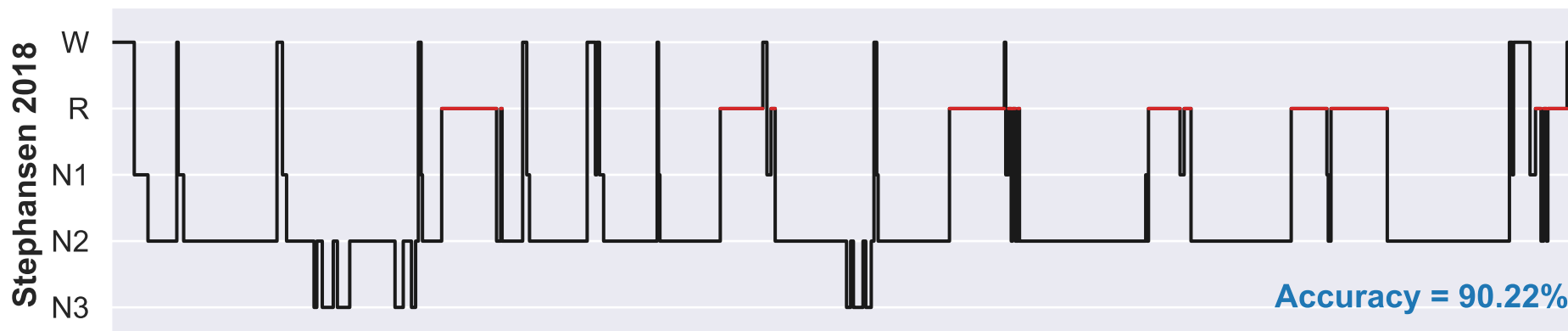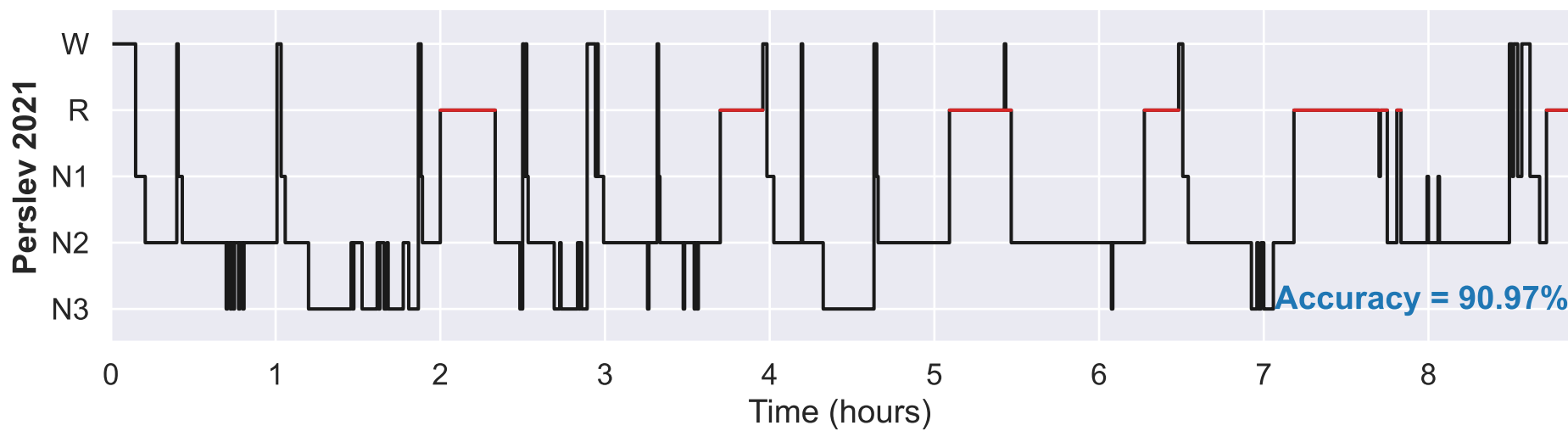

198c91b9

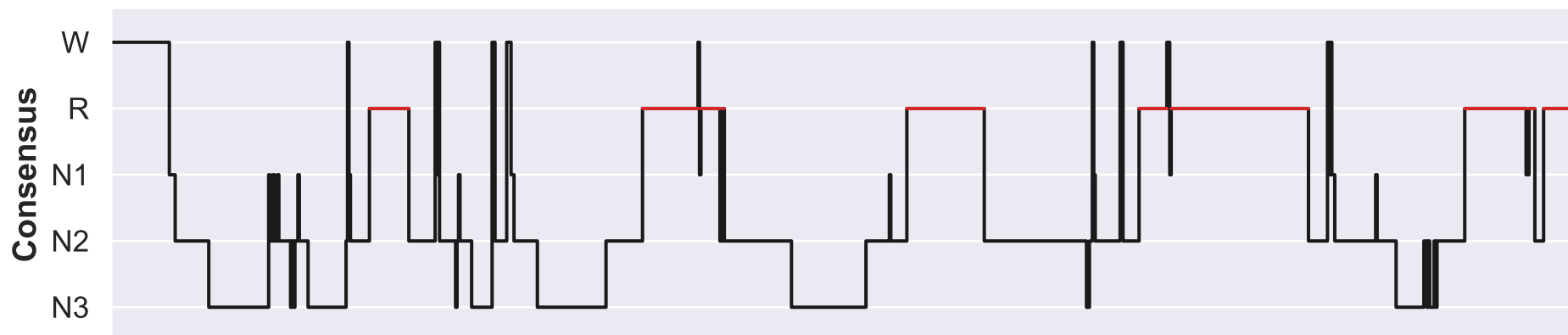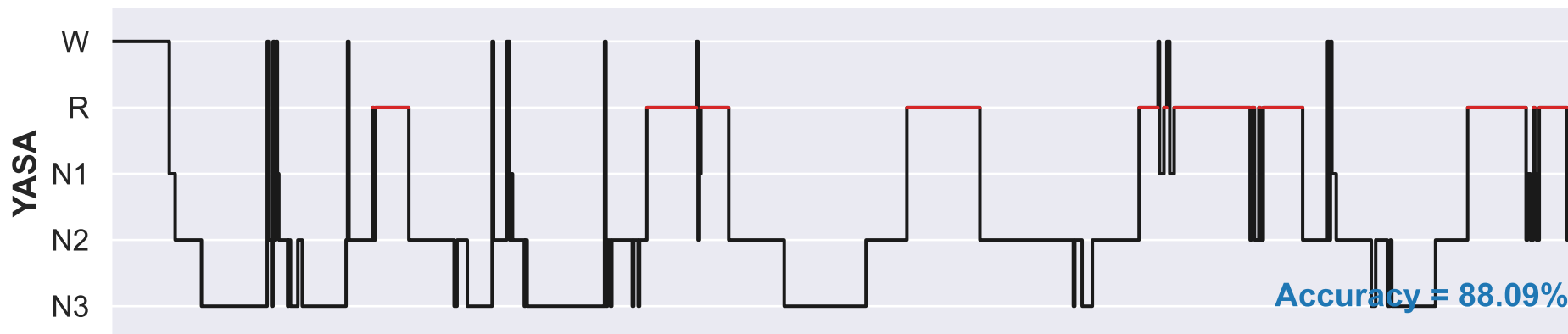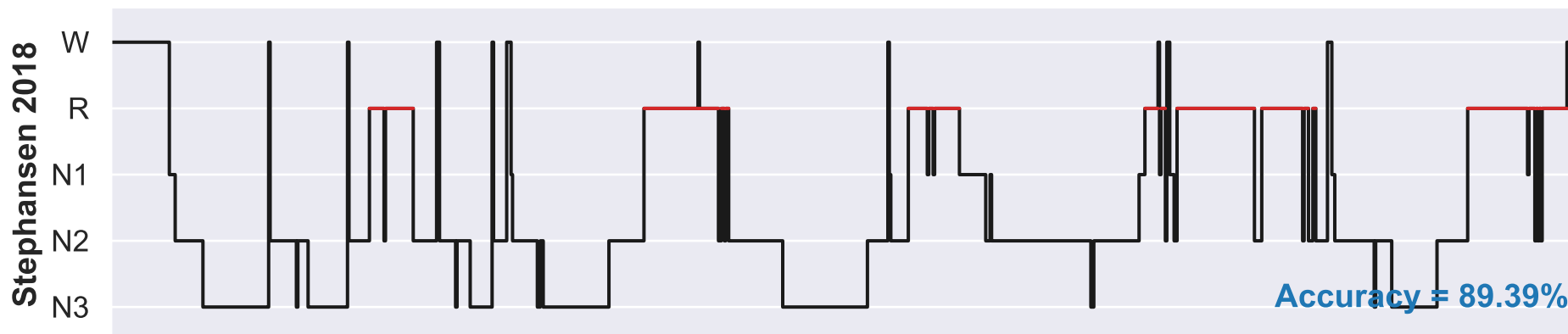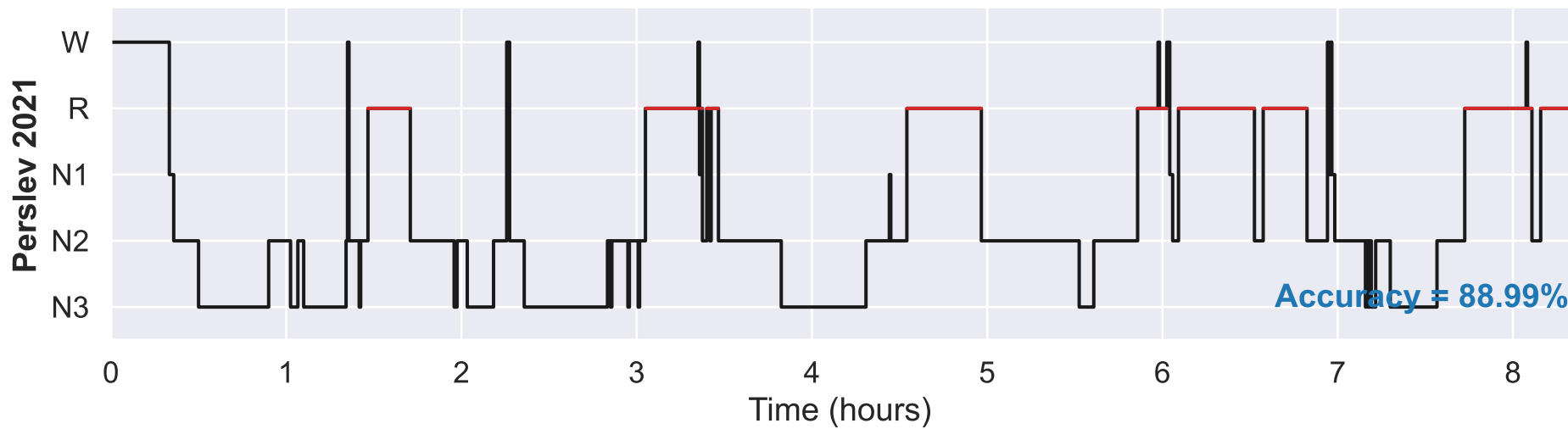

a14f8058

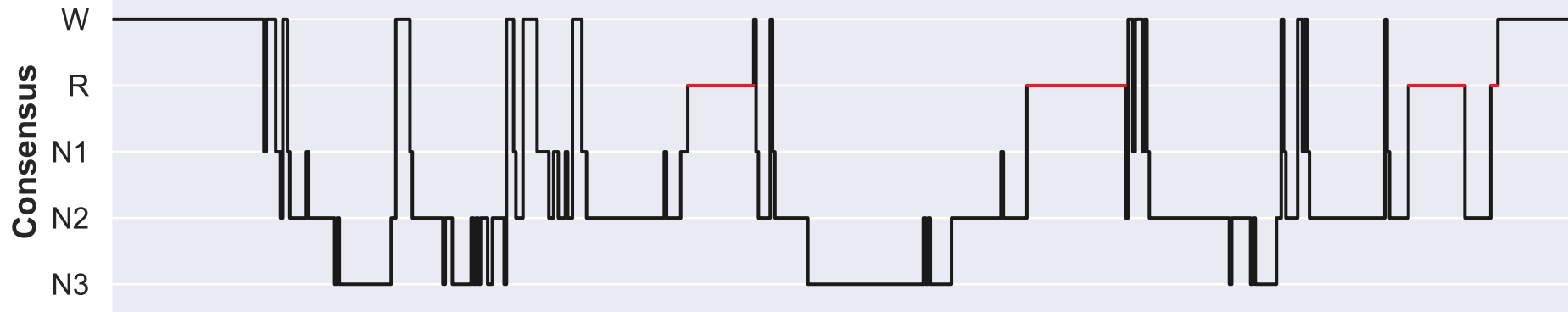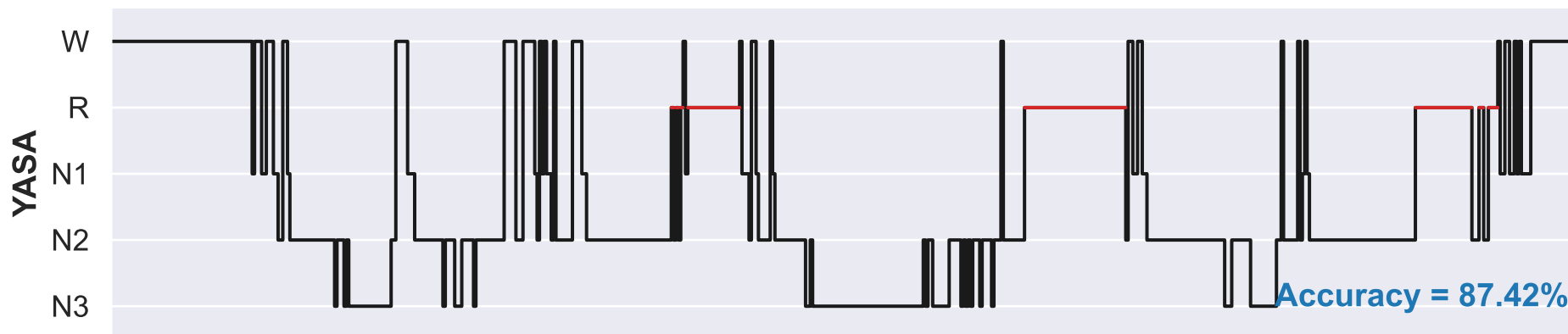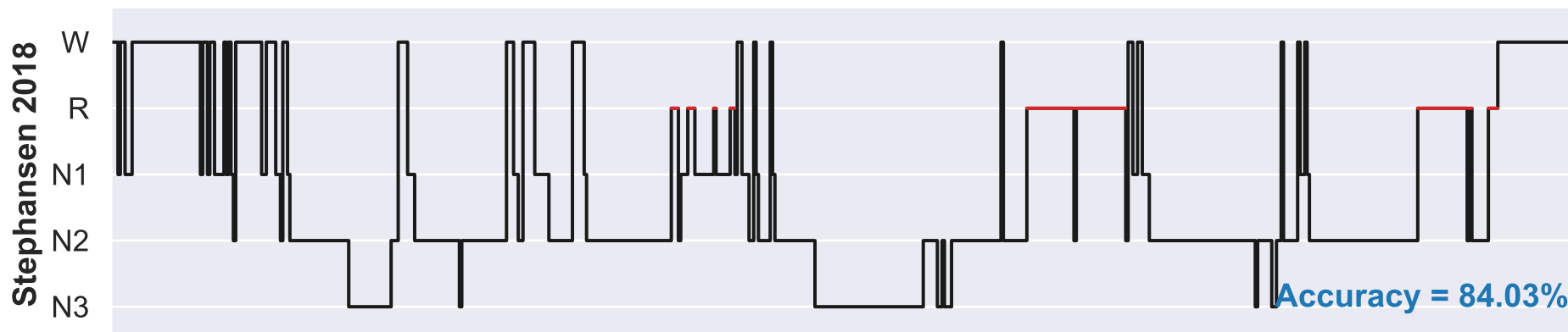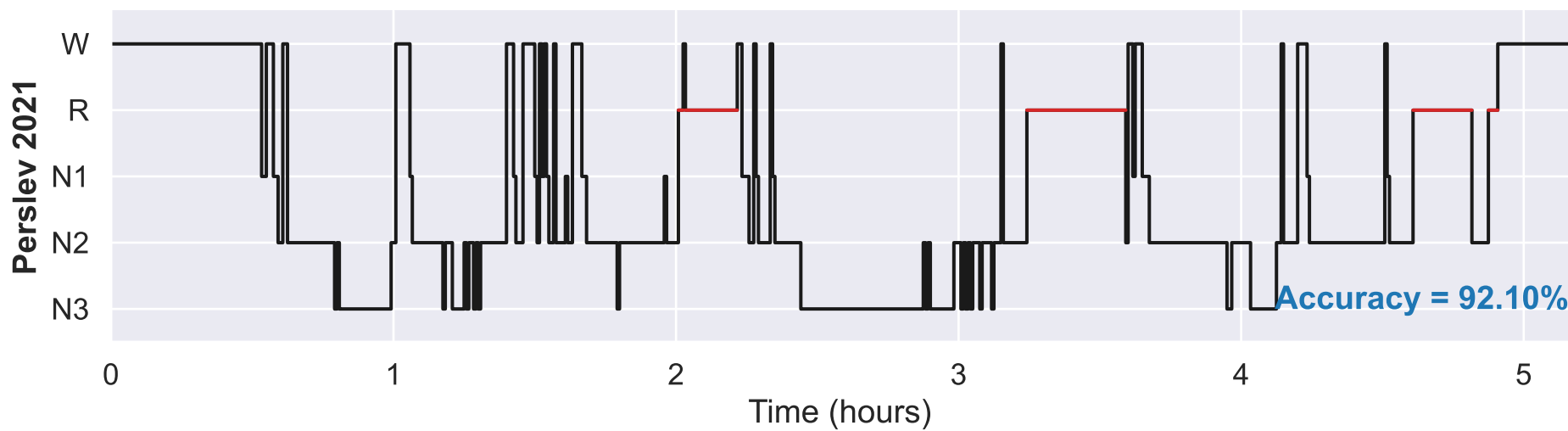

# 1340cbed

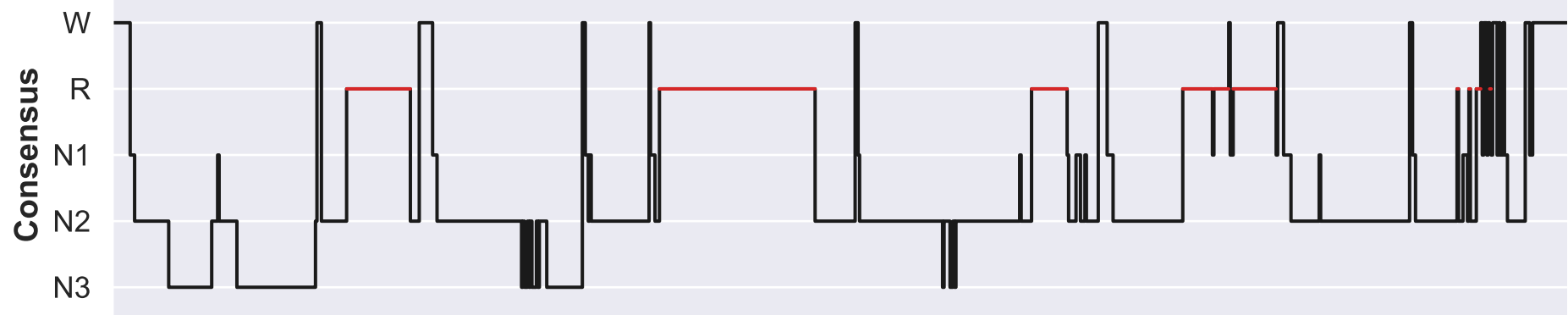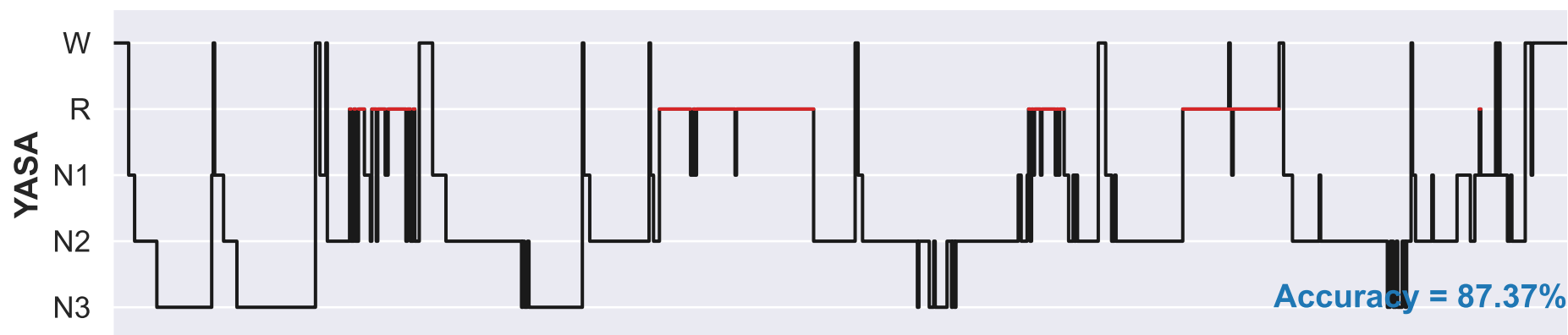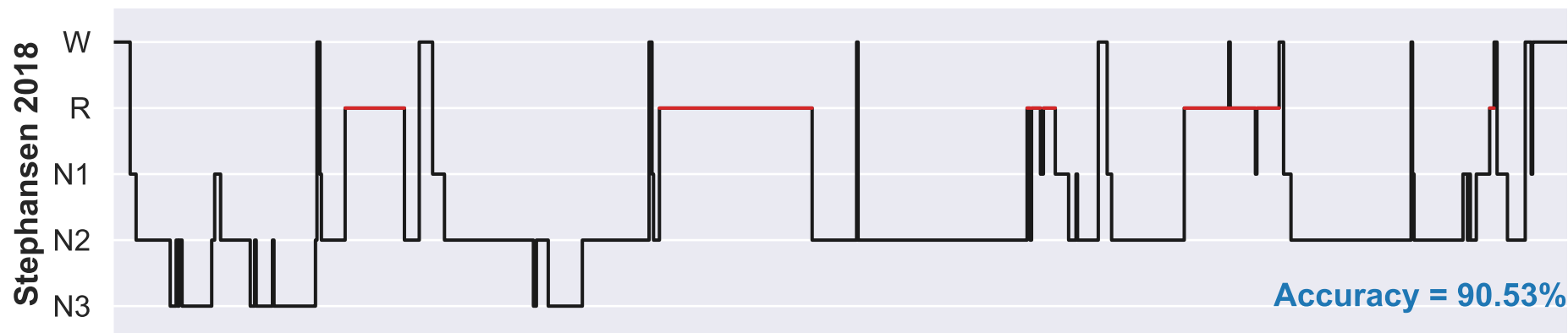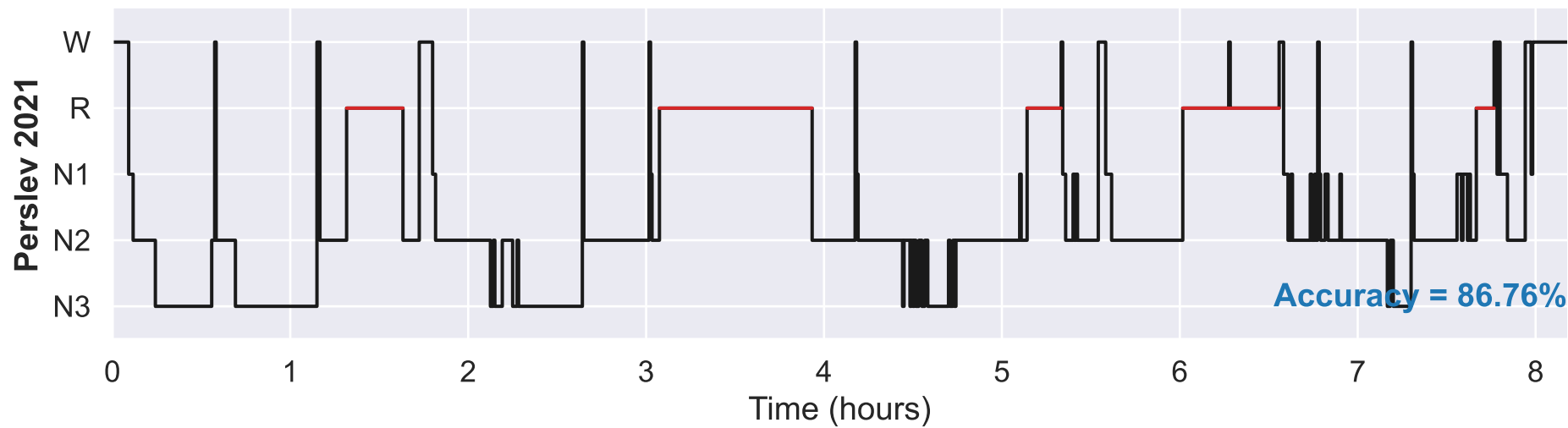

63b799f6

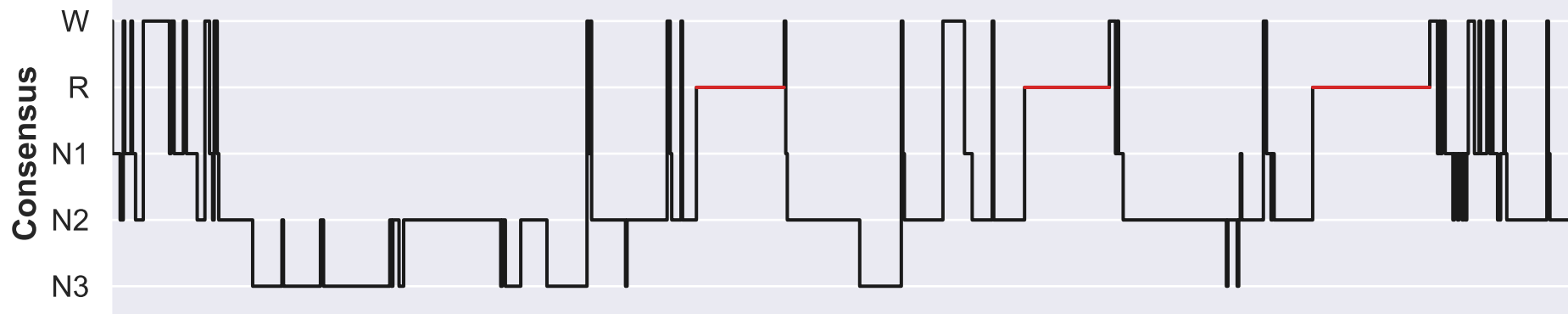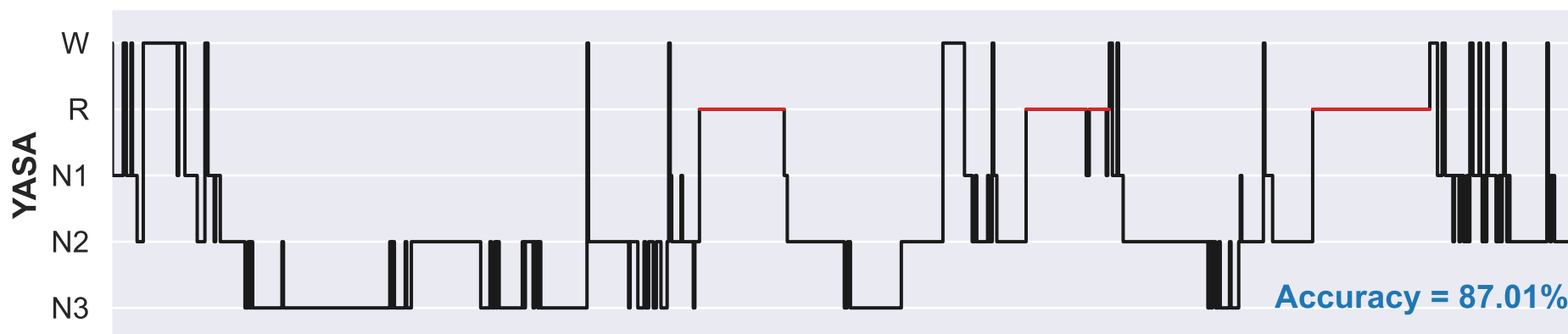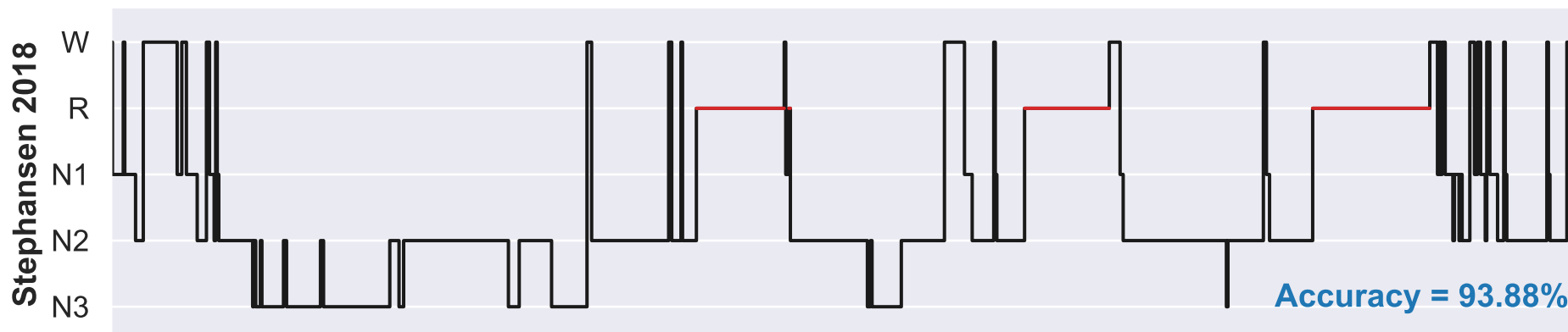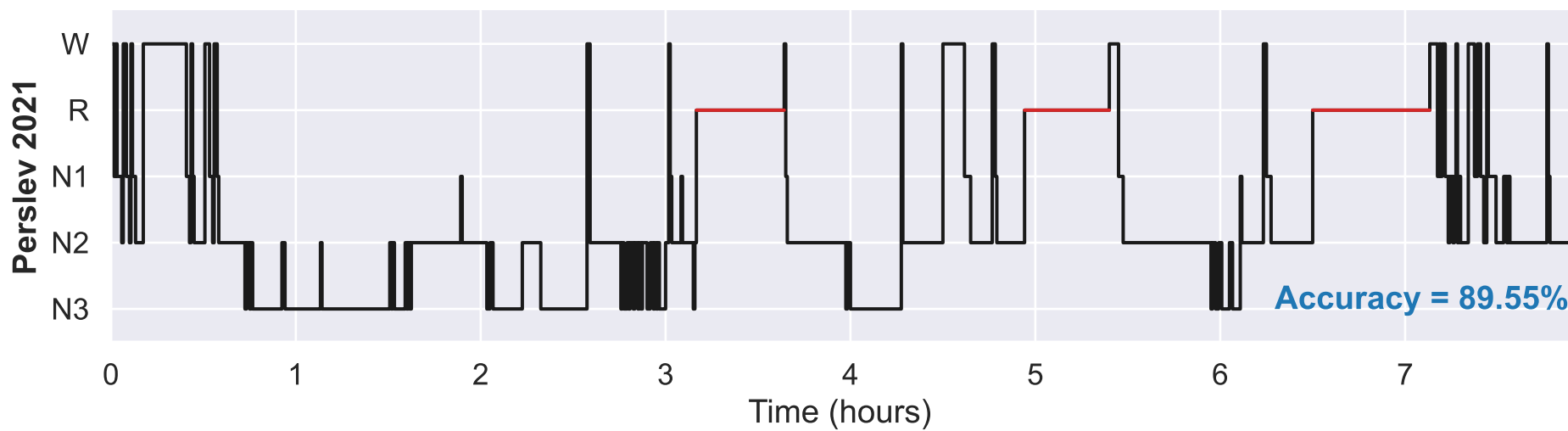

e00f7d62

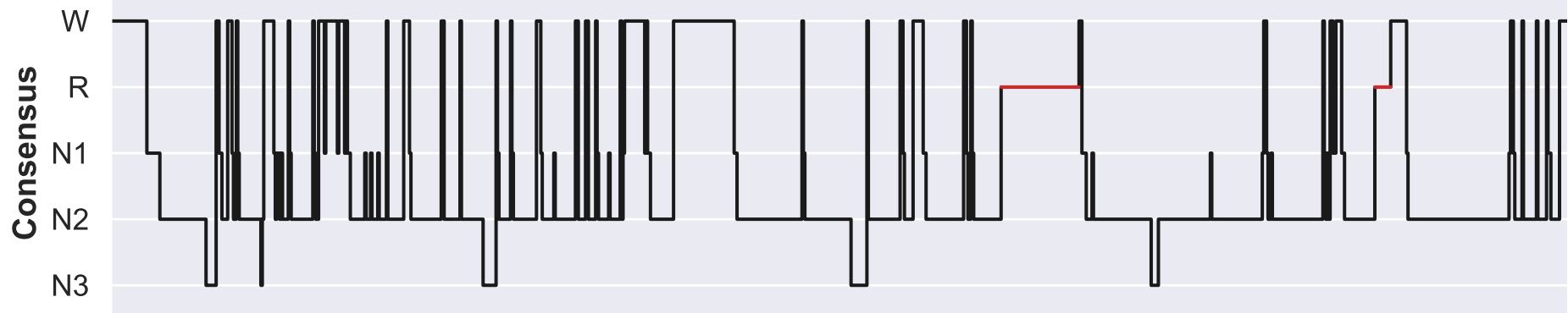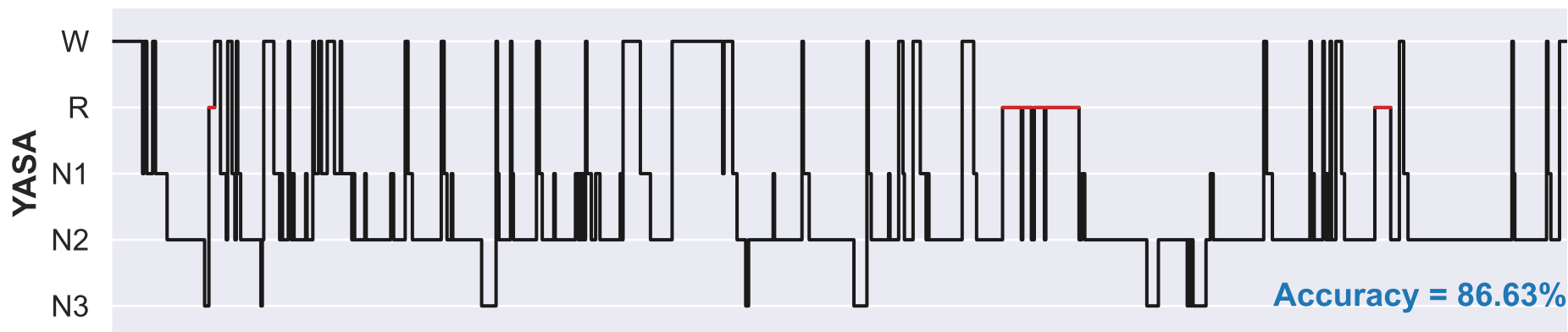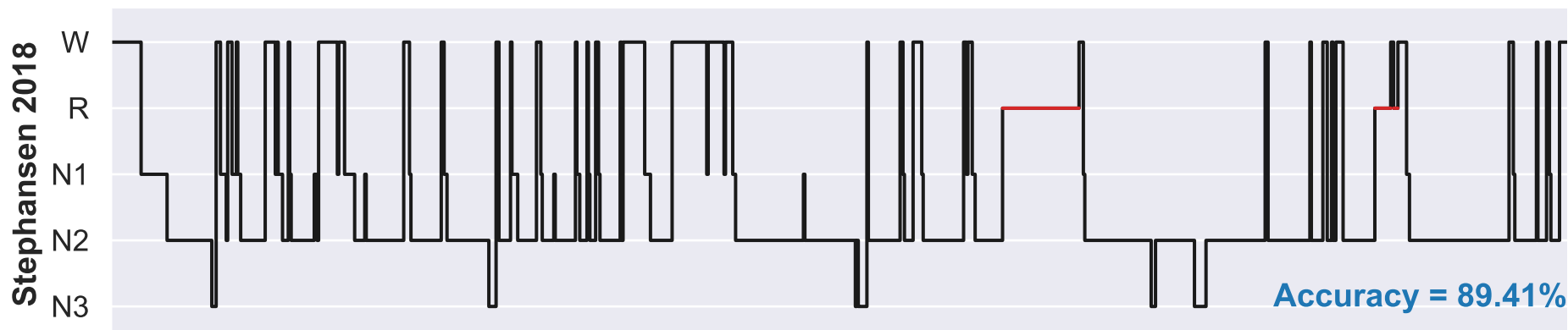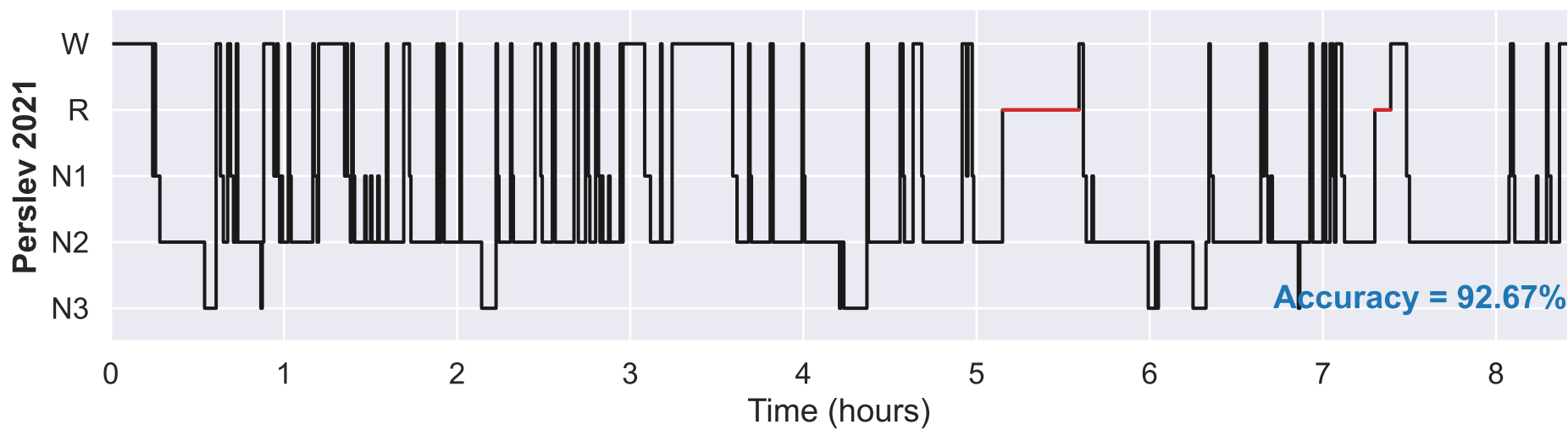

377ca73e

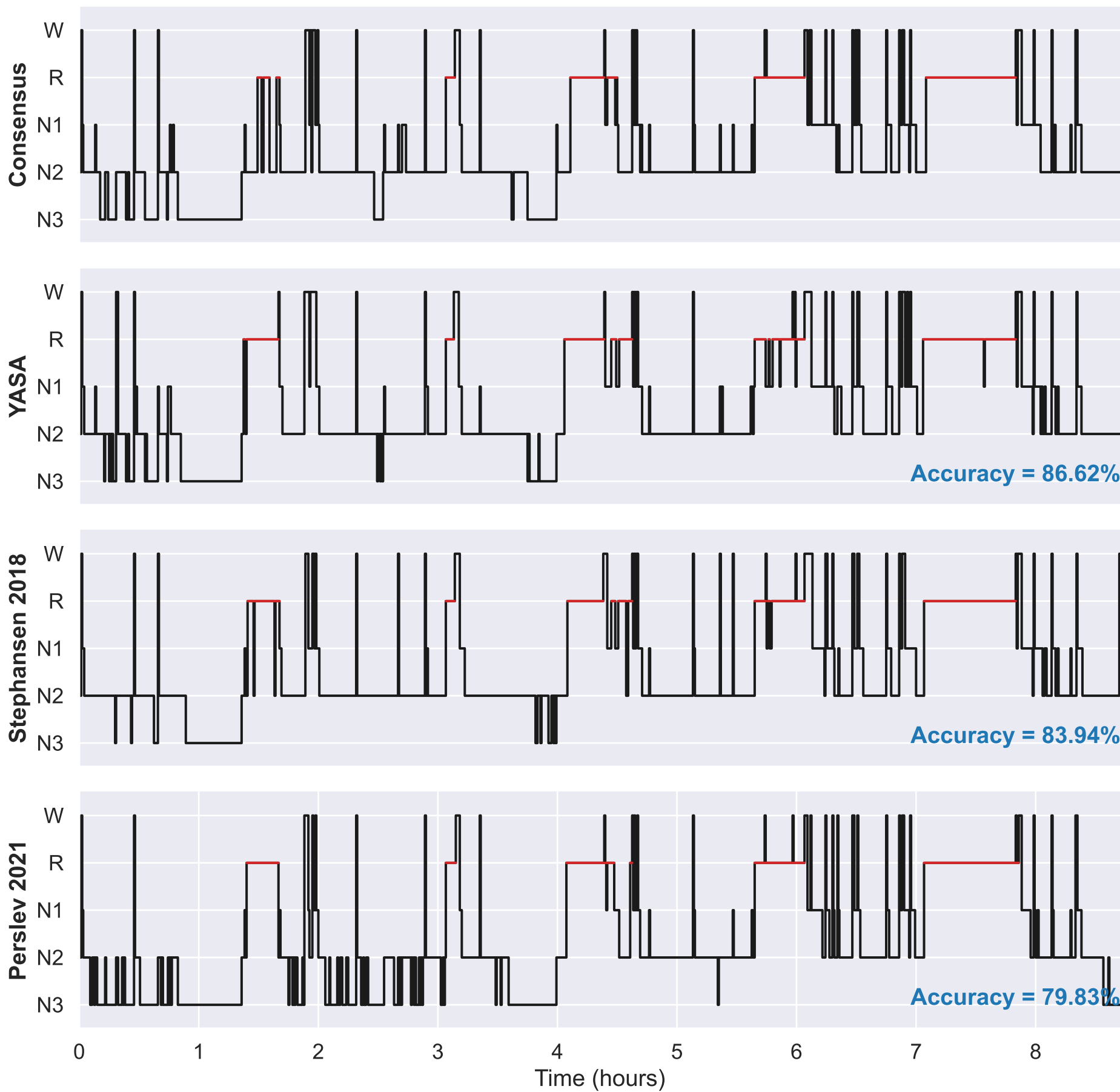

799df2d1

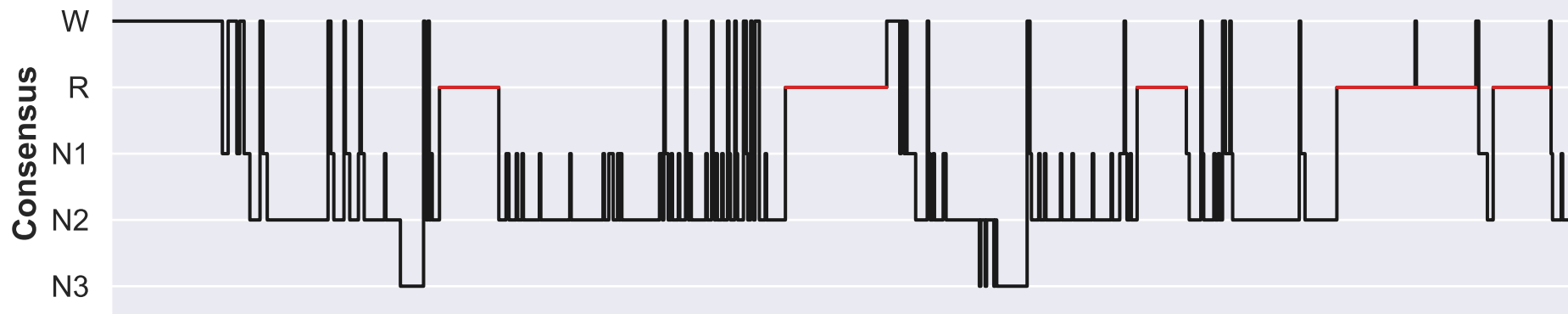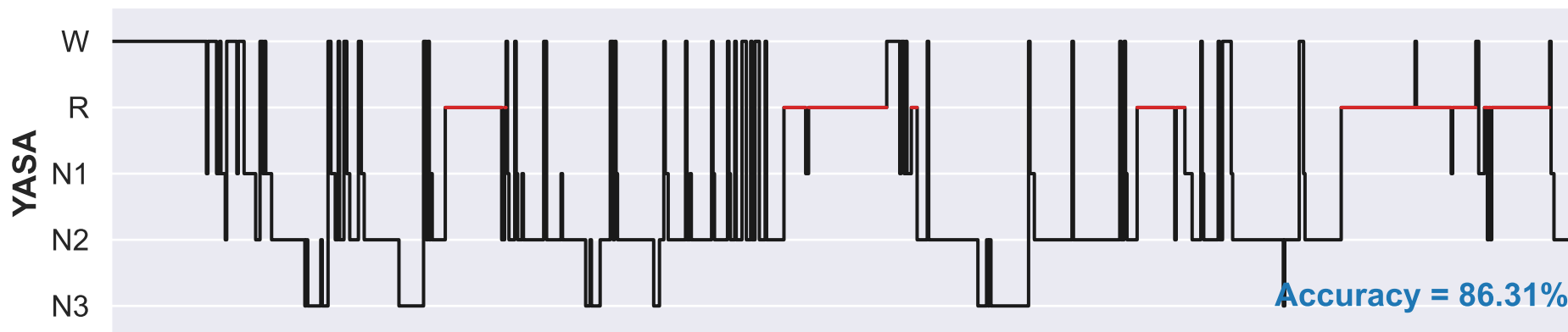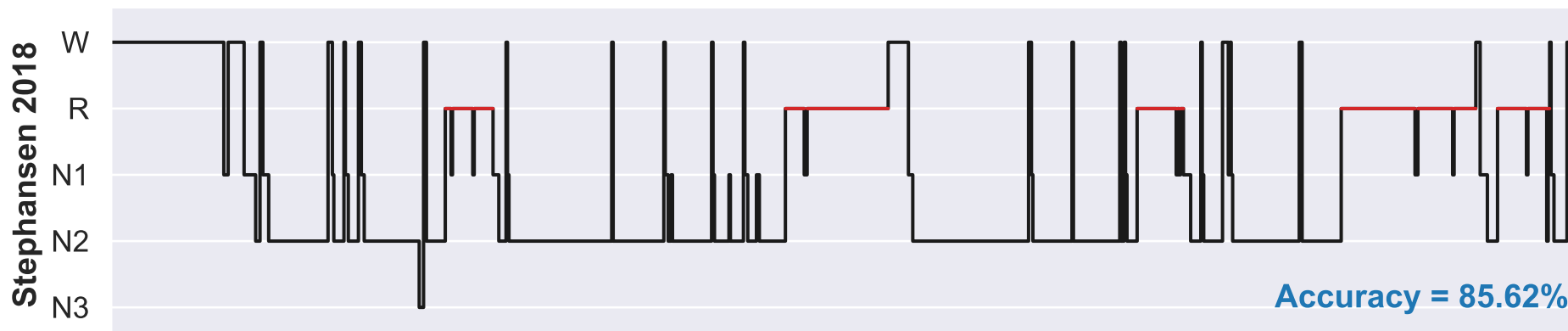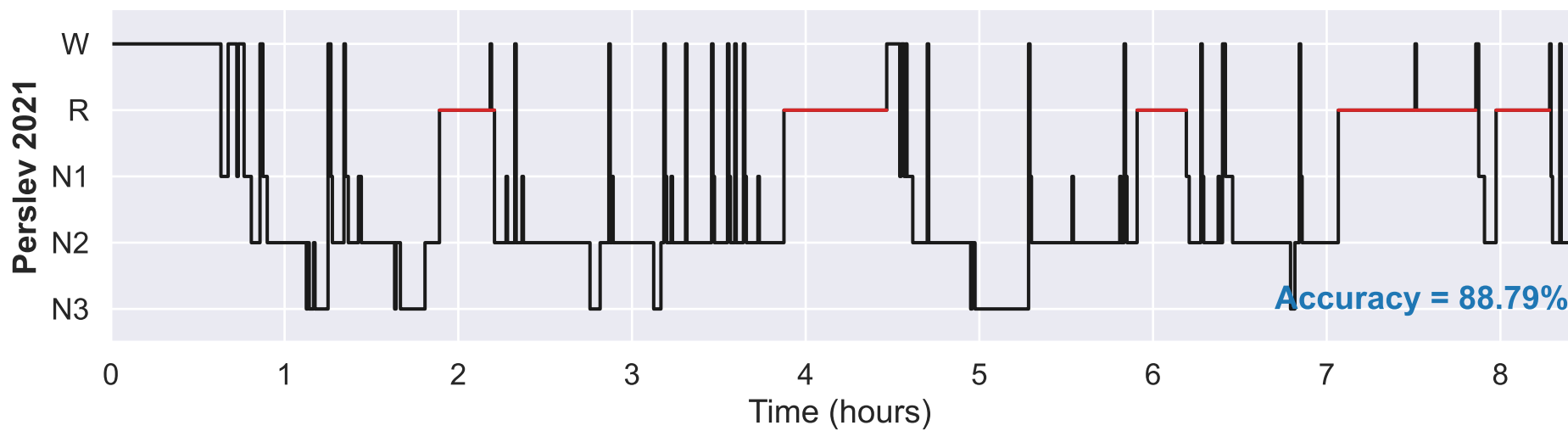

01e60017

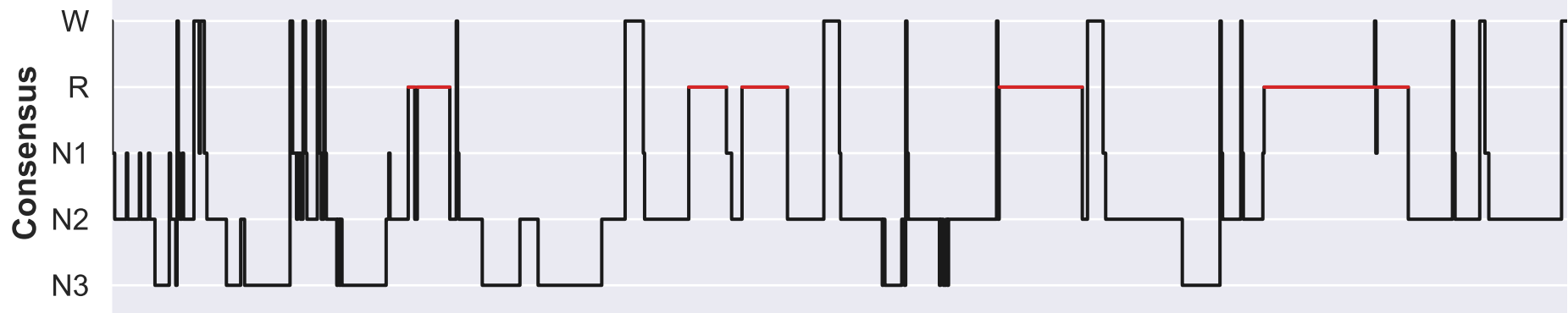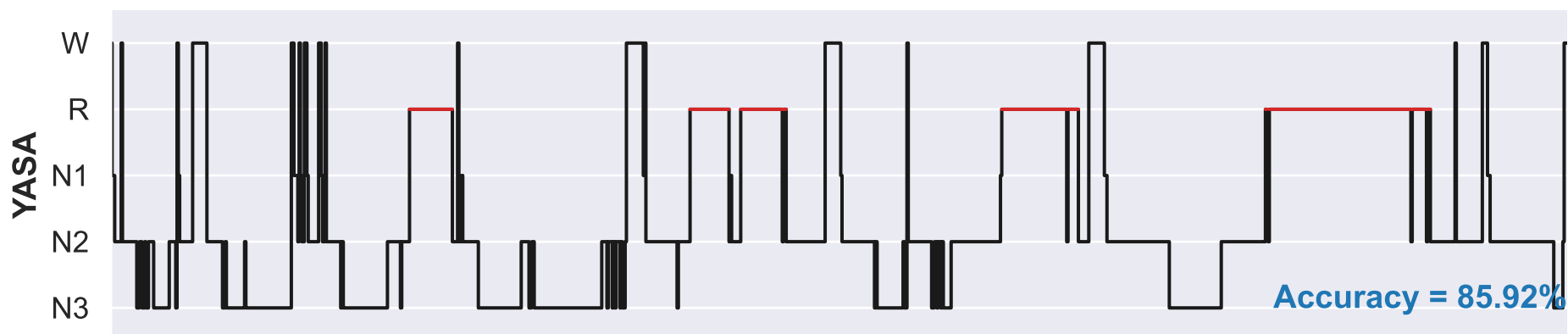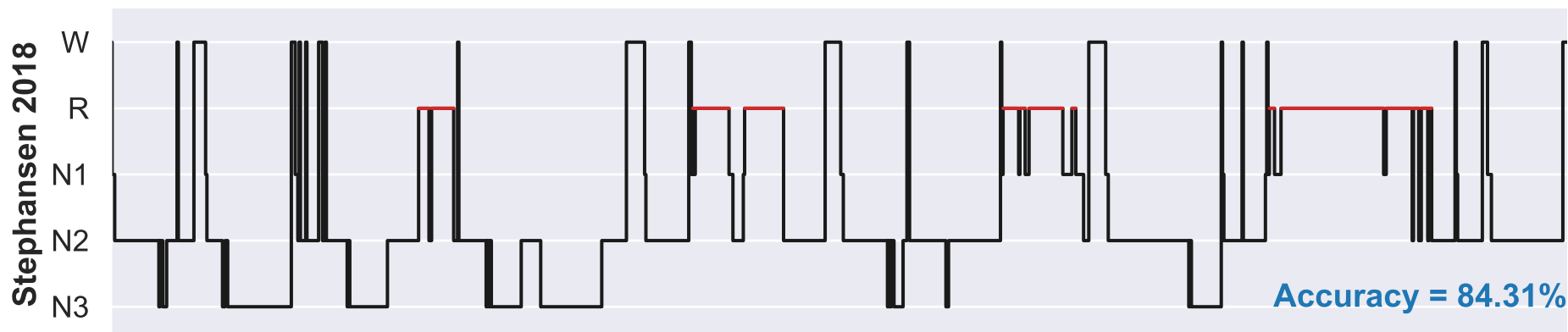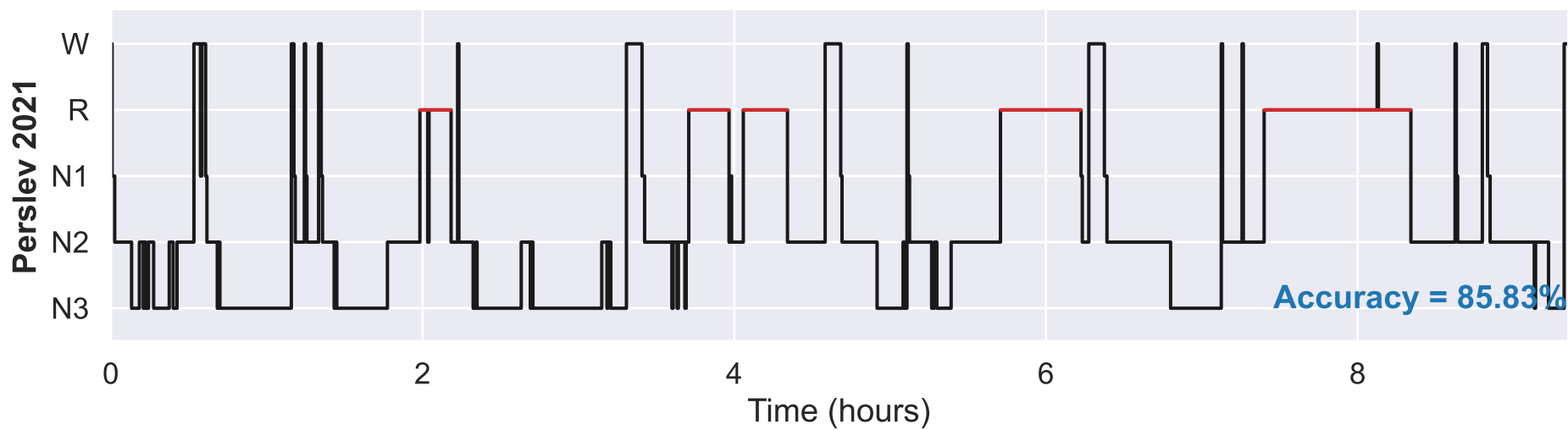

72e22310

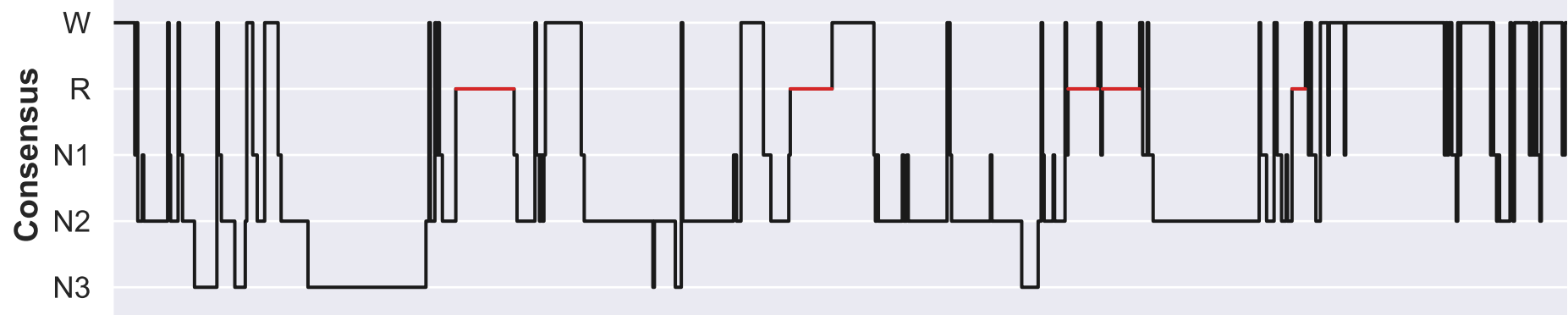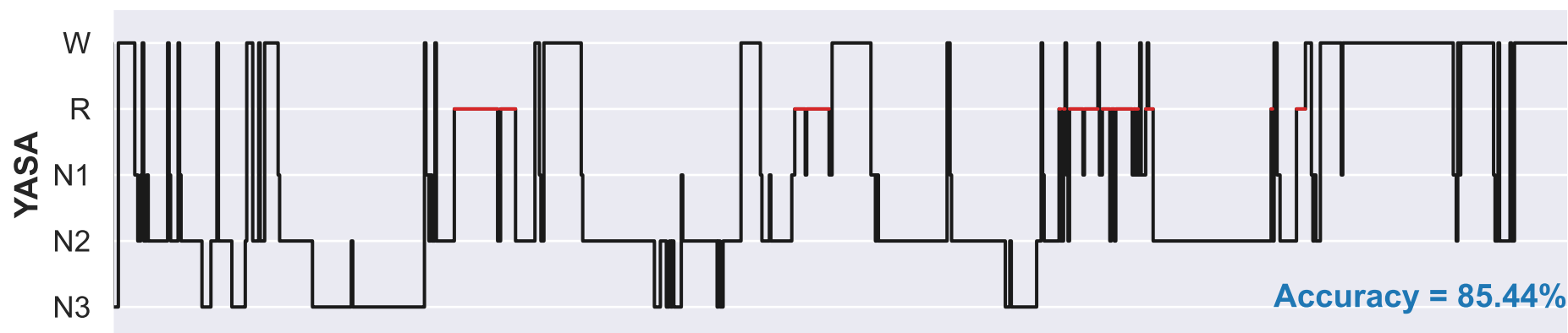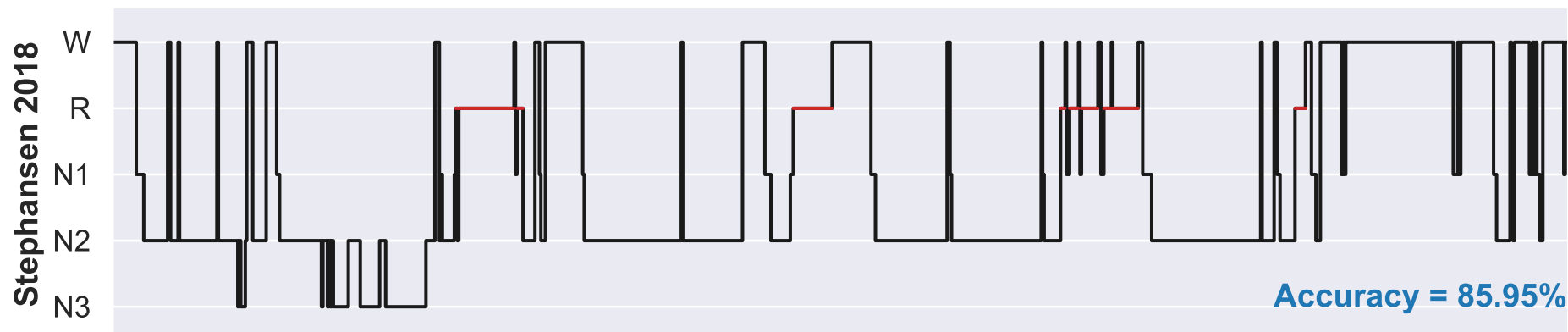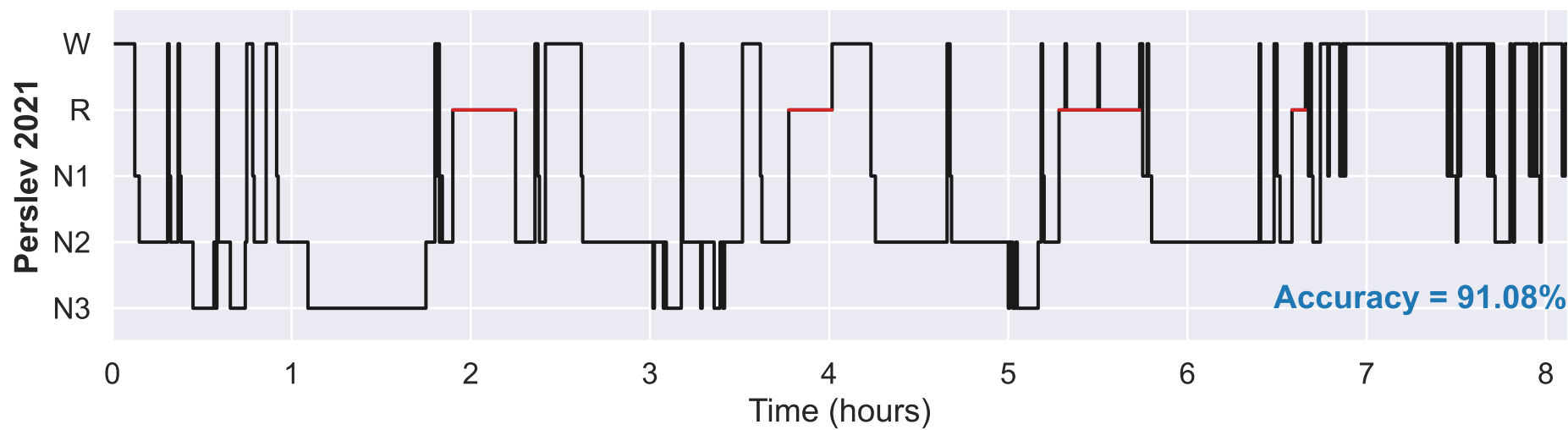

e055b5a6

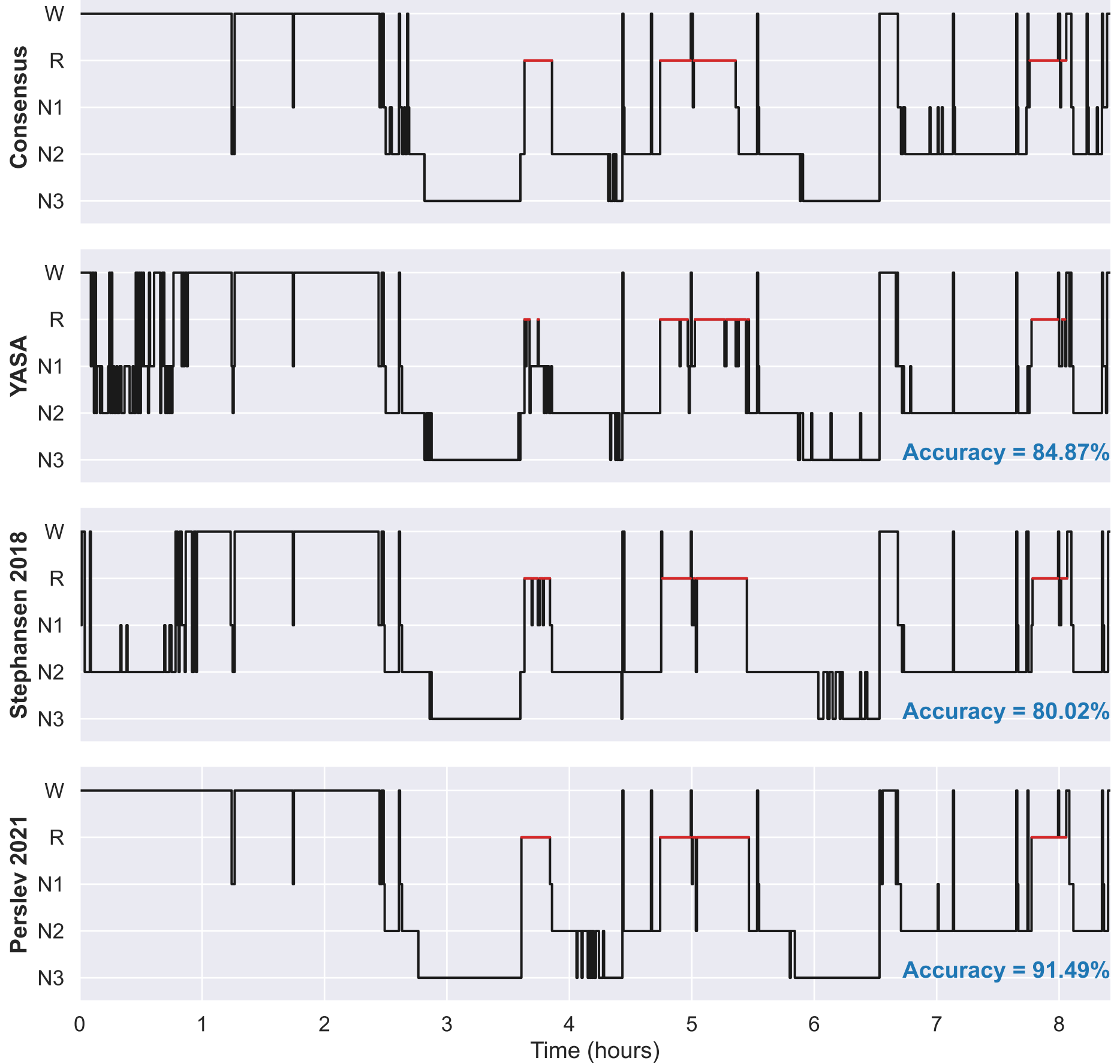

42f25159

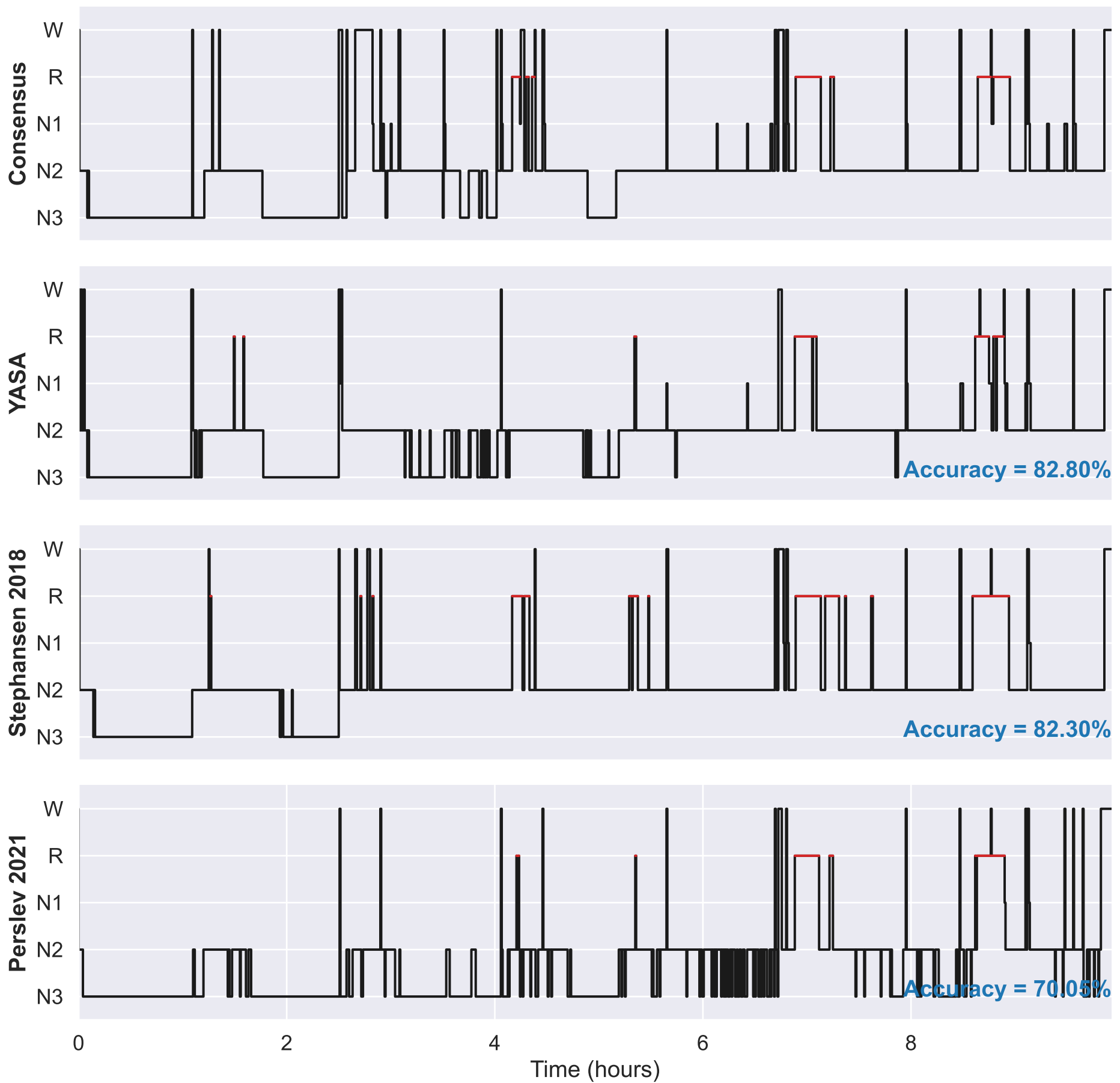

de3af7b1

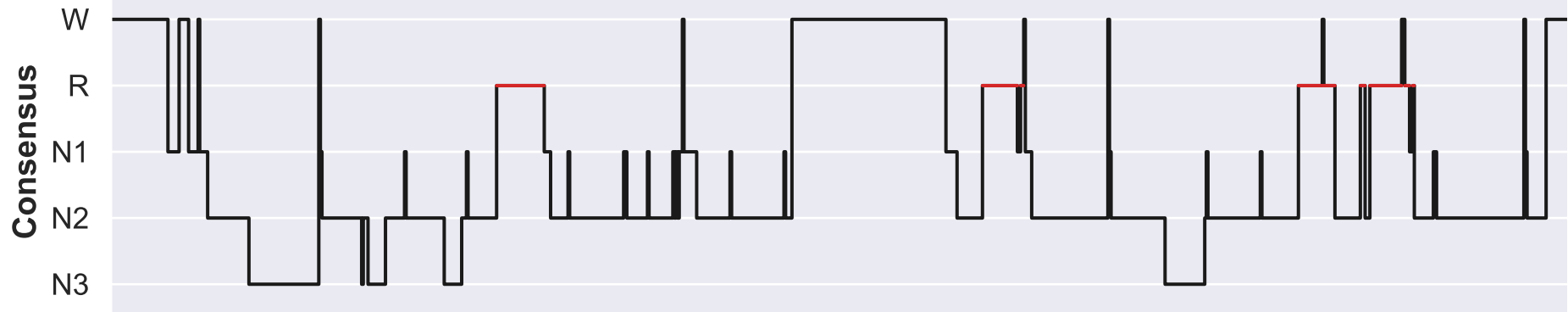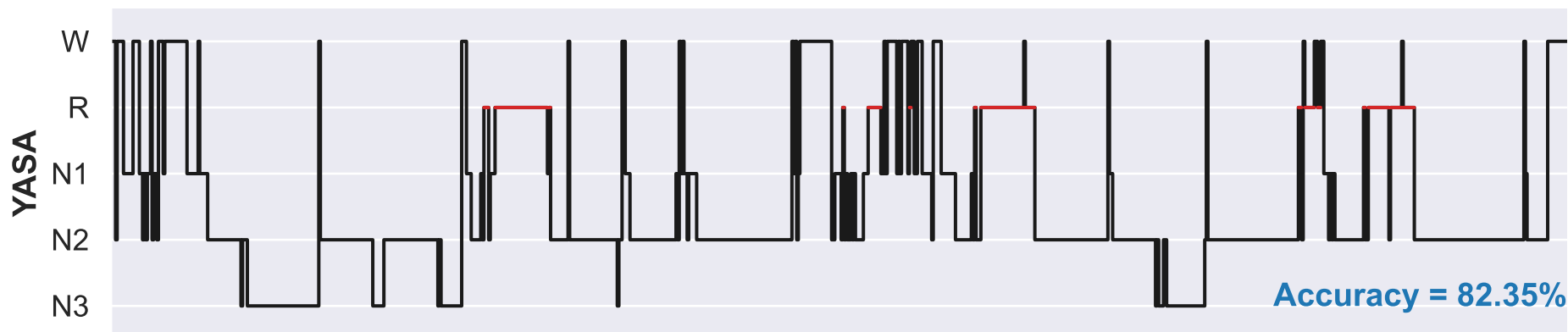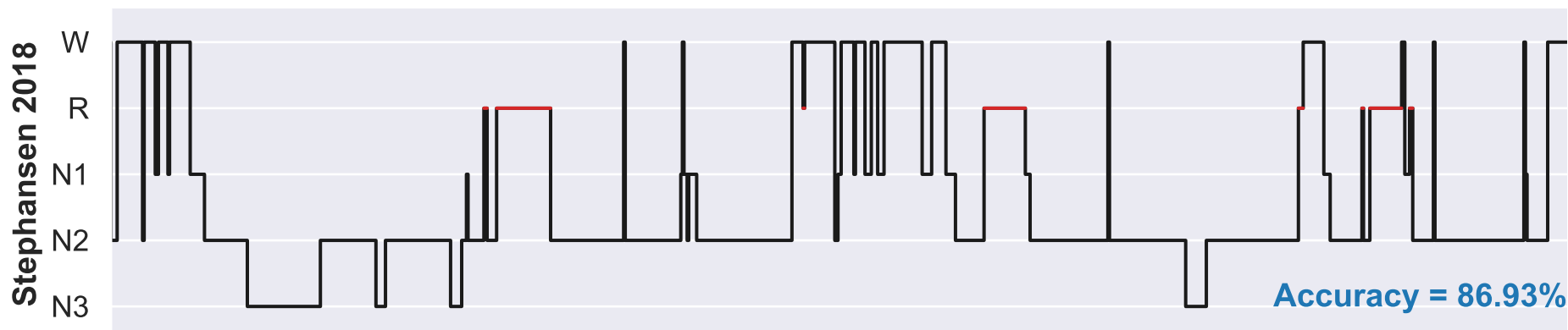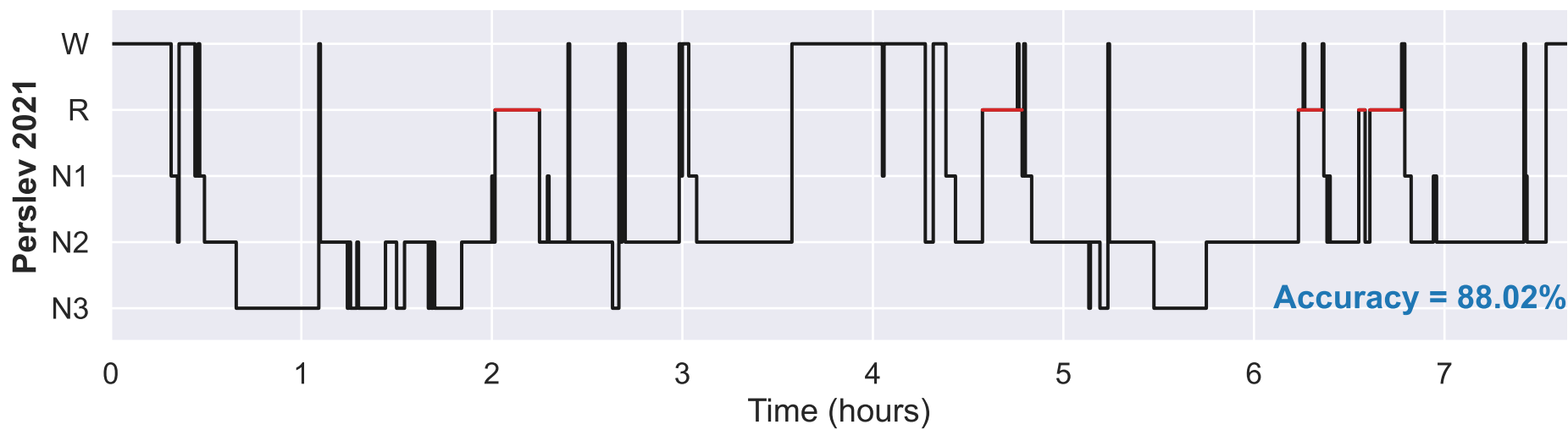

5d01f8da

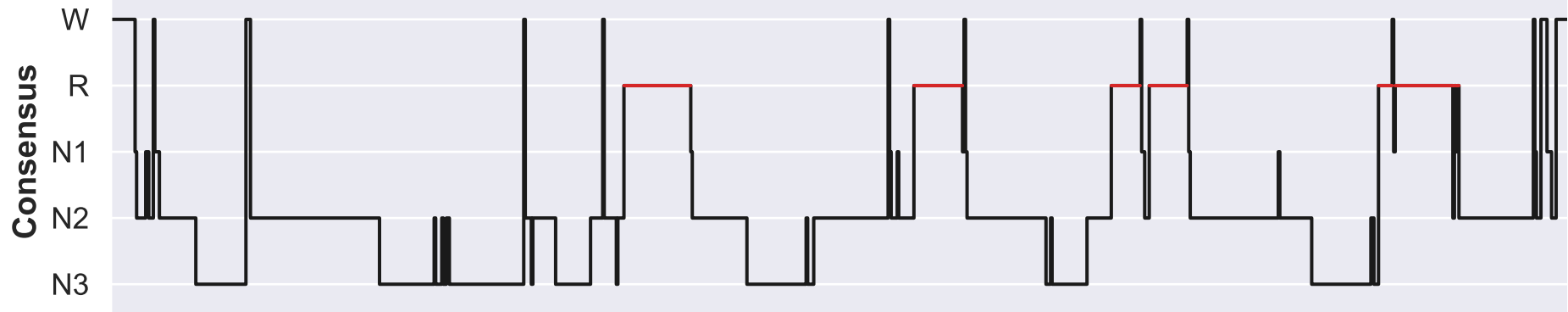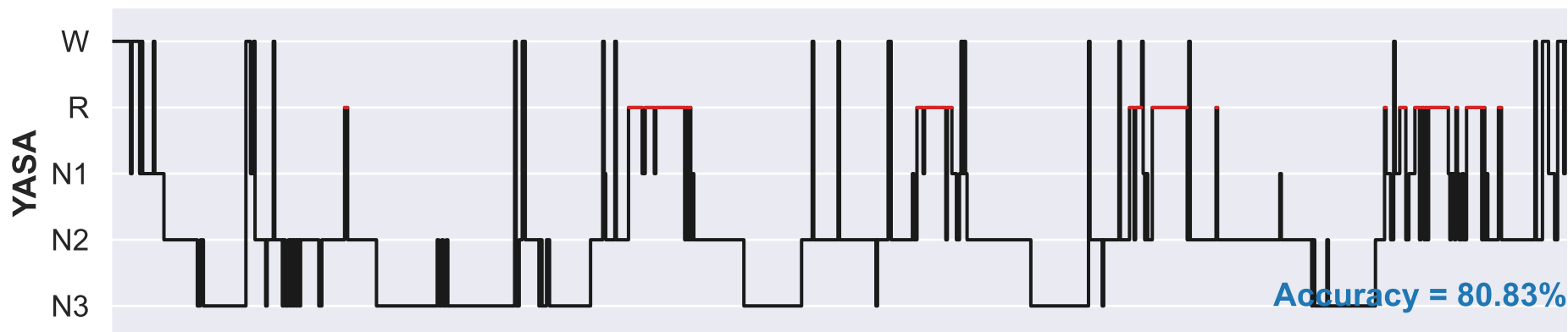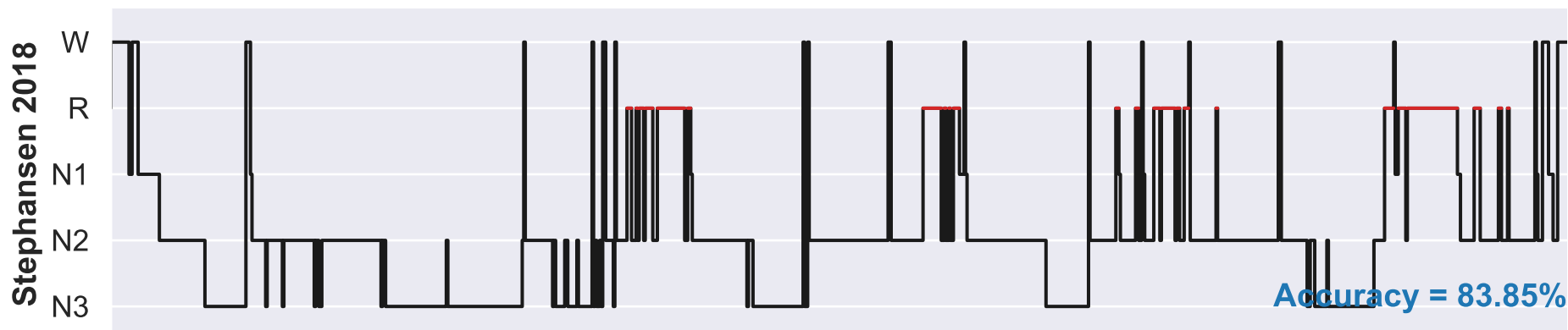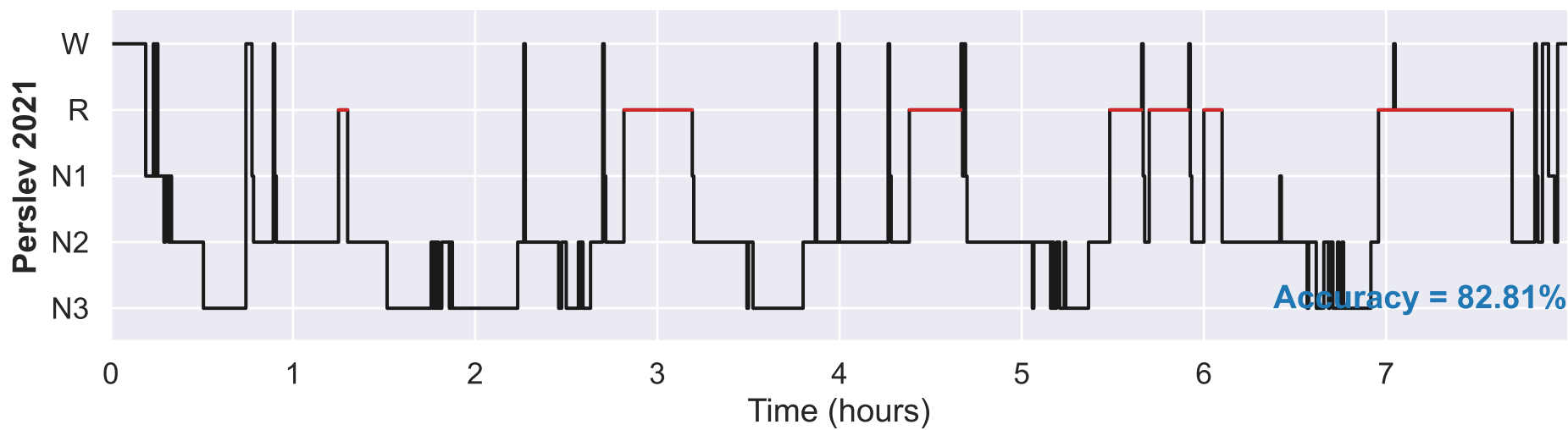

6776c5fc

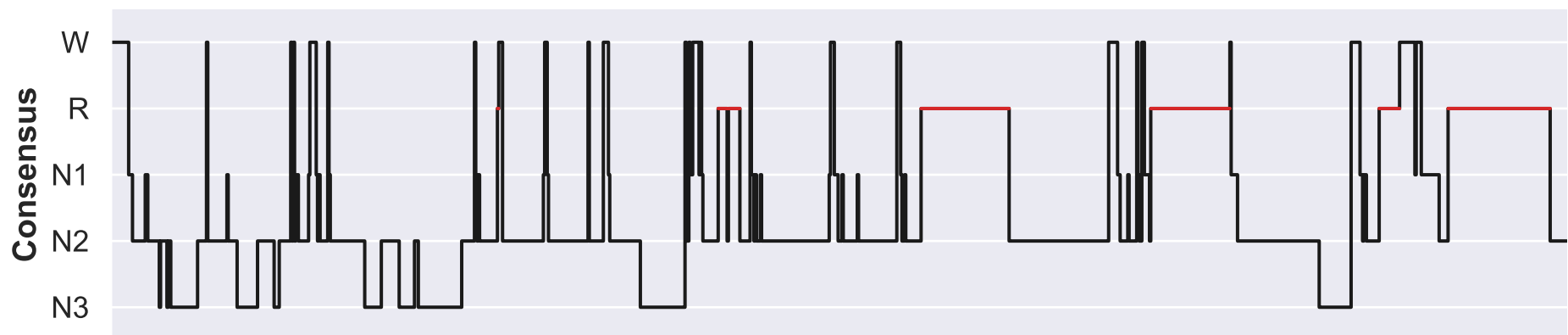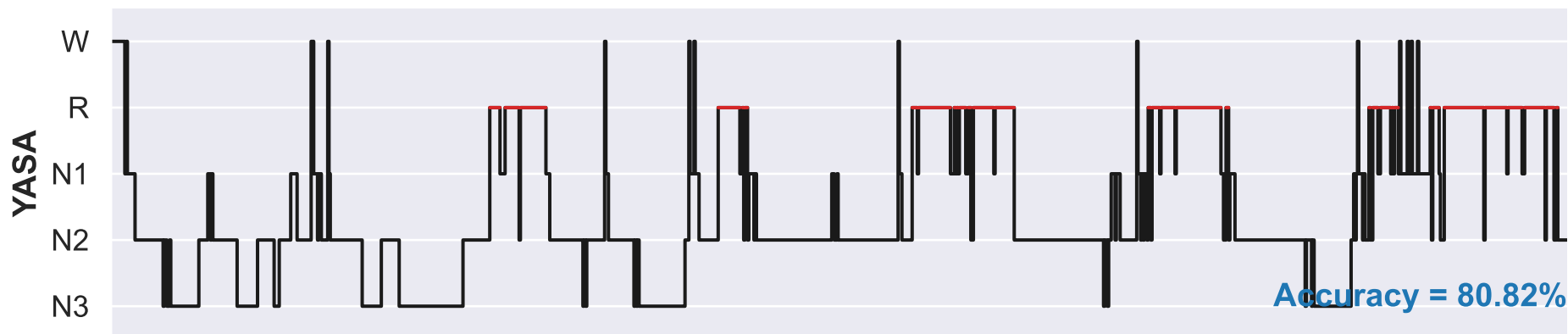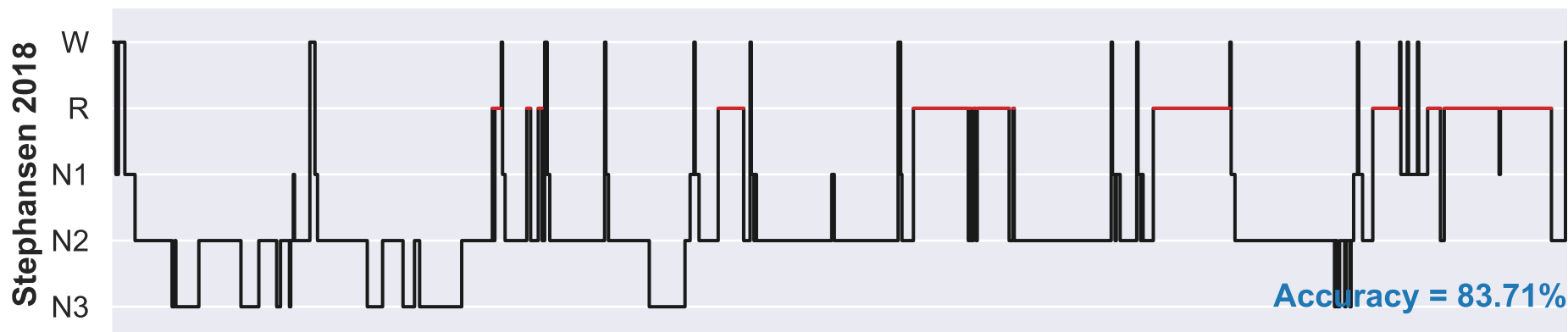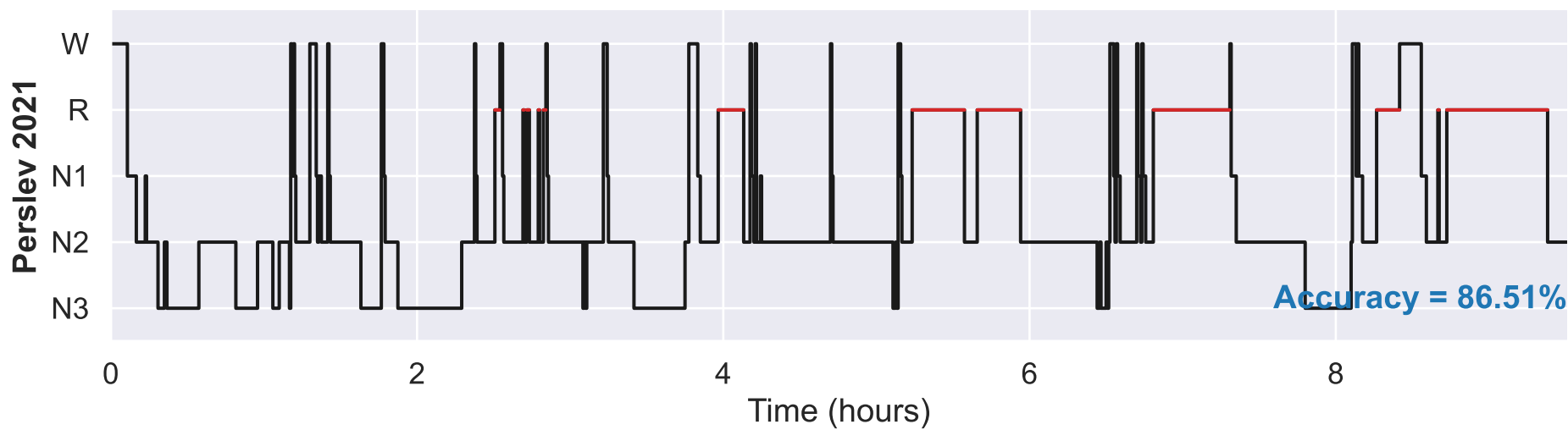

75599827

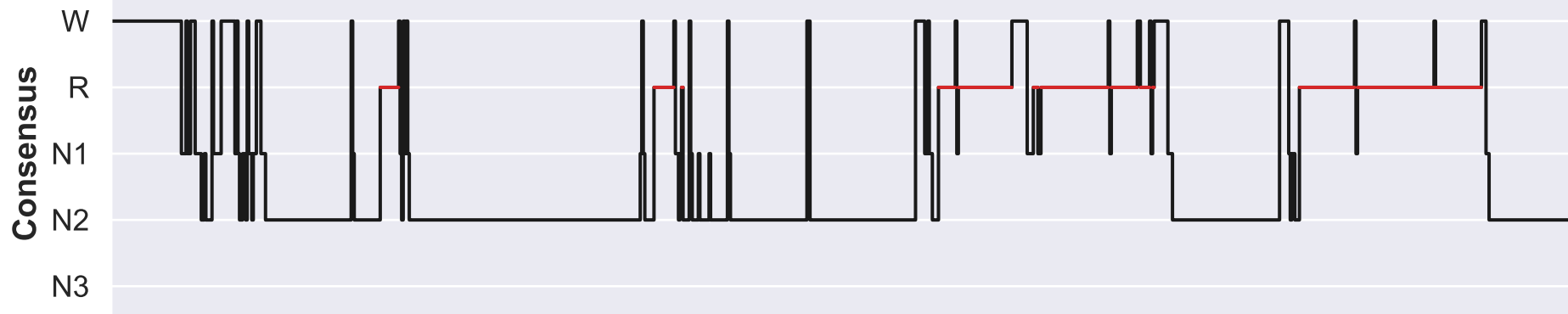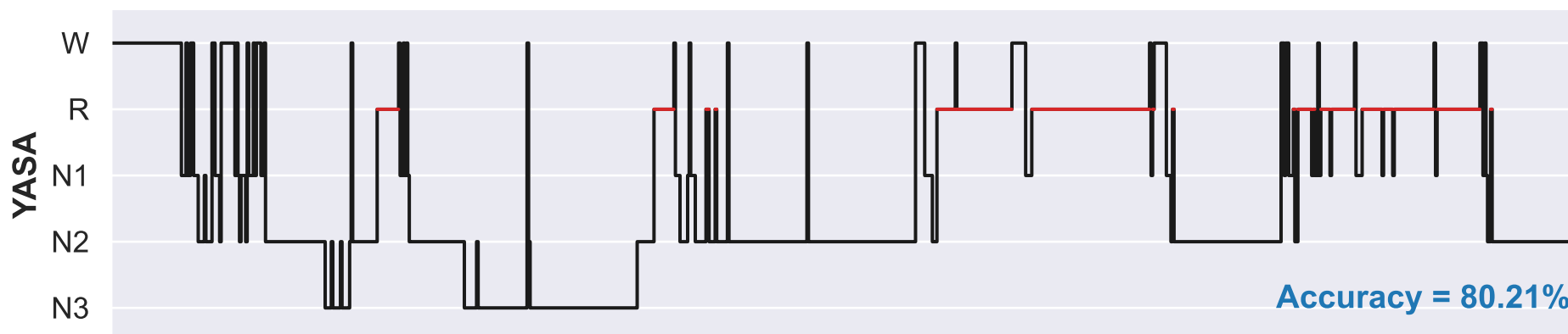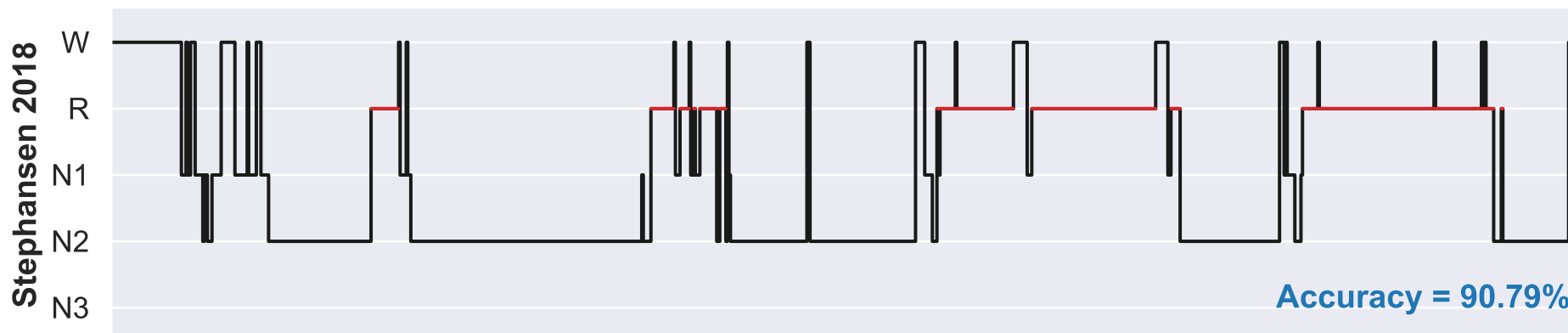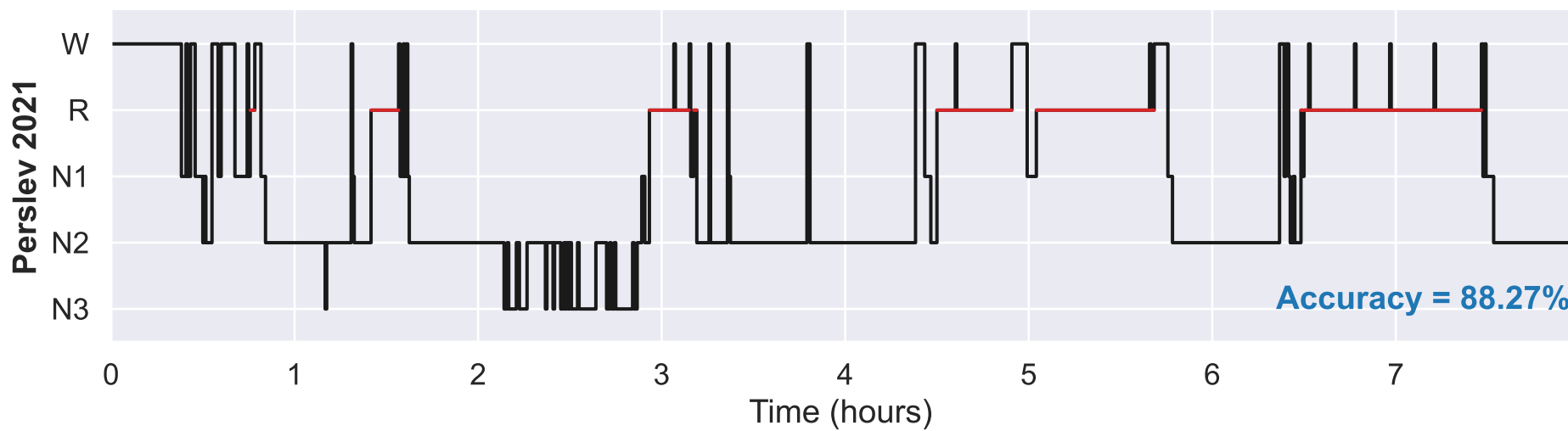

439d4fbf

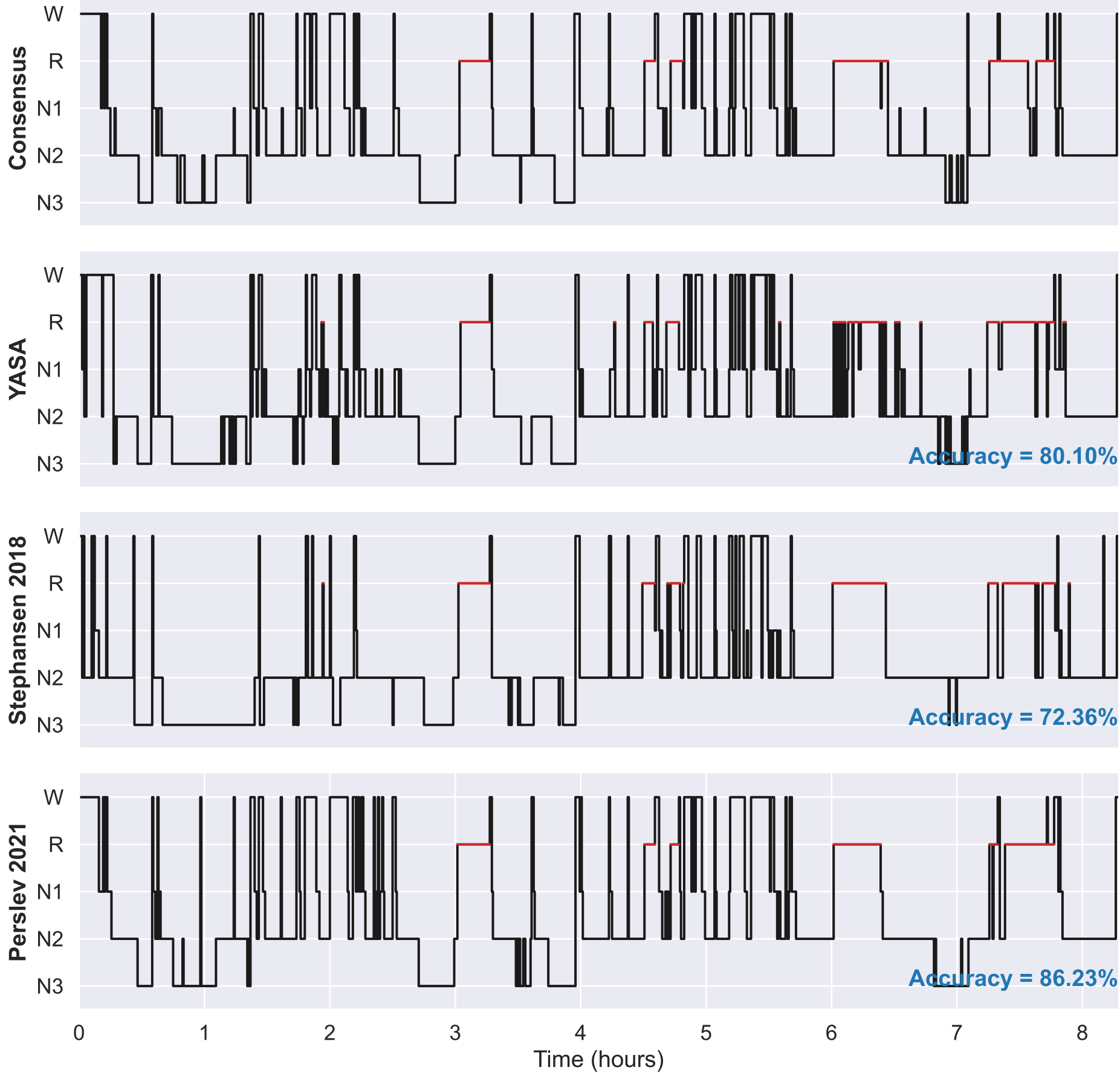

65726d19

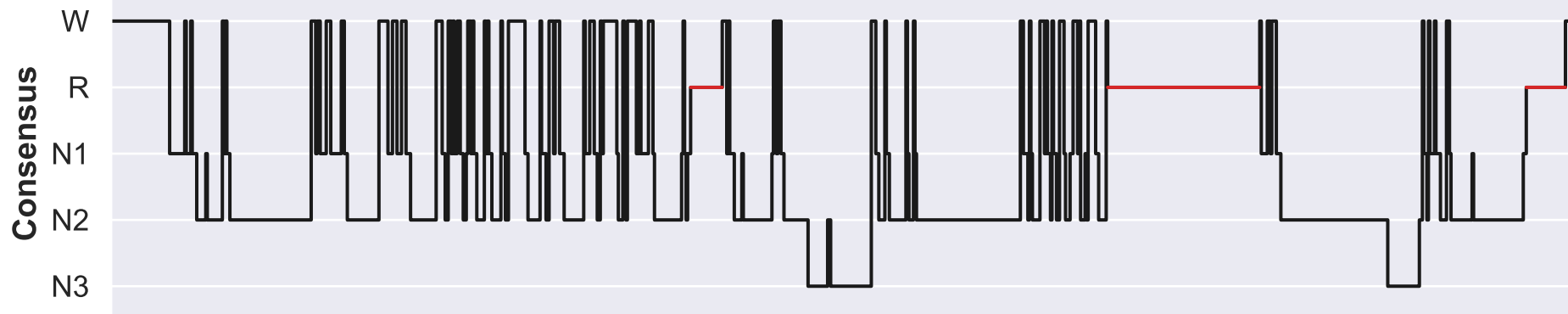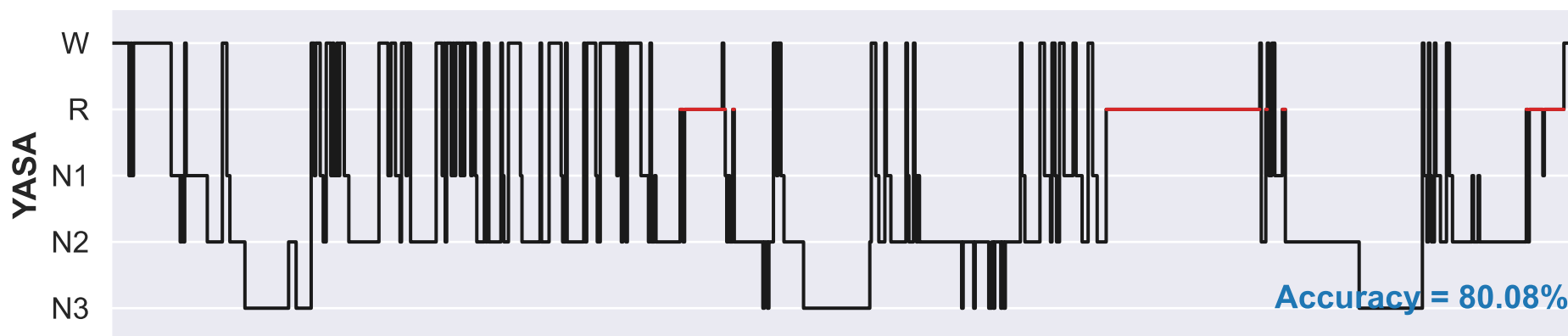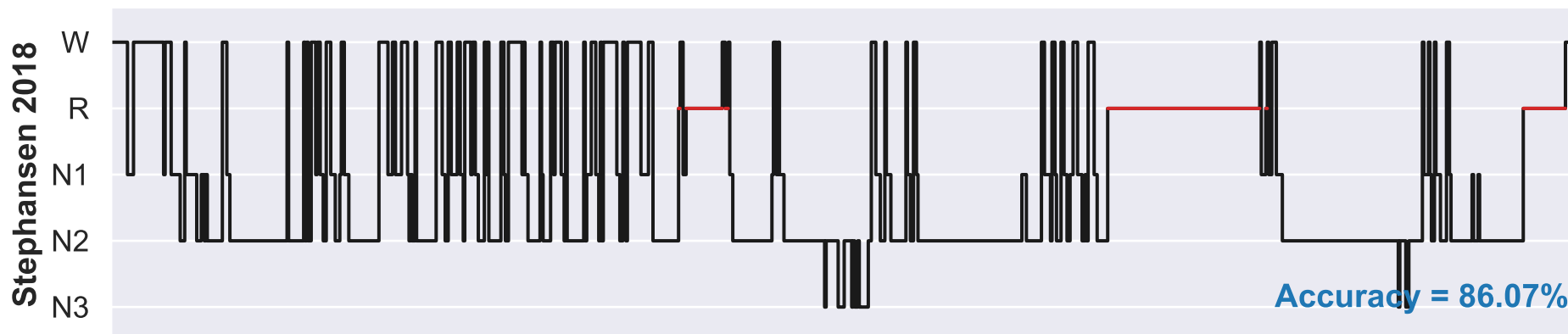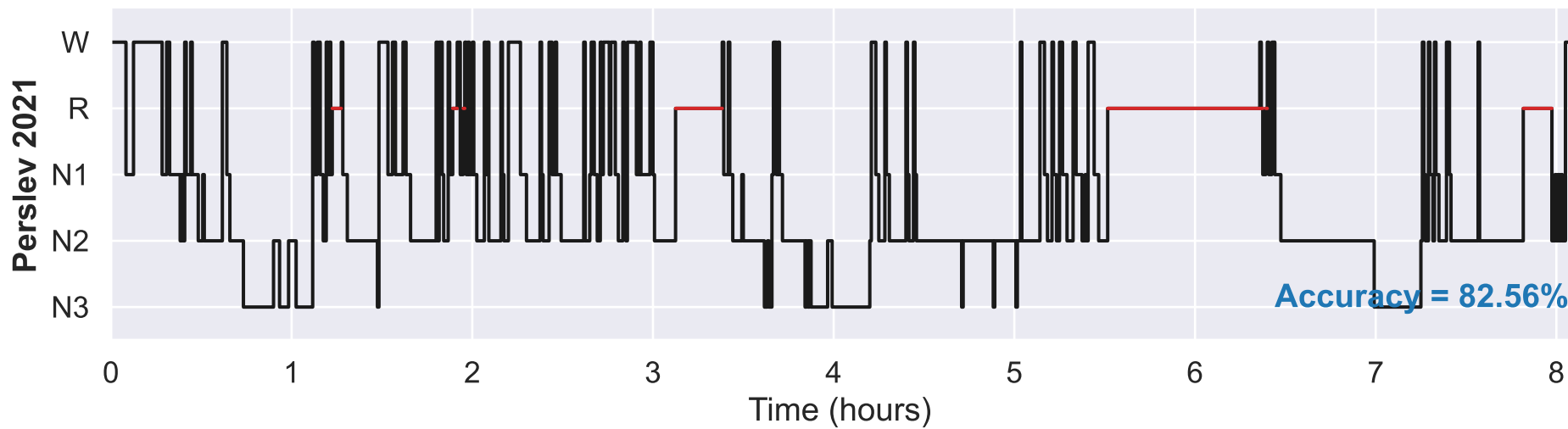

2721aa08

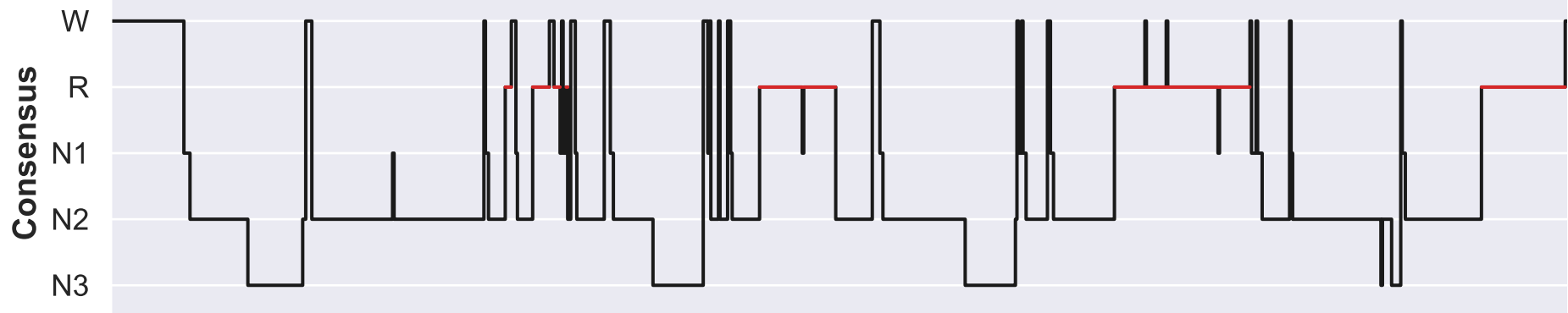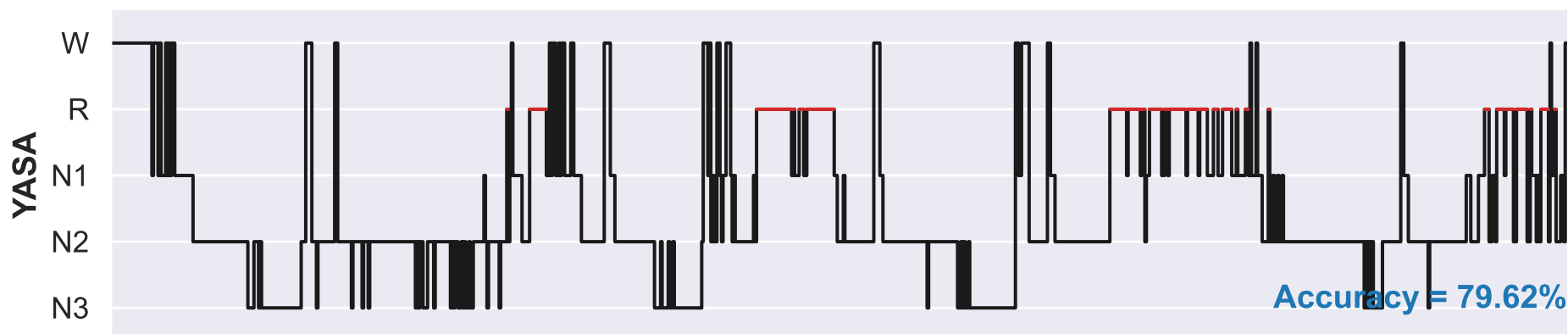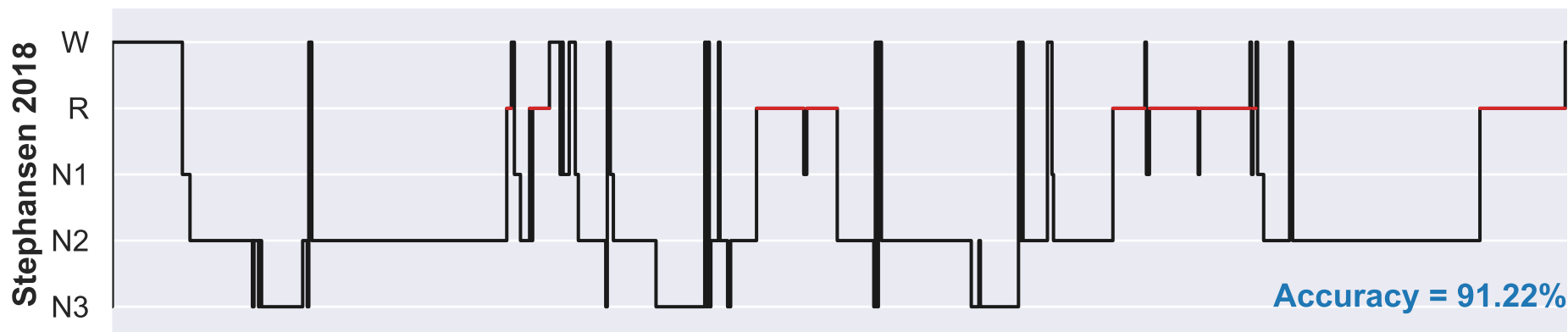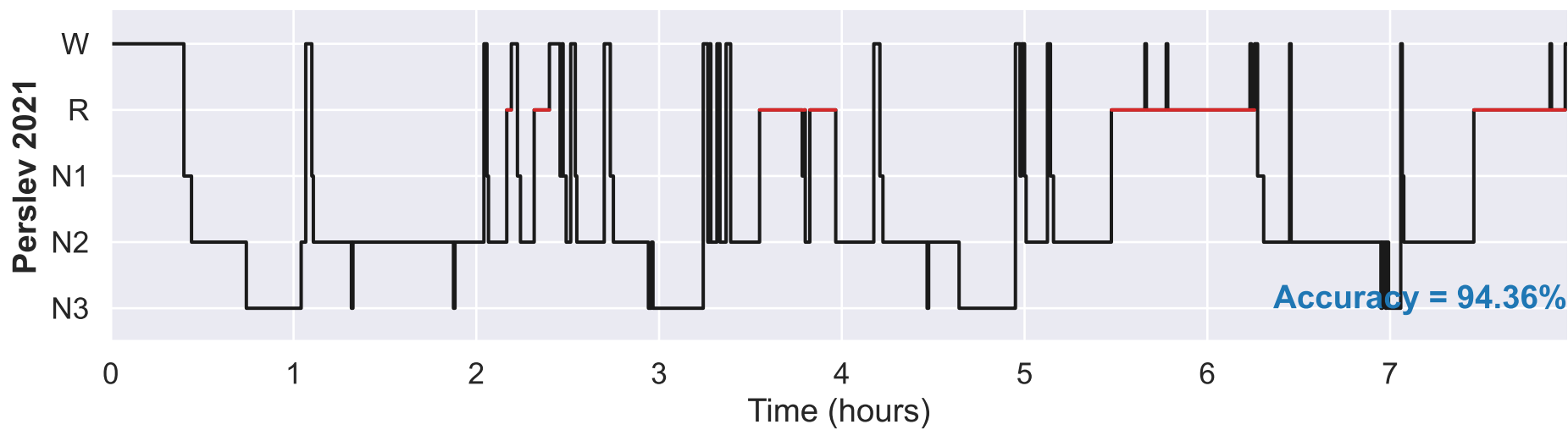

Supplement: Supplementary file 1. — Accuracy refers to the percentage of agreement of the algorithm against the human consensus scoring. Nights are ranked in descending order of agreement between YASA and the consensus scoring. [file elife-70092-supp1.pdf]
