# Supplementary material for: An open-source, high-performance tool for automated sleep staging
Source: eLife. 2021 Oct 14;10:e70092. doi: 10.7554/eLife.70092 (PMC8516415; doi:10.7554/eLife.70092)
Supplement: Supplementary file 2. — Accuracy refers to the percentage of agreement of the algorithm against the human consensus scoring. Nights are ranked in descending order of agreement between YASA and the consensus scoring. [file elife-70092-supp2.pdf]

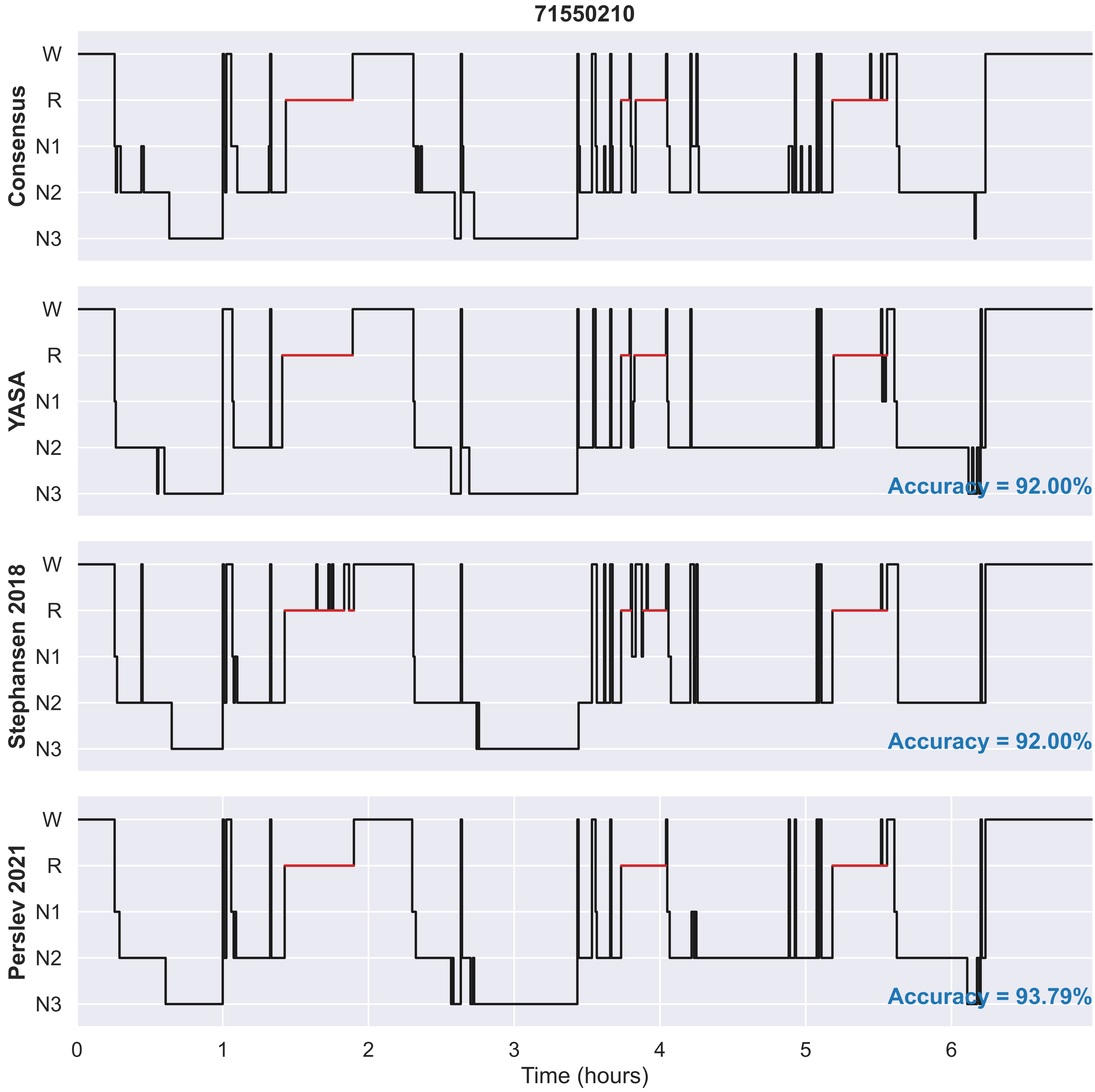
71550210
Consensus
YASA
Stephansen 2018
Perslev 2021
W
R
N1
N2
N3
Accuracy = 92.00%
Accuracy = 92.00%
Accuracy = 93.79%
0
1
2
3
4
5
6
Time (hours)

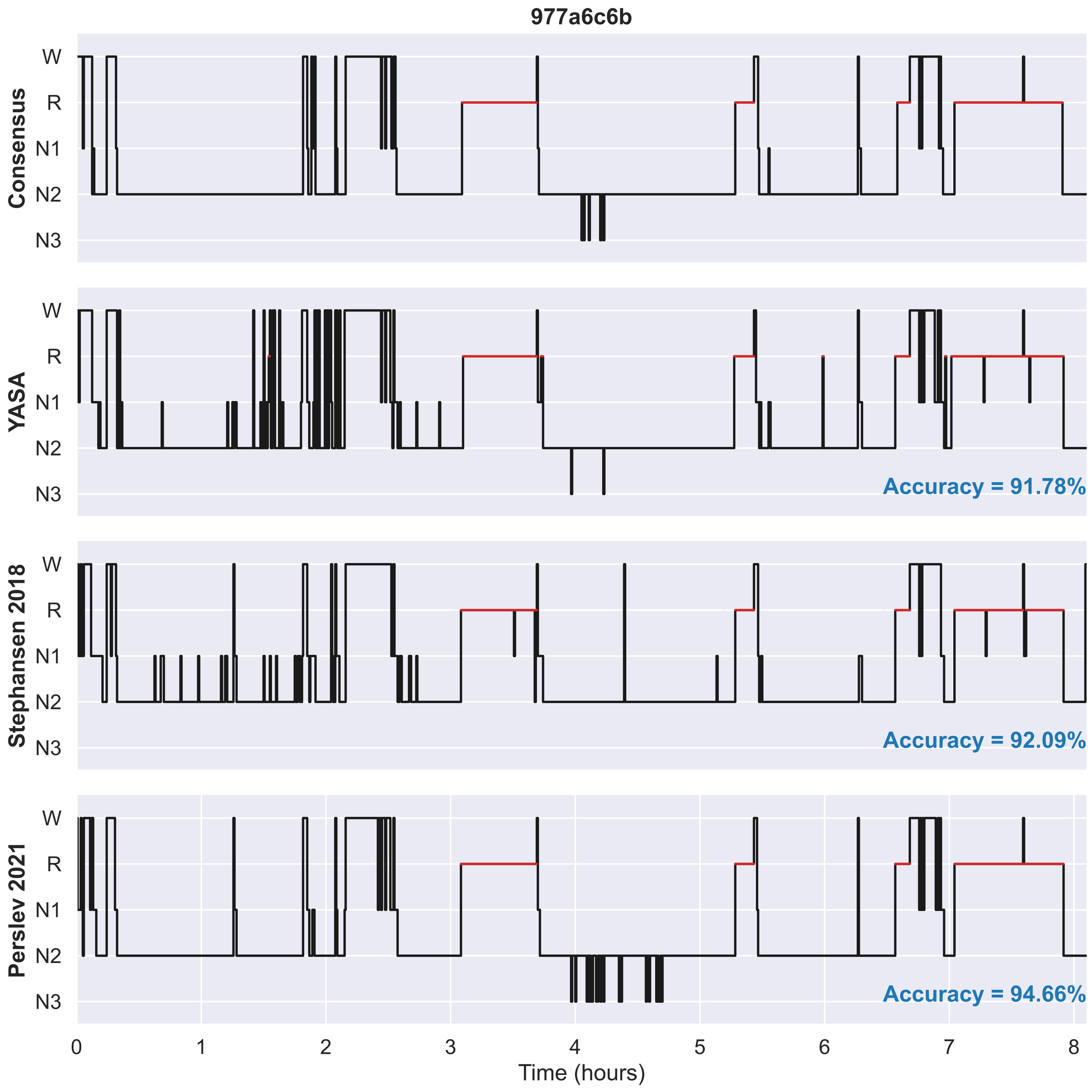

977a6c6b
Consensus
YASA
Stephansen 2018
Perslev 2021
W
R
N1
N2
N3
Accuracy = 91.78%
Accuracy = 92.09%
Accuracy = 94.66%
0
1
2
3
4
5
6
7
8
Time (hours)

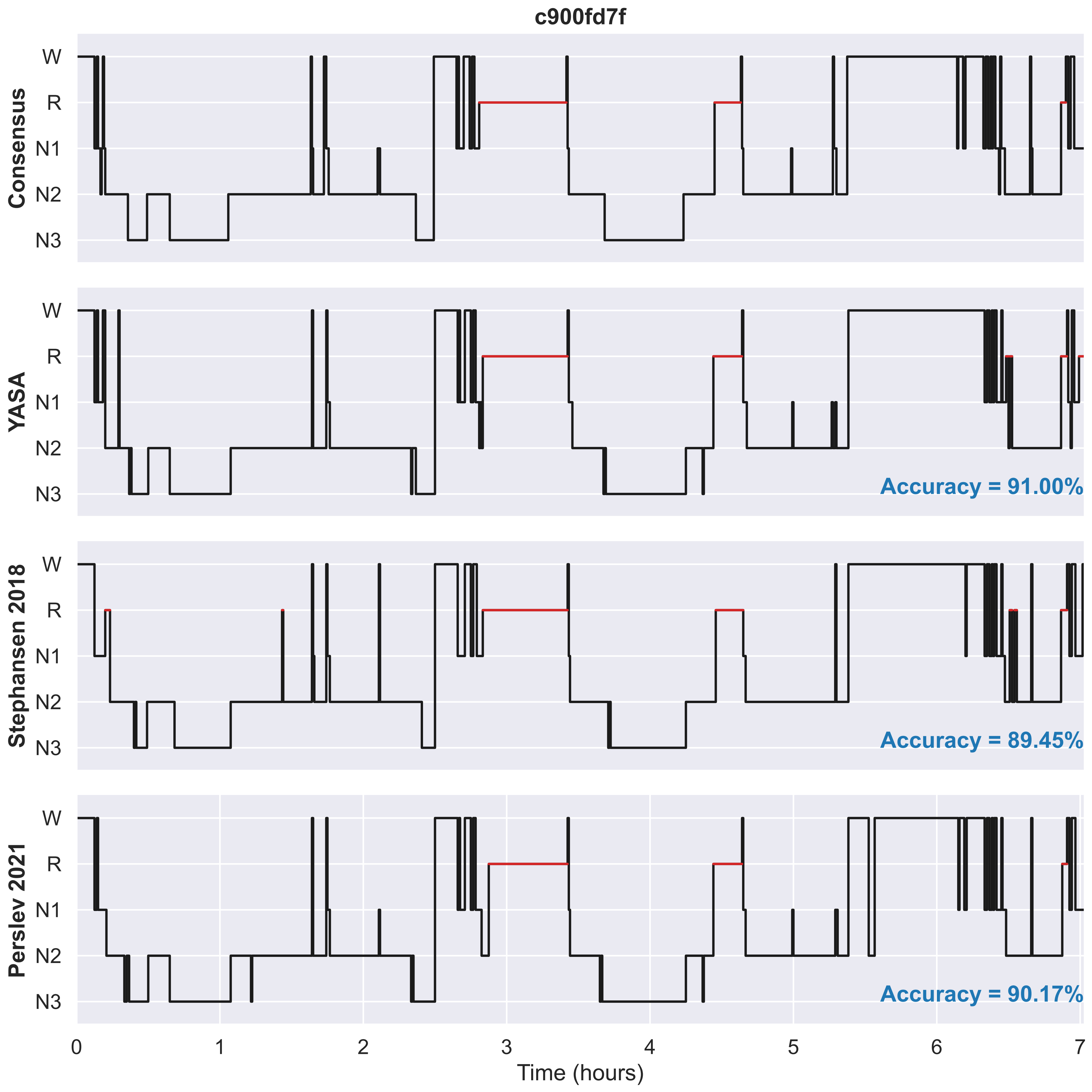

c900fd7f
Consensus
YASA
Stephansen 2018
Perslev 2021
W
R
N1
N2
N3
Accuracy = 91.00%
Accuracy = 89.45%
Accuracy = 90.17%
0
1
2
3
4
5
6
7
Time (hours)

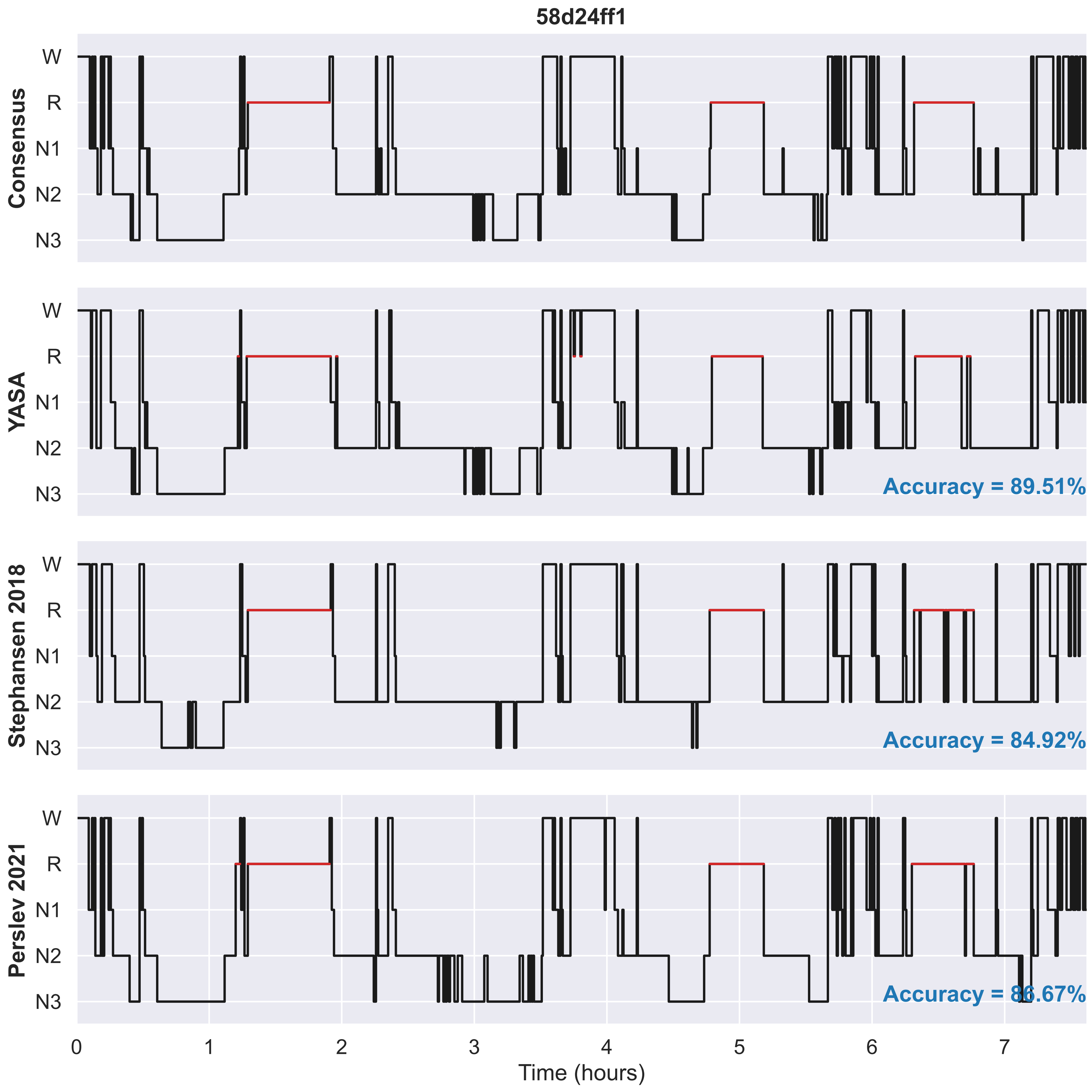

58d24ff1
Consensus
YASA
Stephansen 2018
Perslev 2021
W
R
N1
N2
N3
Accuracy = 89.51%
Accuracy = 84.92%
Accuracy = 86.67%
0
1
2
3
4
5
6
7
Time (hours)

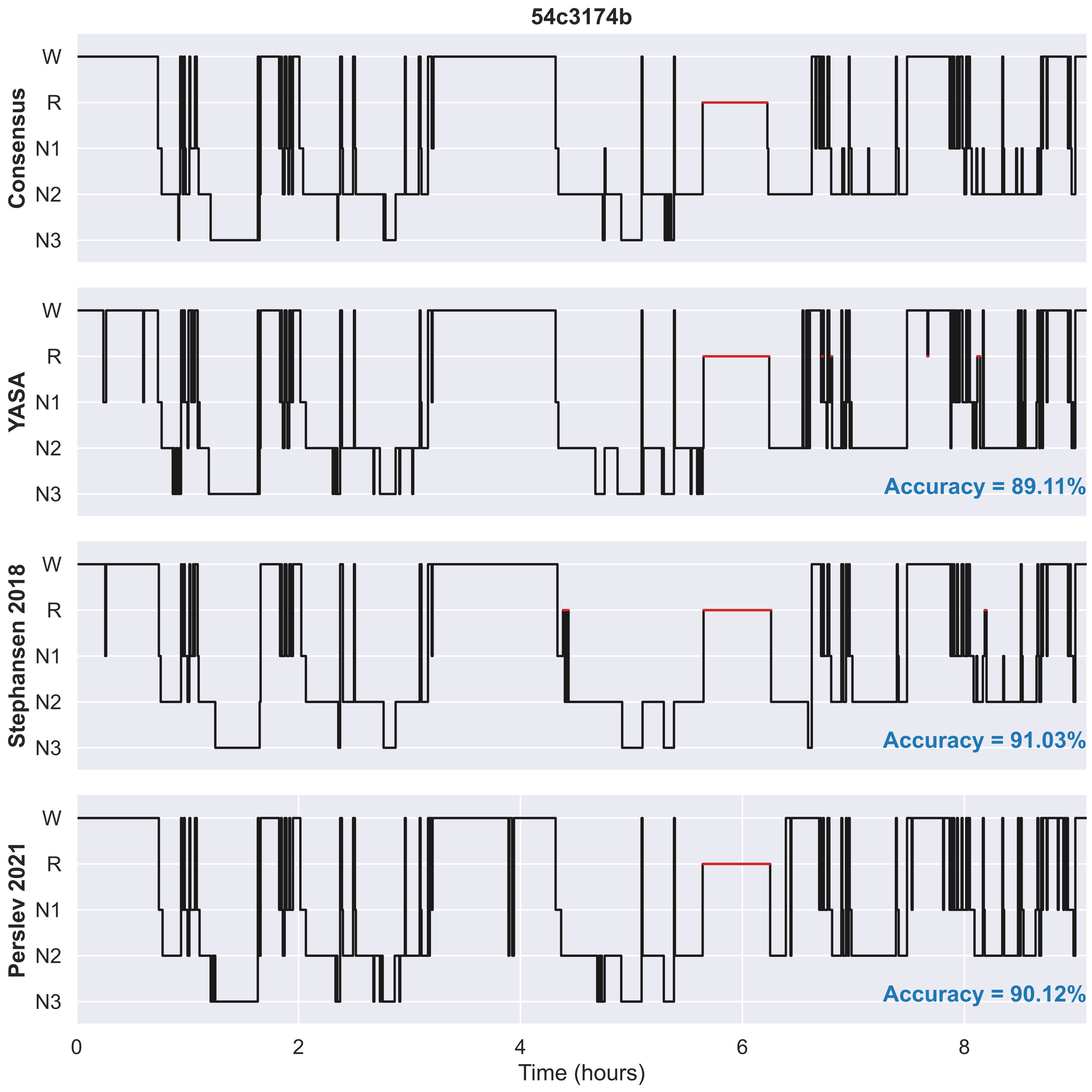

54c3174b
Consensus
YASA
Stephansen 2018
Perslev 2021
W
R
N1
N2
N3
Accuracy = 89.11%
Accuracy = 91.03%
Accuracy = 90.12%
0
2
4
6
8
Time (hours)

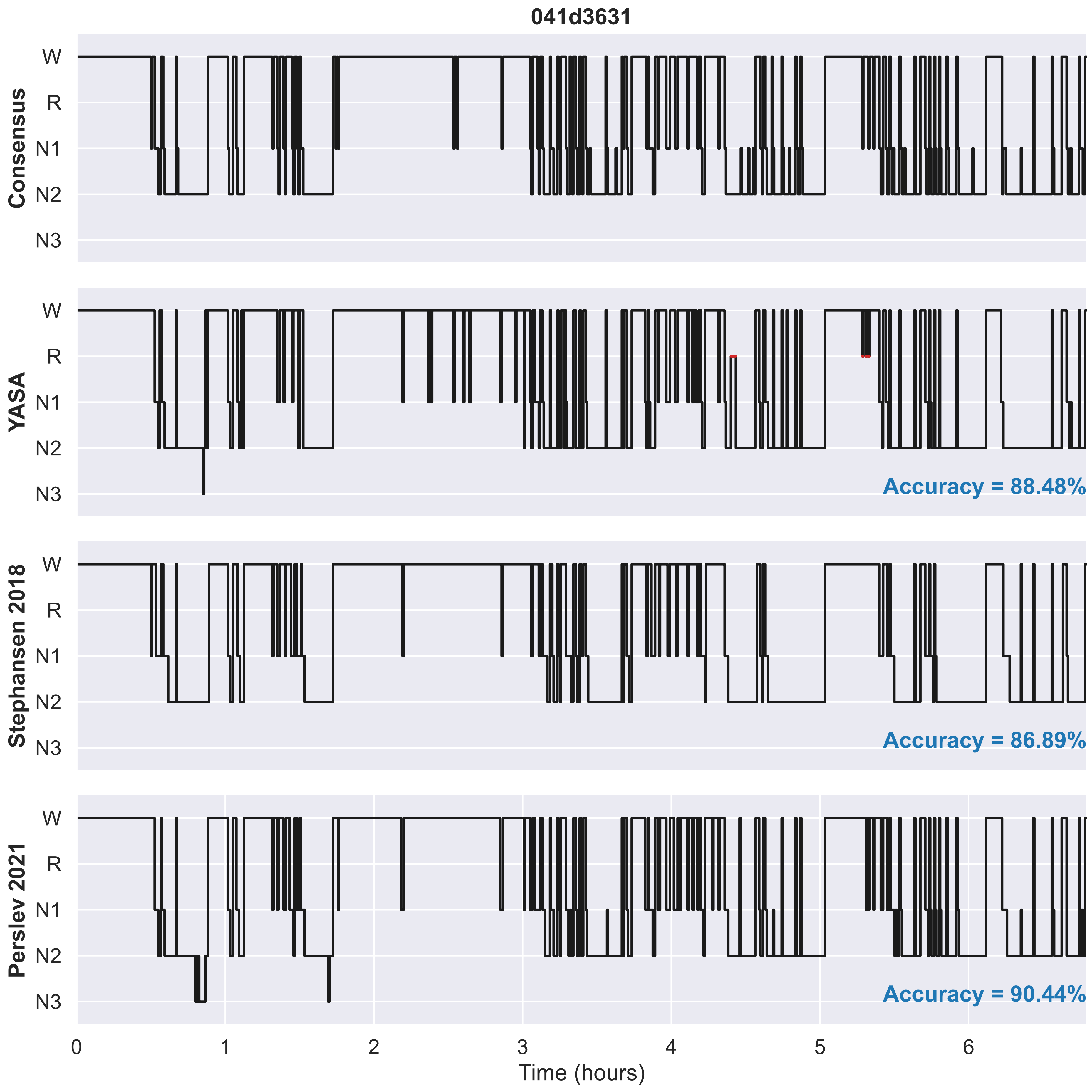

041d3631
Consensus
YASA
Stephansen 2018
Perslev 2021
W
R
N1
N2
N3
Accuracy = 88.48%
Accuracy = 86.89%
Accuracy = 90.44%
0
1
2
3
4
5
6
Time (hours)

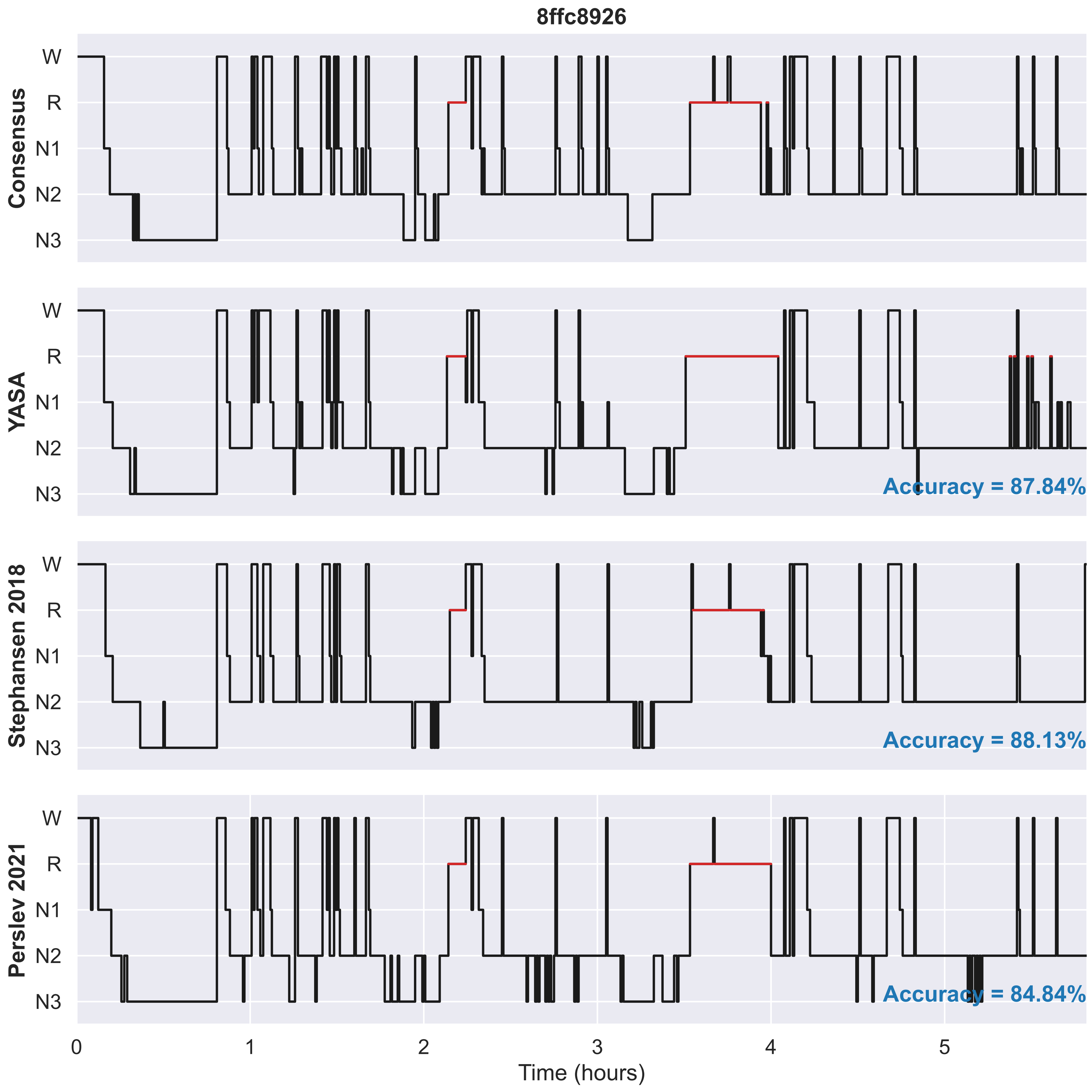

8ffc8926
Consensus
YASA
Stephansen 2018
Perslev 2021
W
R
N1
N2
N3
Accuracy = 87.84%
Accuracy = 88.13%
Accuracy = 84.84%
0
1
2
3
4
5
Time (hours)

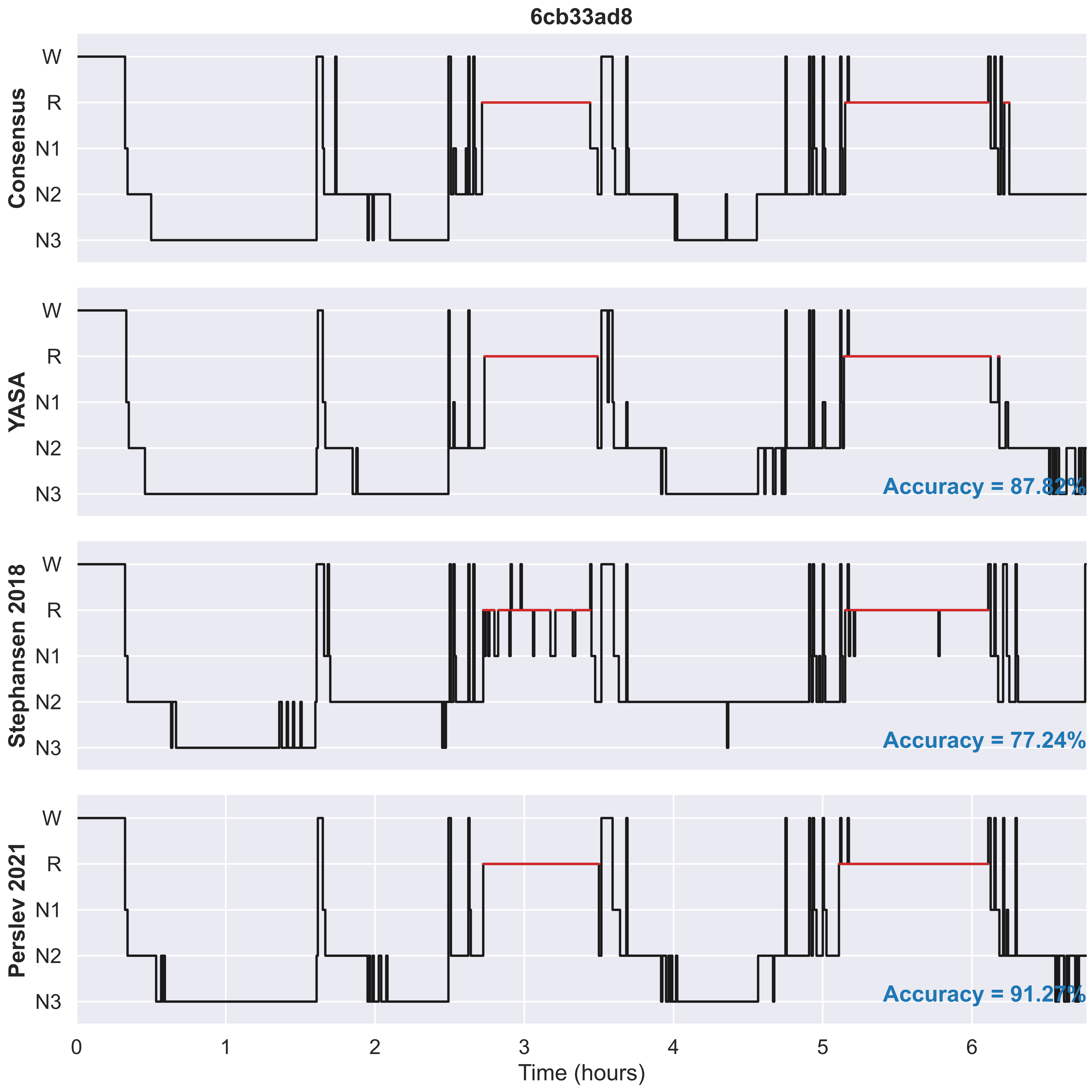

6cb33ad8
Consensus
YASA
Accuracy = 87.82%
Stephansen 2018
Accuracy = 77.24%
Perslev 2021
Accuracy = 91.27%
W
R
N1
N2
N3
0
1
2
3
4
5
6
Time (hours)

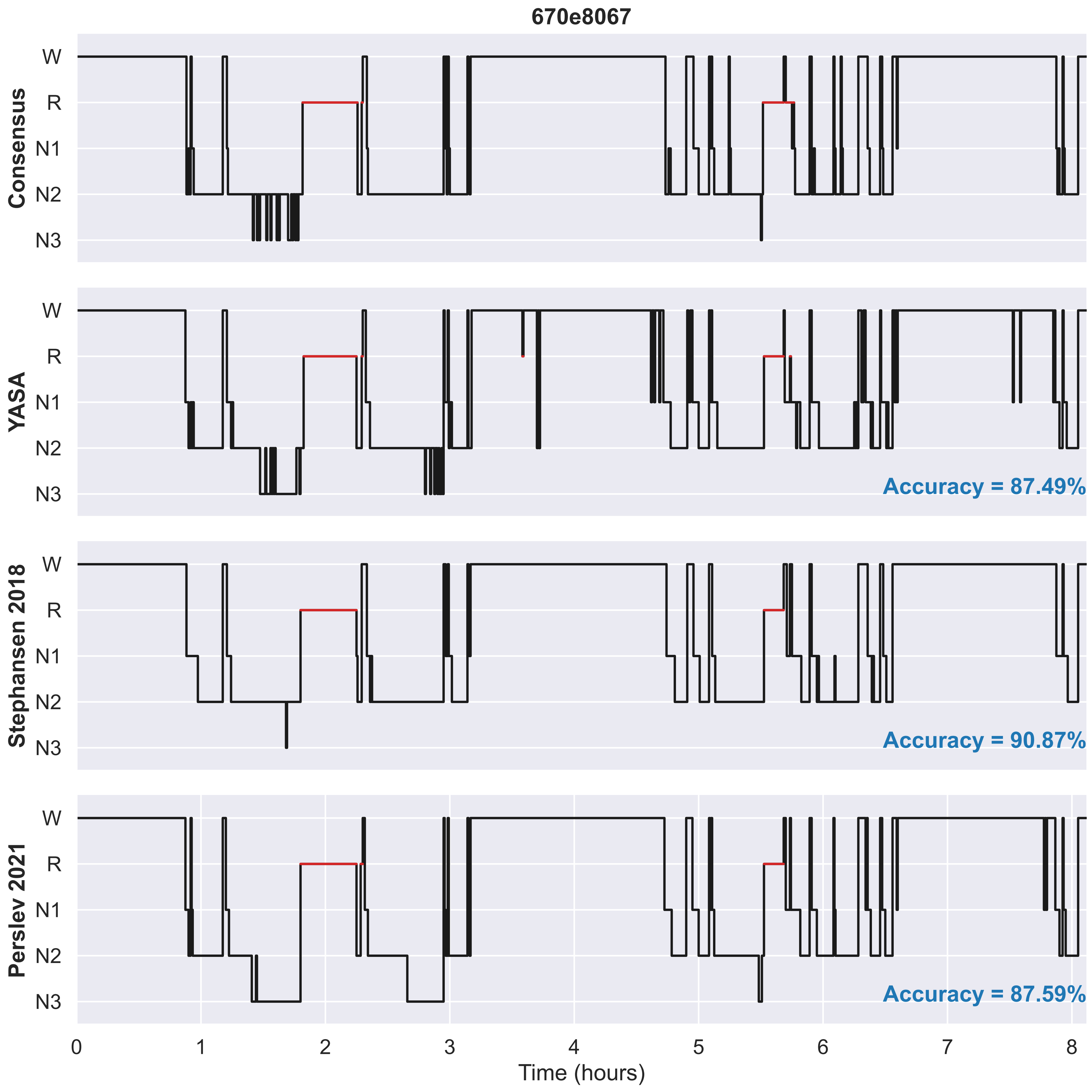
670e8067
Consensus
YASA
Stephansen 2018
Perslev 2021
W
R
N1
N2
N3
Accuracy = 87.49%
Accuracy = 90.87%
Accuracy = 87.59%
0
1
2
3
4
5
6
7
8
Time (hours)

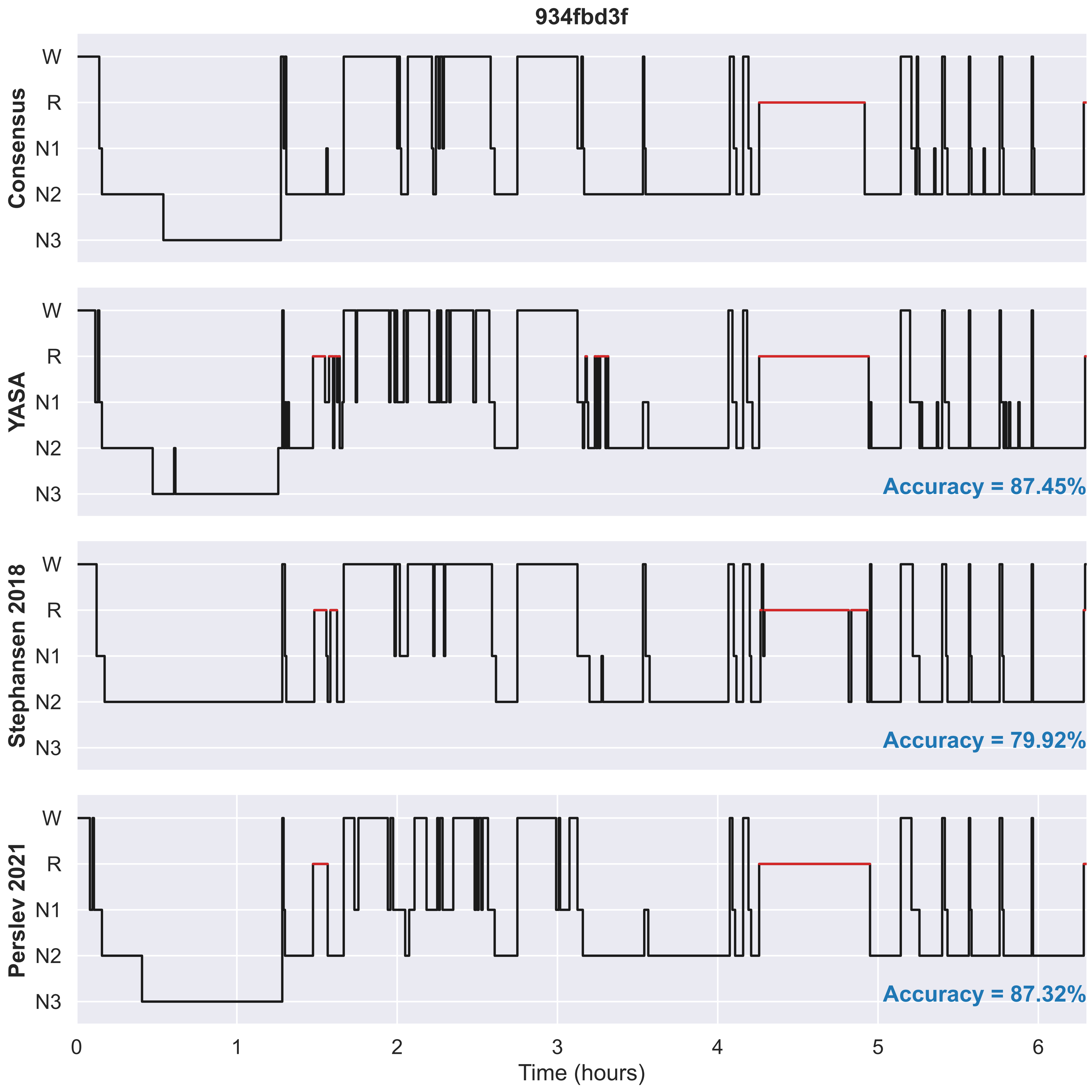
934fbd3f
Consensus
YASA
Stephansen 2018
Perslev 2021
W
R
N1
N2
N3
Accuracy = 87.45%
Accuracy = 79.92%
Accuracy = 87.32%
0
1
2
3
4
5
6
Time (hours)

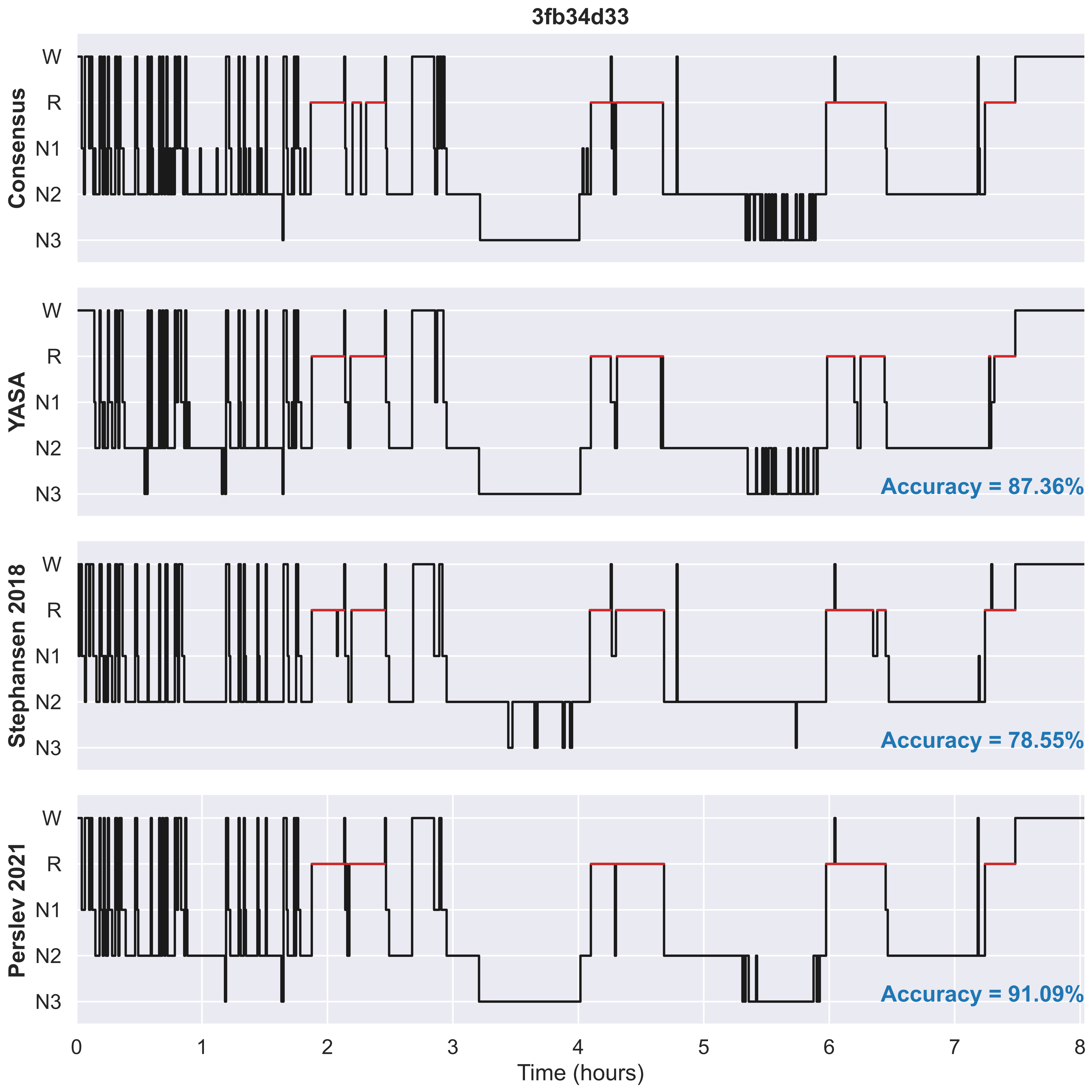

3fb34d33
Consensus
YASA
Stephansen 2018
Perslev 2021
W
R
N1
N2
N3
Accuracy = 87.36%
Accuracy = 78.55%
Accuracy = 91.09%
0
1
2
3
4
5
6
7
8
Time (hours)

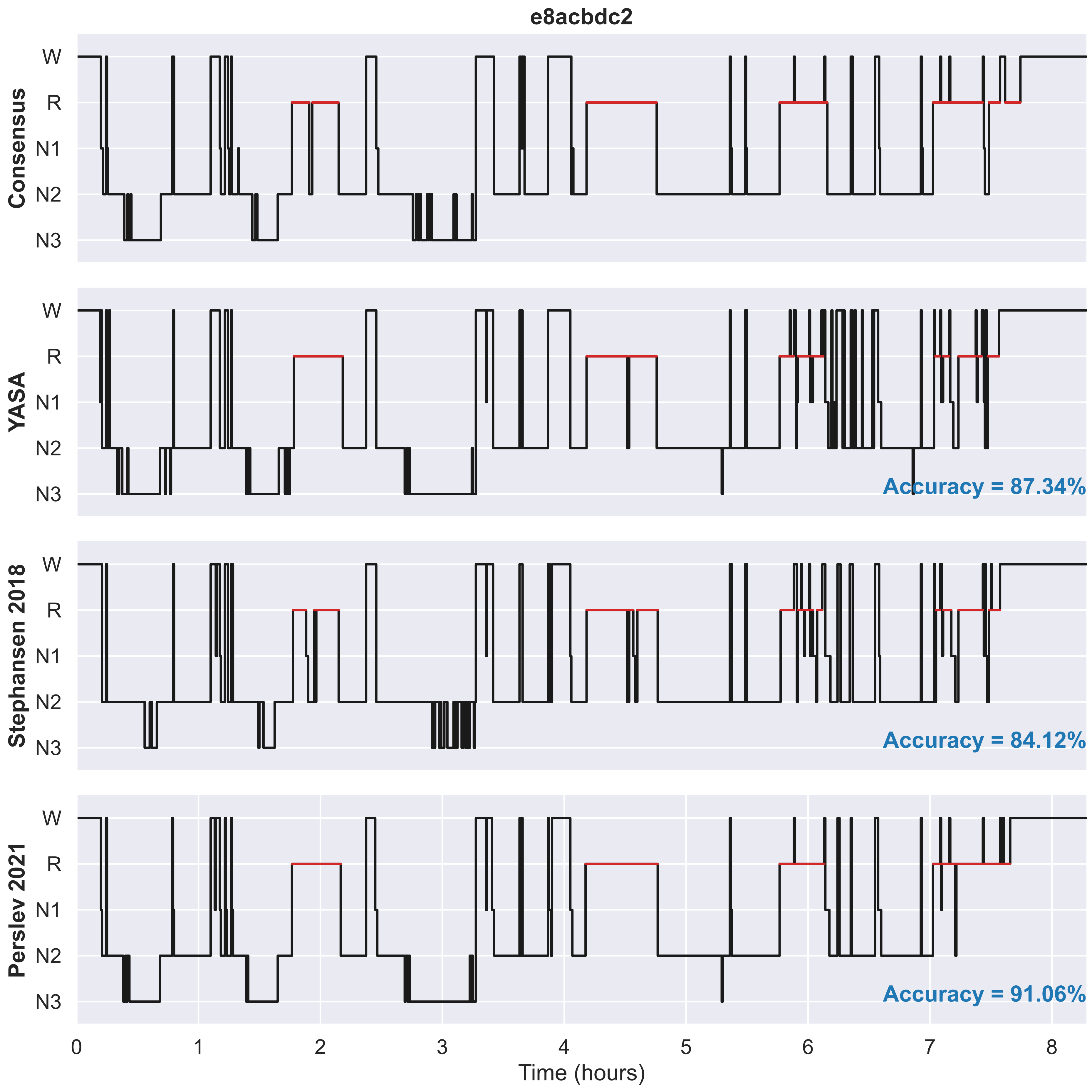

e8acbdc2
Consensus
YASA
Stephansen 2018
Perslev 2021
W
R
N1
N2
N3
Accuracy = 87.34%
Accuracy = 84.12%
Accuracy = 91.06%
0
1
2
3
4
5
6
7
8
Time (hours)

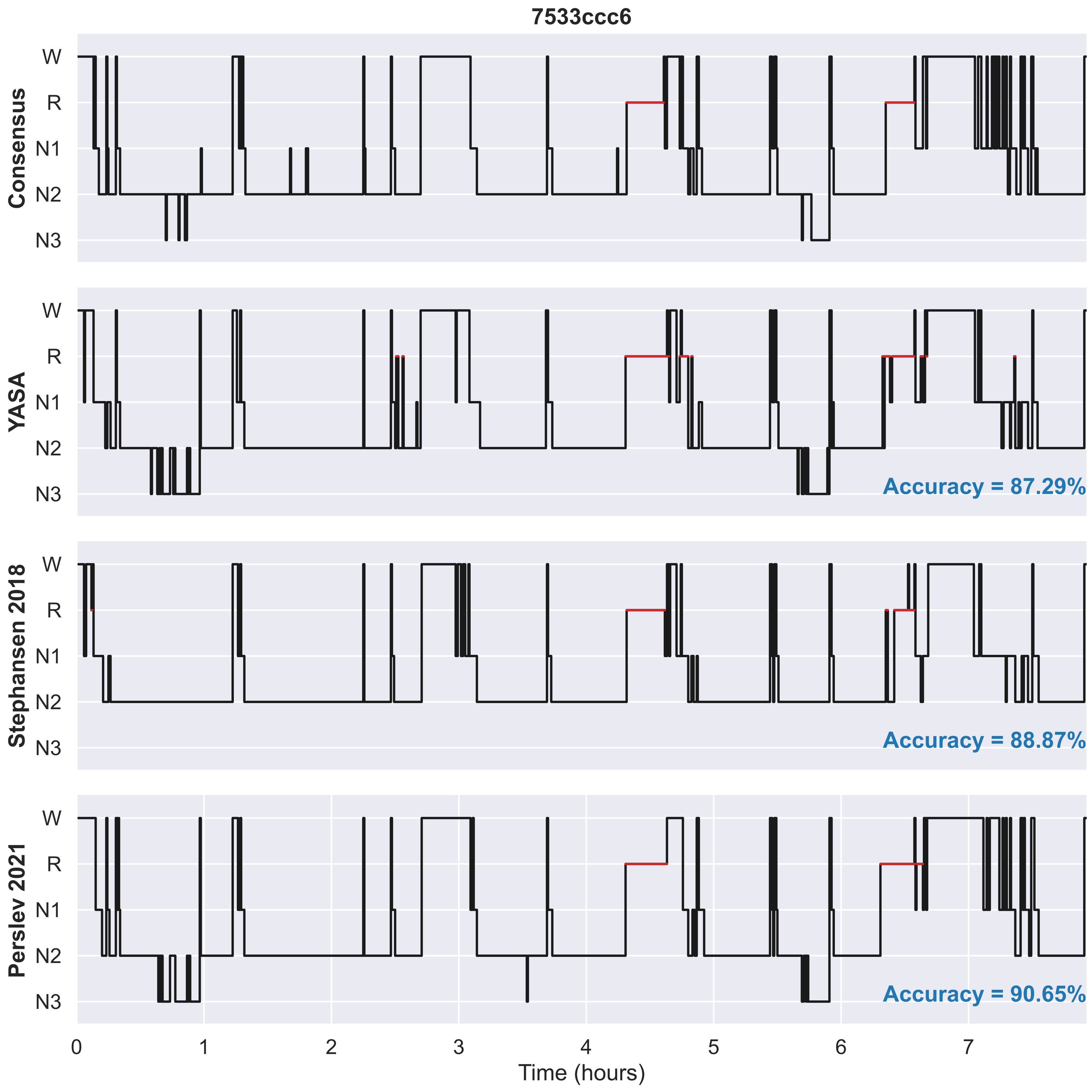

7533ccc6
Consensus
YASA
Stephansen 2018
Perslev 2021
W
R
N1
N2
N3
Accuracy = 87.29%
Accuracy = 88.87%
Accuracy = 90.65%
0
1
2
3
4
5
6
7
Time (hours)

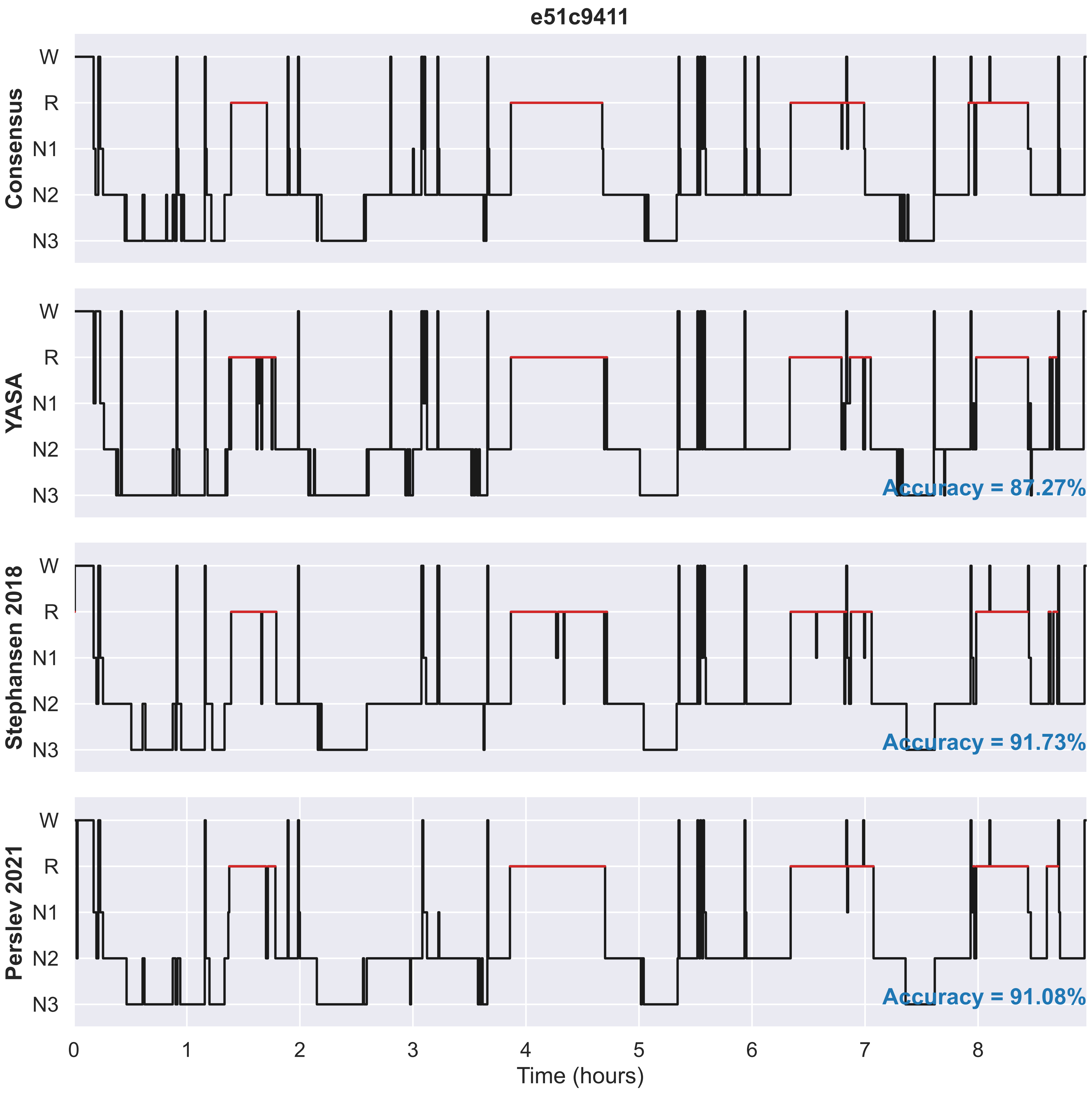
e51c9411
Consensus
YASA
Stephansen 2018
Perslev 2021
W
R
N1
N2
N3
Accuracy = 87.27%
Accuracy = 91.73%
Accuracy = 91.08%
0
1
2
3
4
5
6
7
8
Time (hours)

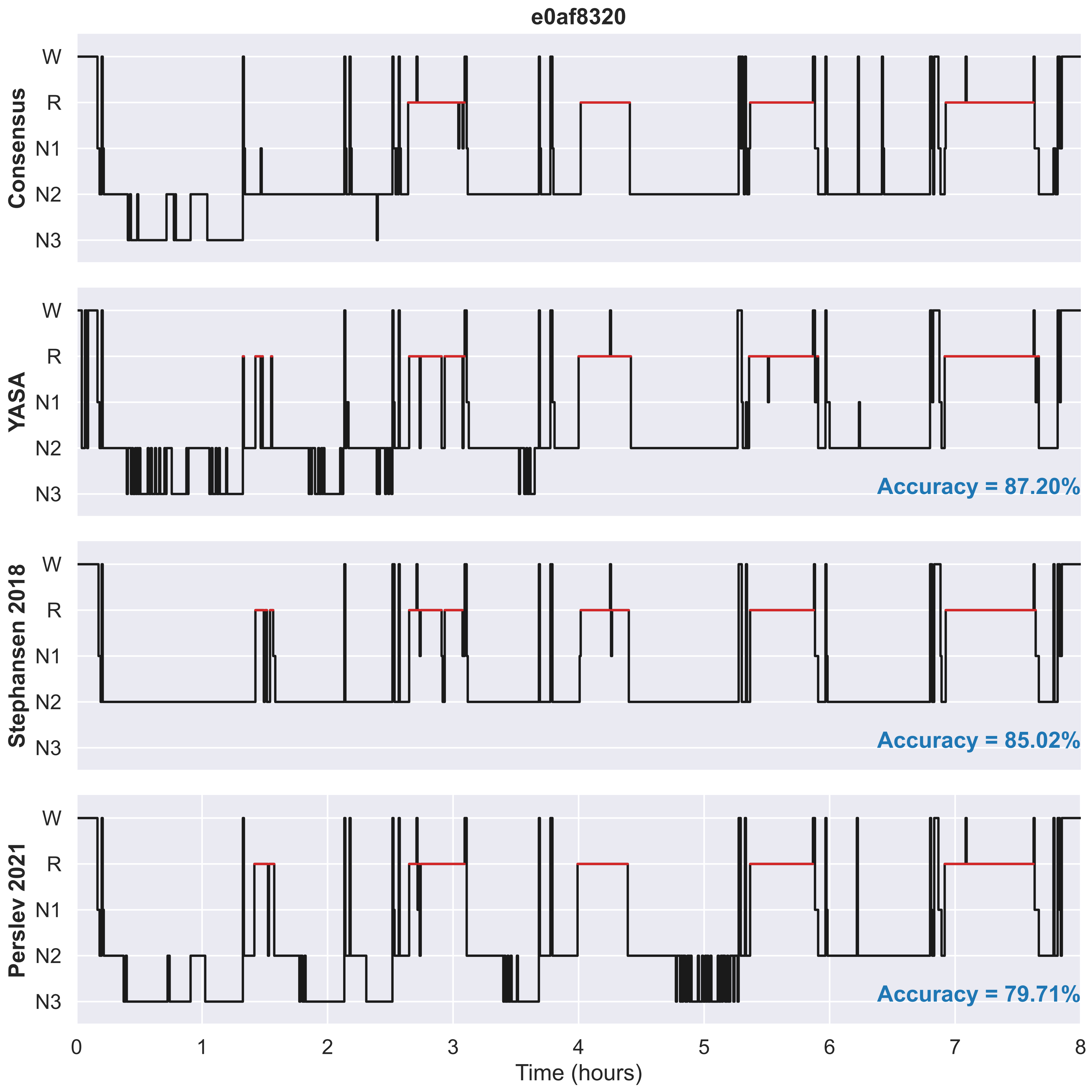
e0af8320
Consensus
YASA
Stephansen 2018
Perslev 2021
W
R
N1
N2
N3
Accuracy = 87.20%
Accuracy = 85.02%
Accuracy = 79.71%
0
1
2
3
4
5
6
7
8
Time (hours)

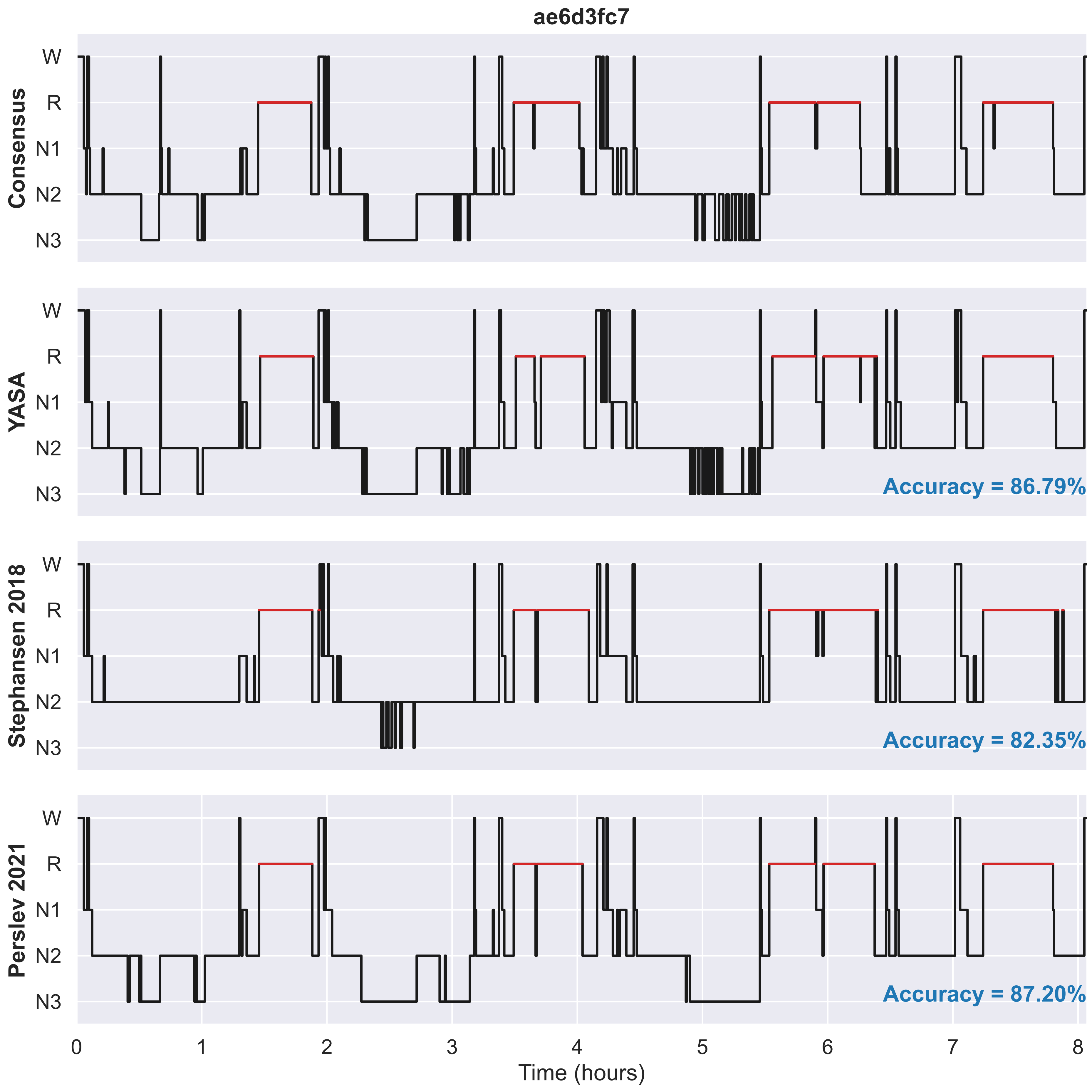
ae6d3fc7
Consensus
YASA
Stephansen 2018
Perslev 2021
W
R
N1
N2
N3
Accuracy = 86.79%
Accuracy = 82.35%
Accuracy = 87.20%
0
1
2
3
4
5
6
7
8
Time (hours)

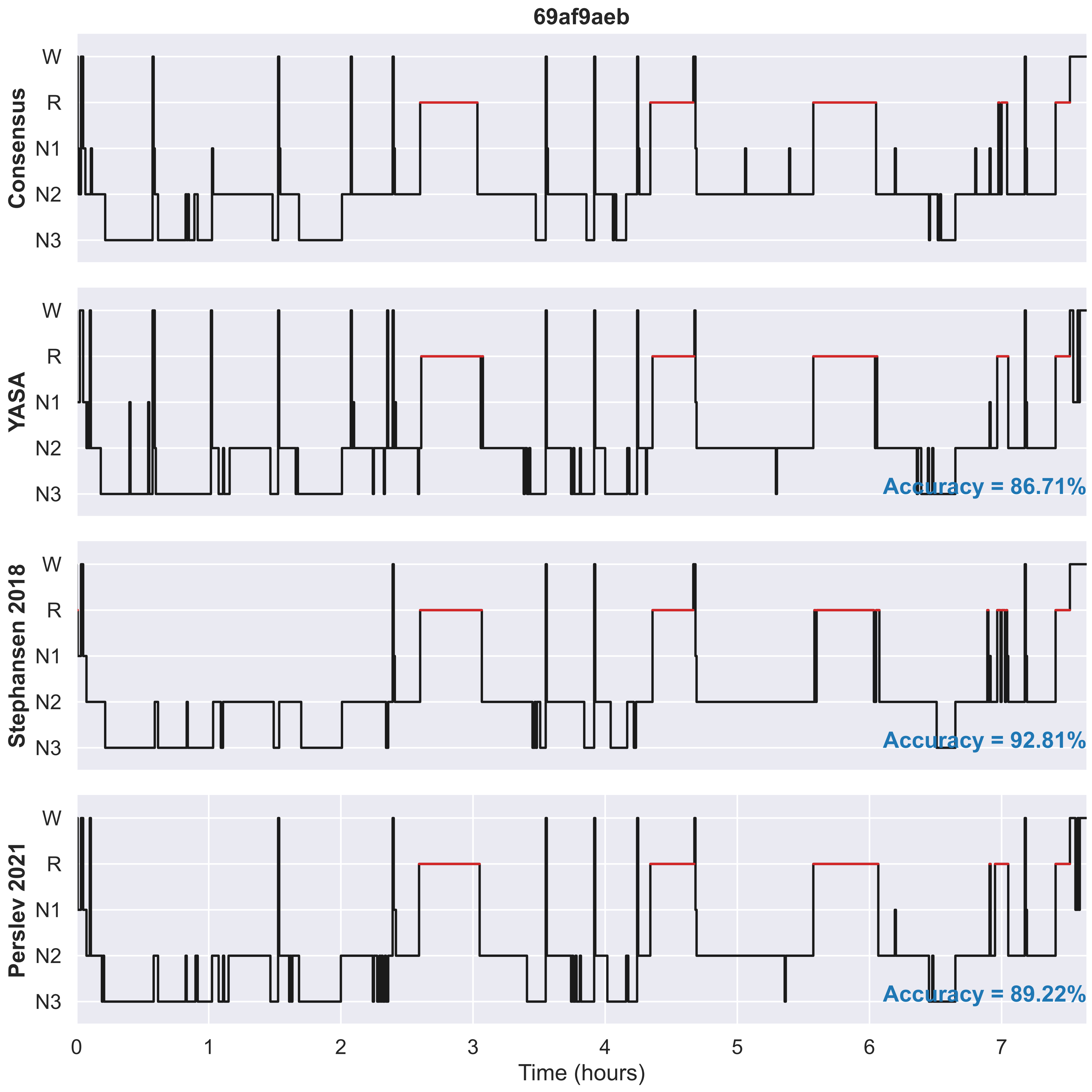
69af9aeb
Consensus
YASA
Stephansen 2018
Perslev 2021
W
R
N1
N2
N3
Accuracy = 86.71%
Accuracy = 92.81%
Accuracy = 89.22%
0
1
2
3
4
5
6
7
Time (hours)

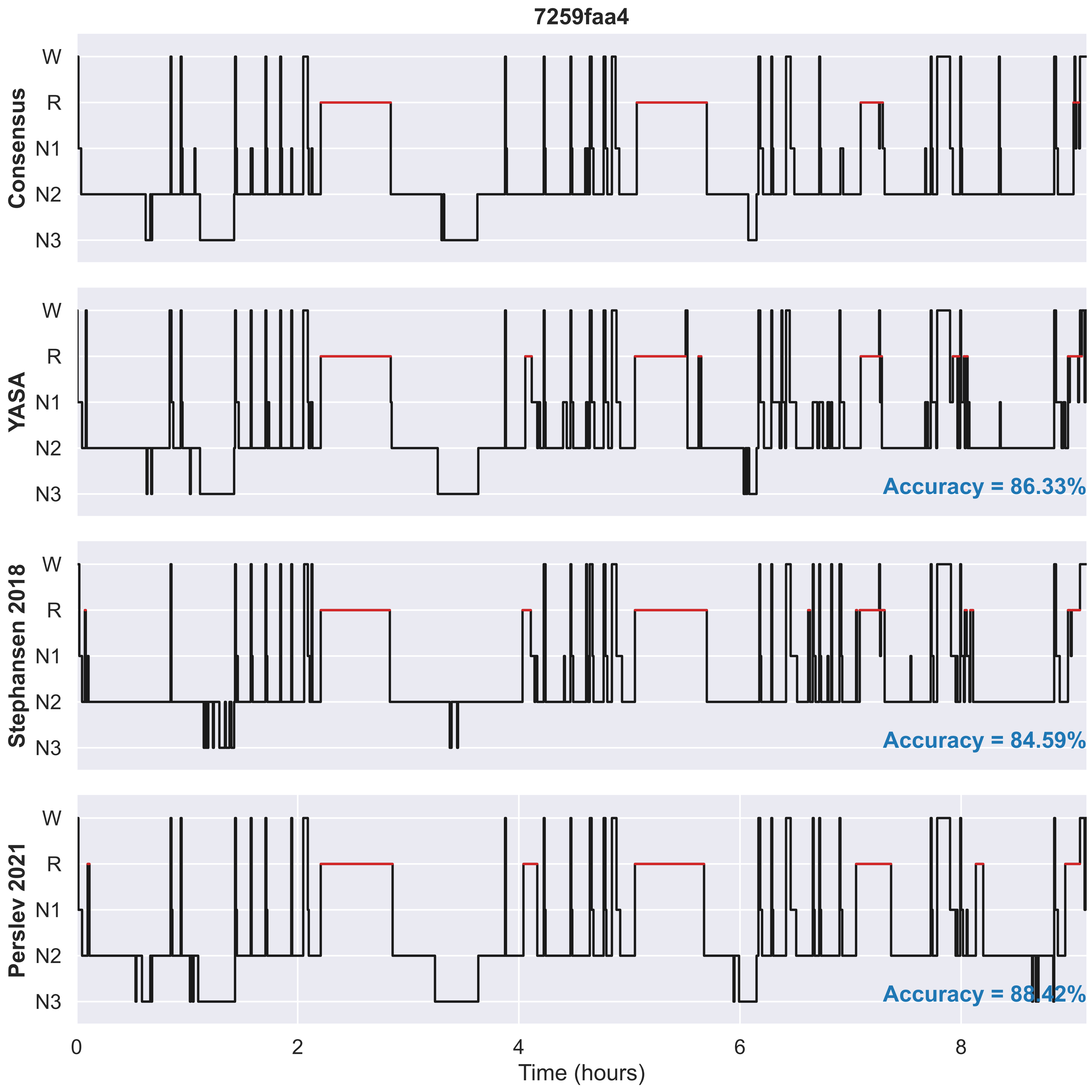

7259faa4
Consensus
YASA
Accuracy = 86.33%
Stephansen 2018
Accuracy = 84.59%
Perslev 2021
Accuracy = 88.42%
W
R
N1
N2
N3
0
2
4
6
8
Time (hours)

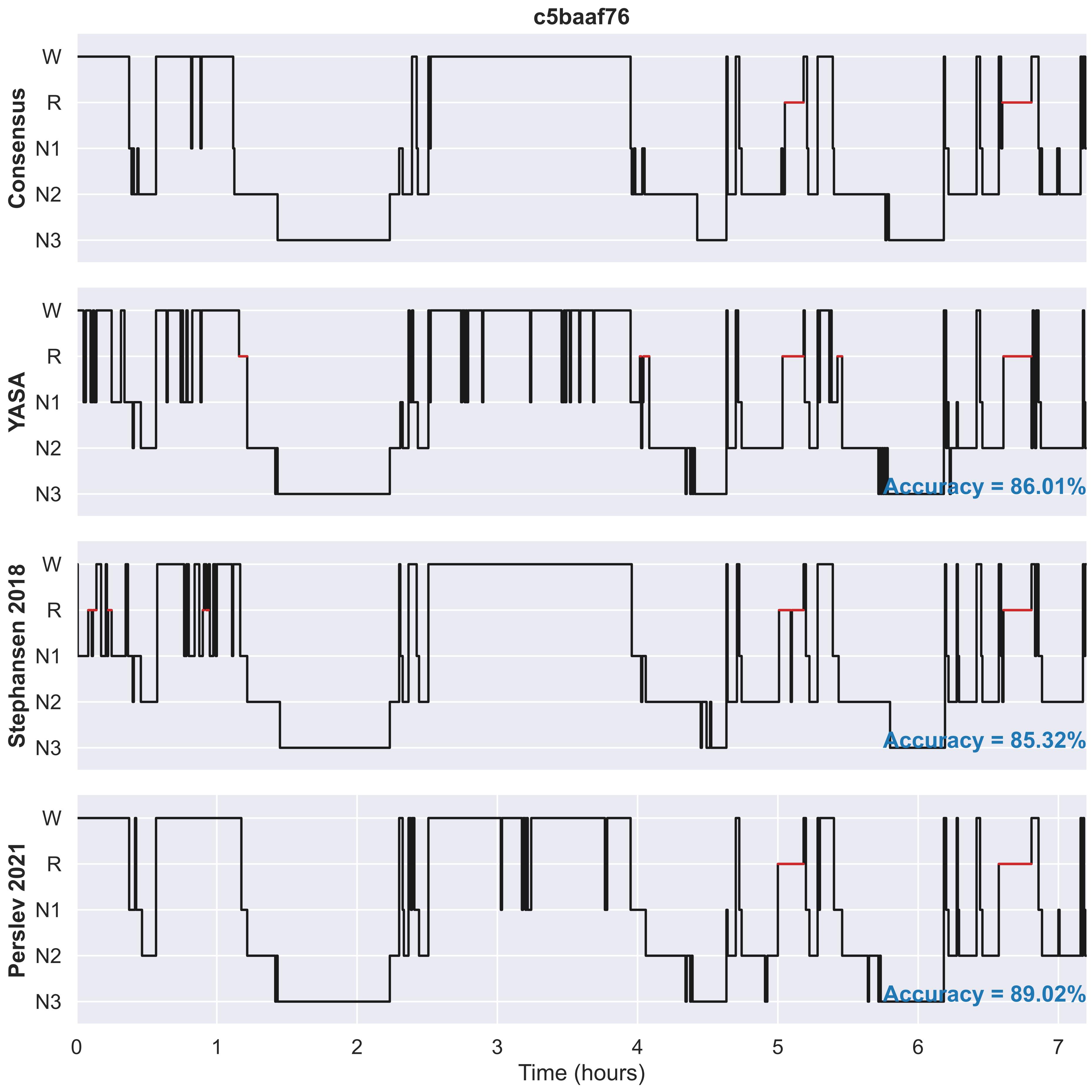
c5baaf76
Consensus
YASA
Stephansen 2018
Perslev 2021
W
R
N1
N2
N3
Accuracy = 86.01%
Accuracy = 85.32%
Accuracy = 89.02%
0
1
2
3
4
5
6
7
Time (hours)

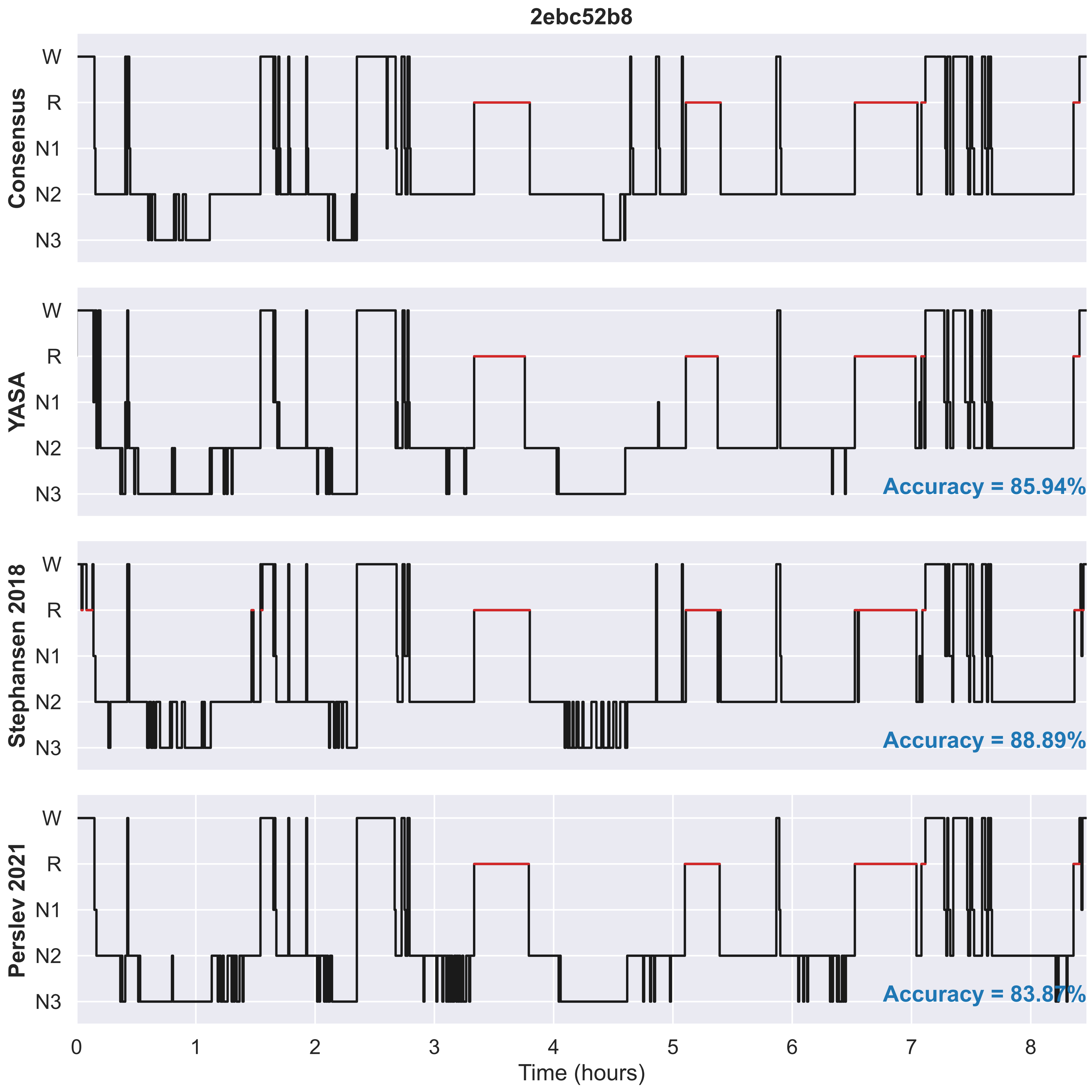

2ebc52b8
Consensus
YASA
Accuracy = 85.94%
Stephansen 2018
Accuracy = 88.89%
Perslev 2021
Accuracy = 83.87%
W
R
N1
N2
N3
0
1
2
3
4
5
6
7
8
Time (hours)

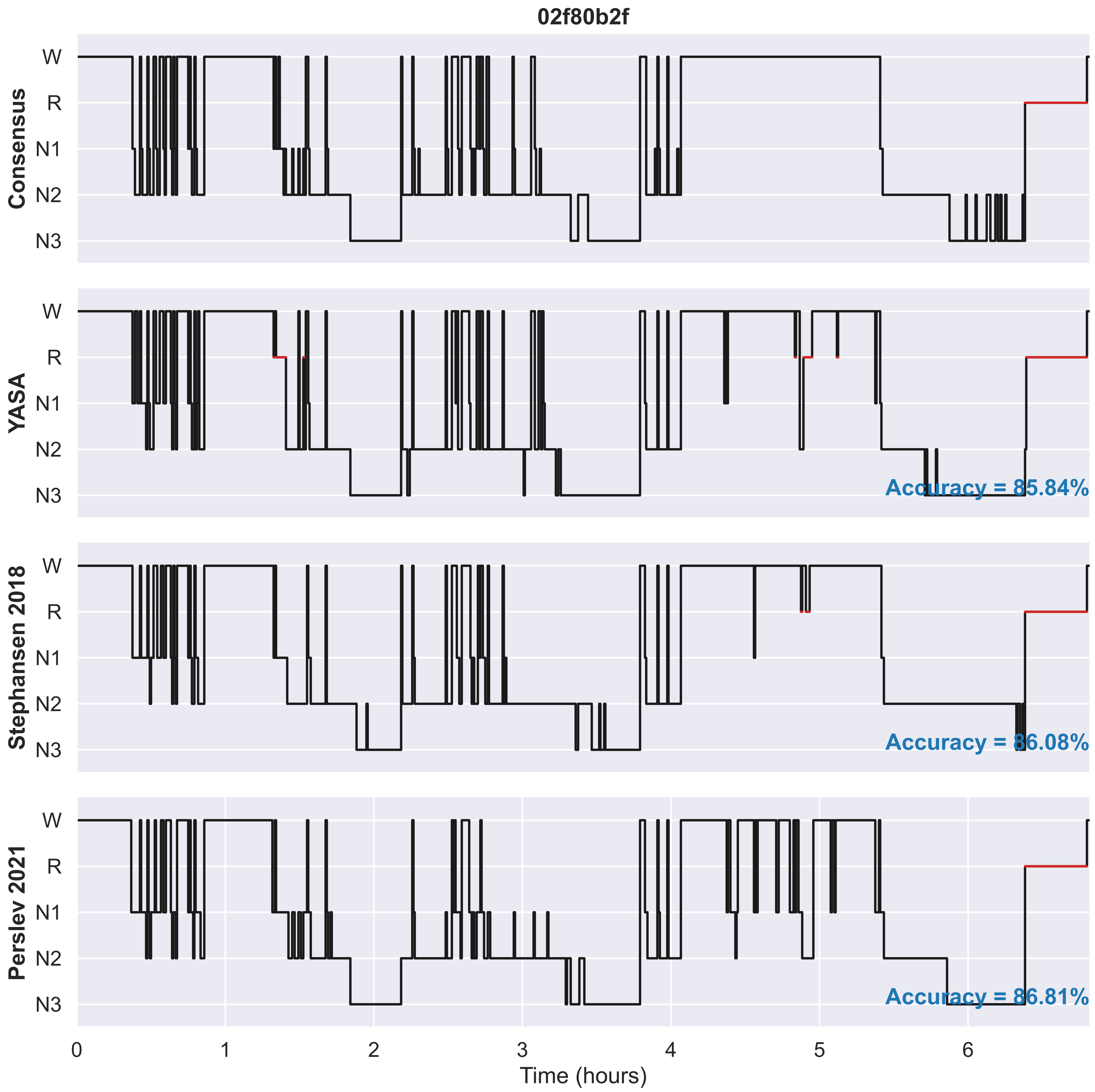

02f80b2f
Consensus
YASA
Accuracy = 85.84%
Stephansen 2018
Accuracy = 86.08%
Perslev 2021
Accuracy = 86.81%
W
R
N1
N2
N3
0
1
2
3
4
5
6
Time (hours)

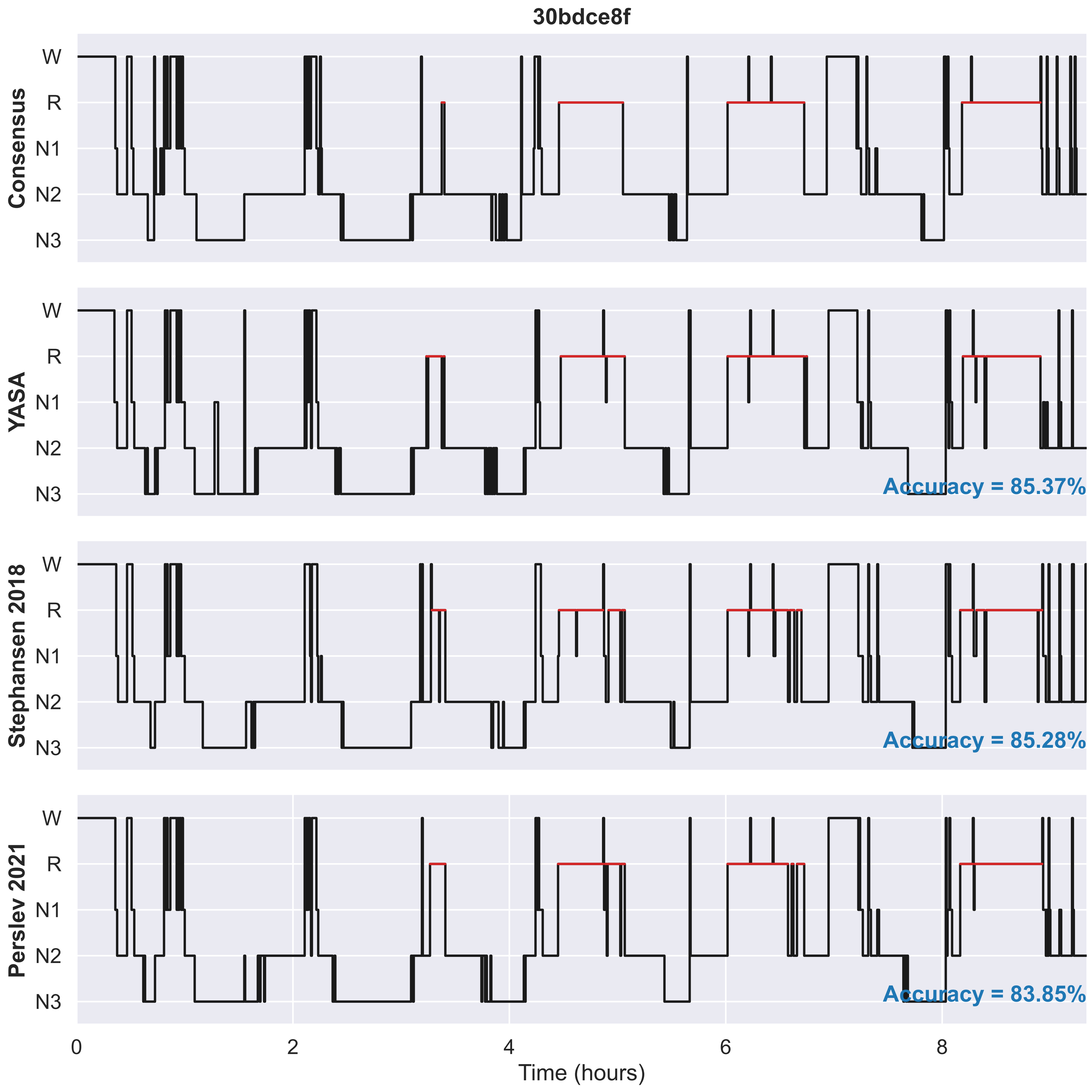
30bdce8f
Consensus
YASA
Stephansen 2018
Perslev 2021
W
R
N1
N2
N3
Accuracy = 85.37%
Accuracy = 85.28%
Accuracy = 83.85%
0
2
4
6
8
Time (hours)

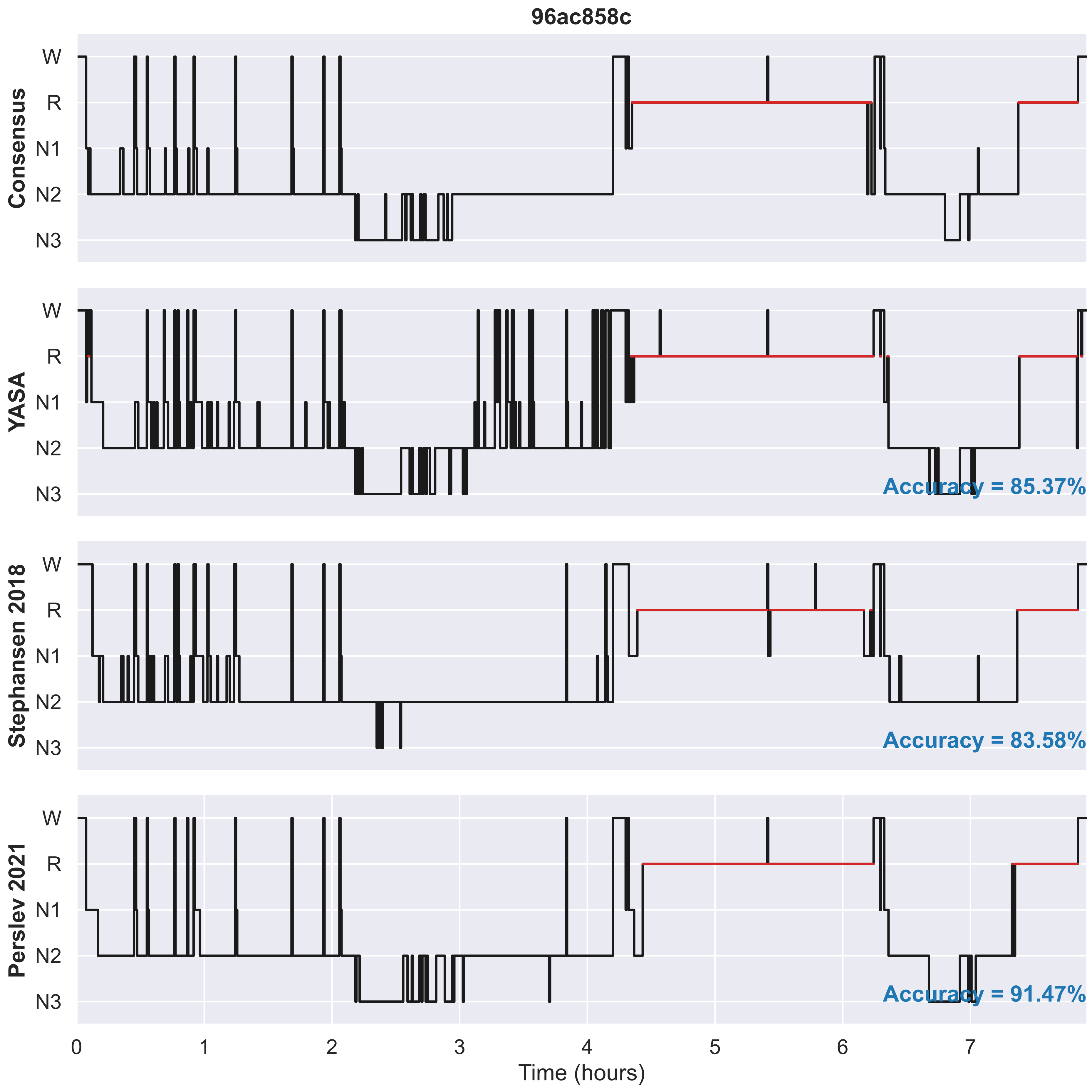

96ac858c
Consensus
YASA
Stephansen 2018
Perslev 2021
W
R
N1
N2
N3
Accuracy = 85.37%
Accuracy = 83.58%
Accuracy = 91.47%
0
1
2
3
4
5
6
7
Time (hours)

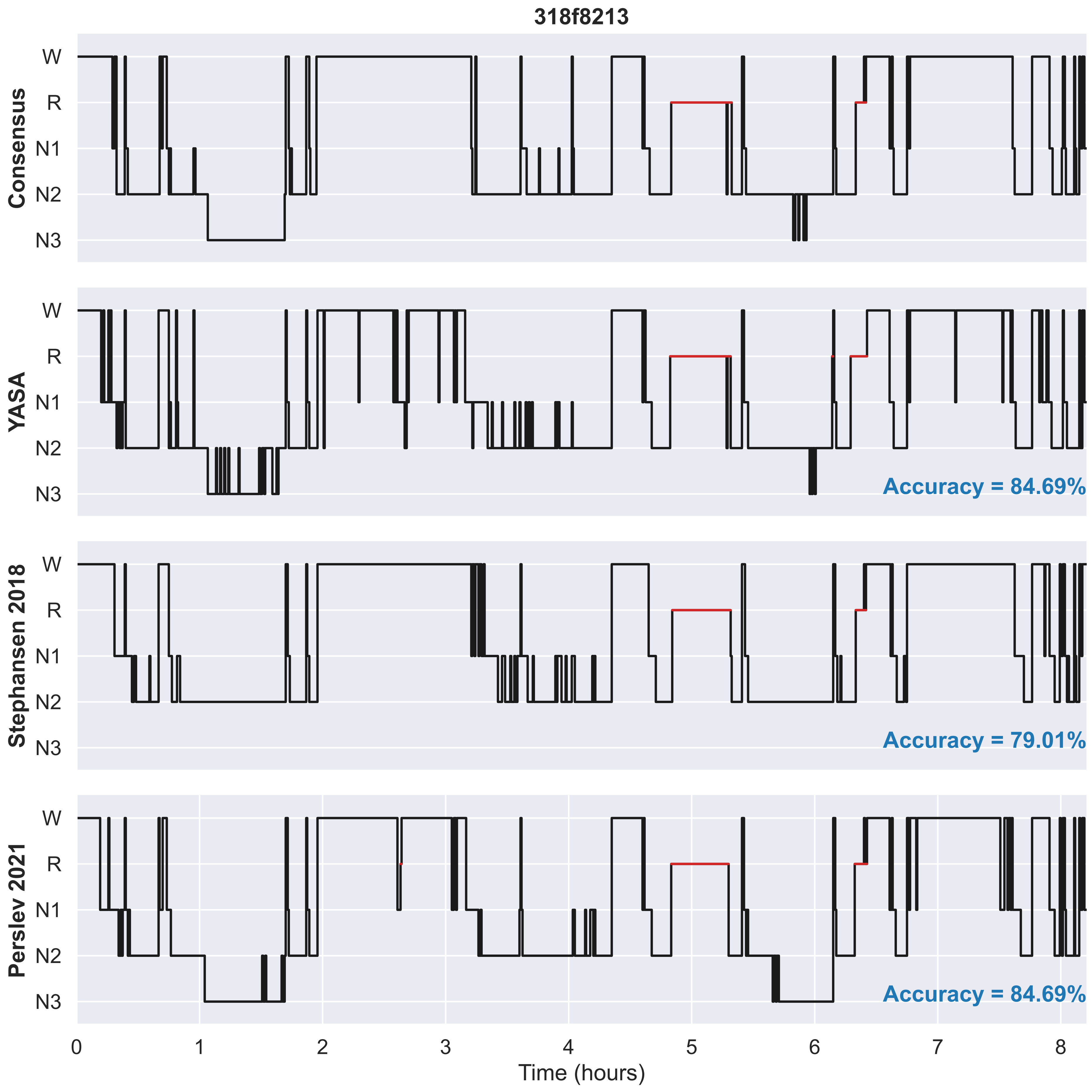
318f8213
Consensus
YASA
Stephansen 2018
Perslev 2021
W
R
N1
N2
N3
Accuracy = 84.69%
Accuracy = 79.01%
Accuracy = 84.69%
0
1
2
3
4
5
6
7
8
Time (hours)

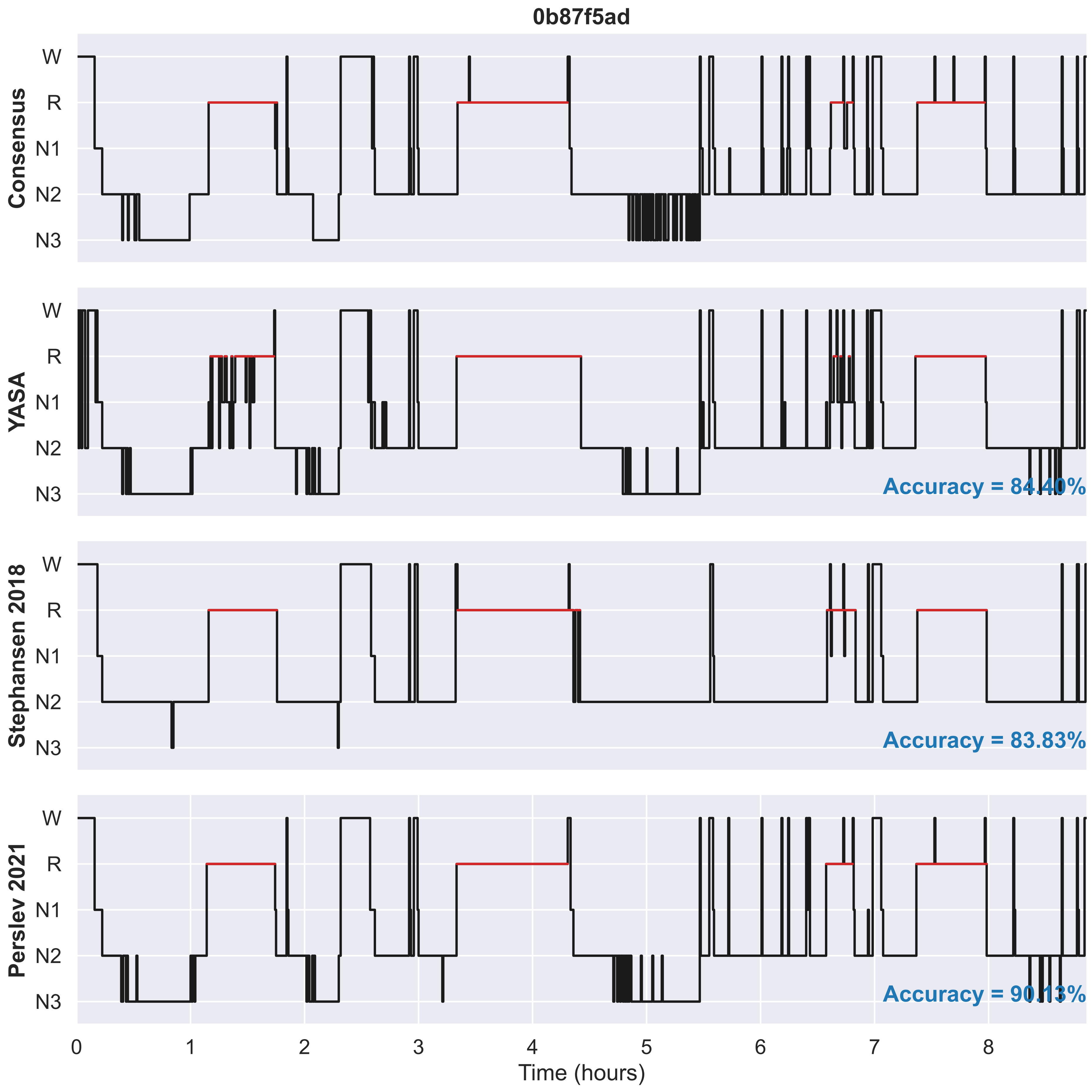
0b87f5ad
Consensus
YASA
Stephansen 2018
Perslev 2021
W
R
N1
N2
N3
Accuracy = 84.40%
Accuracy = 83.83%
Accuracy = 90.13%
0
1
2
3
4
5
6
7
8
Time (hours)

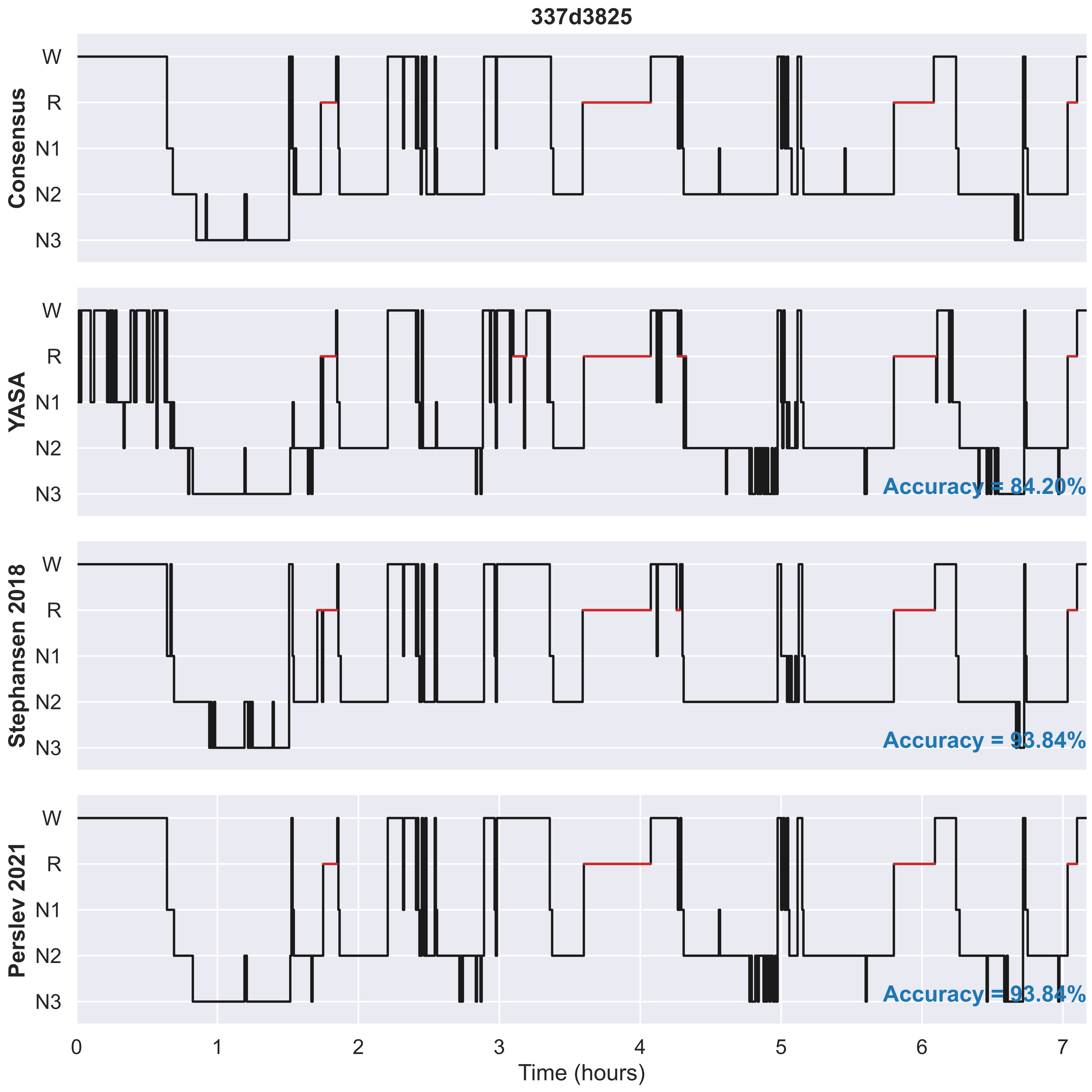
337d3825
Consensus
YASA
Stephansen 2018
Perslev 2021
W
R
N1
N2
N3
Accuracy = 84.20%
Accuracy = 93.84%
Accuracy = 93.84%
0
1
2
3
4
5
6
7
Time (hours)

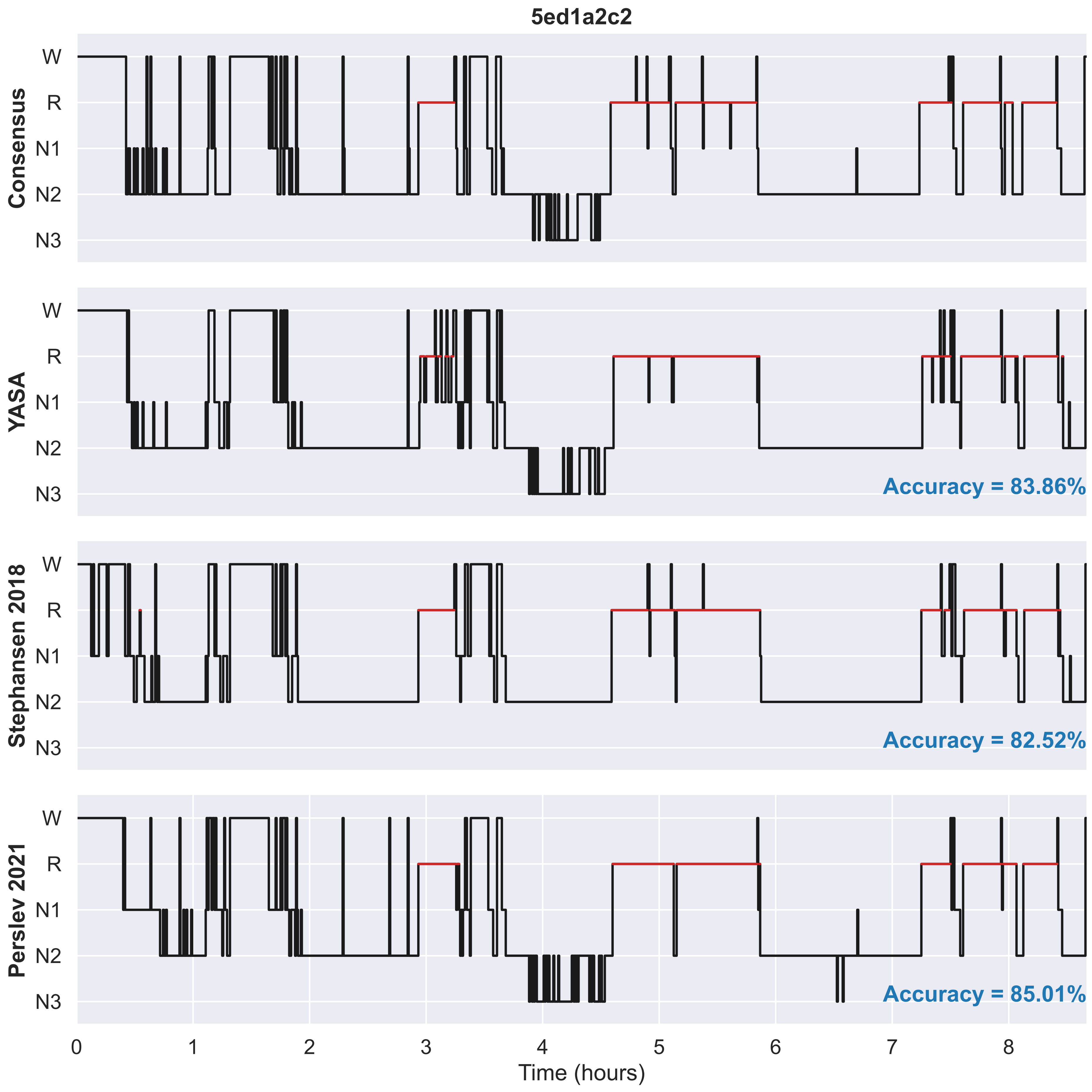

5ed1a2c2
Consensus
YASA
Stephansen 2018
Perslev 2021
W
R
N1
N2
N3
Accuracy = 83.86%
Accuracy = 82.52%
Accuracy = 85.01%
0
1
2
3
4
5
6
7
8
Time (hours)

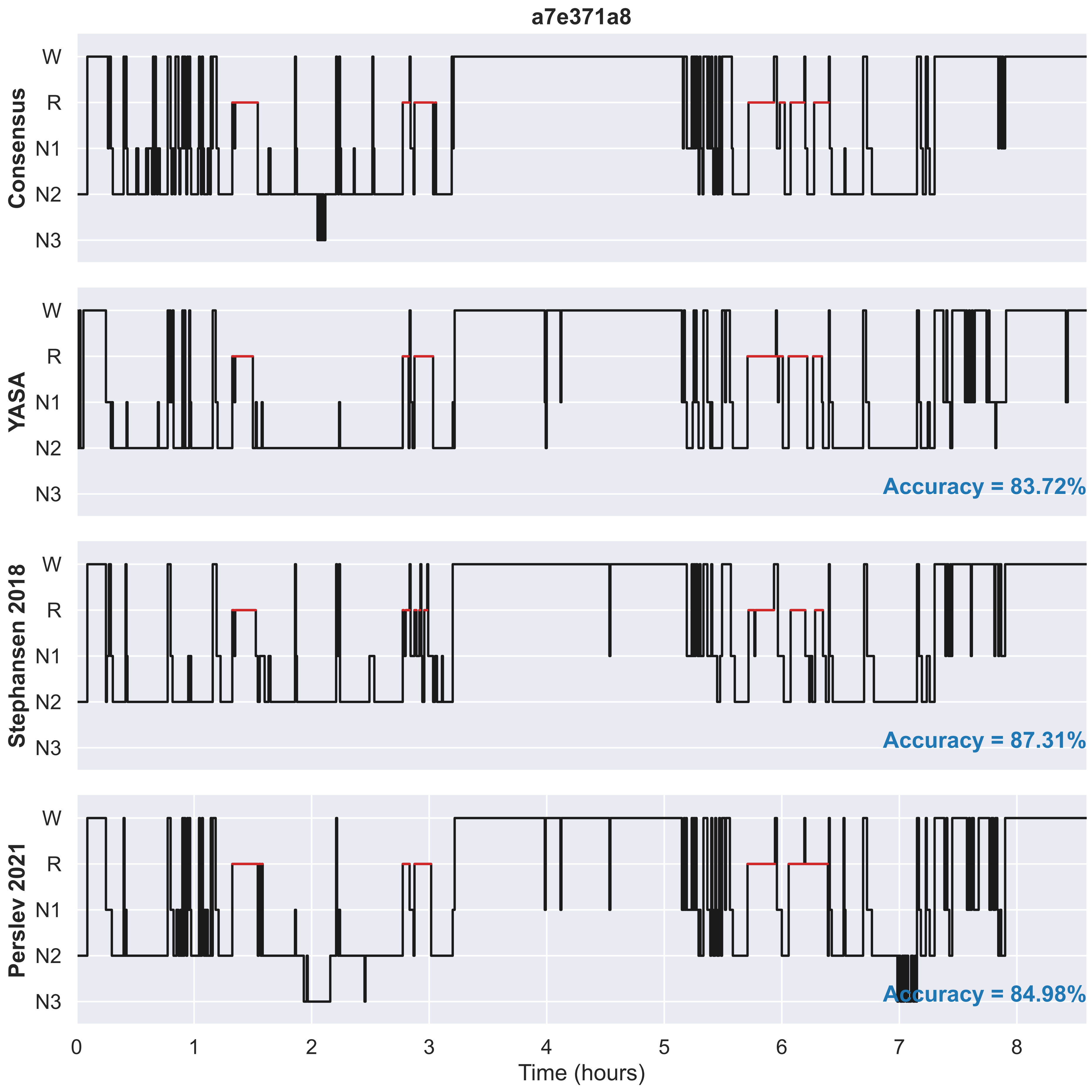

a7e371a8
Consensus
YASA
Stephansen 2018
Perslev 2021
W
R
N1
N2
N3
Accuracy = 83.72%
Accuracy = 87.31%
Accuracy = 84.98%
0
1
2
3
4
5
6
7
8
Time (hours)

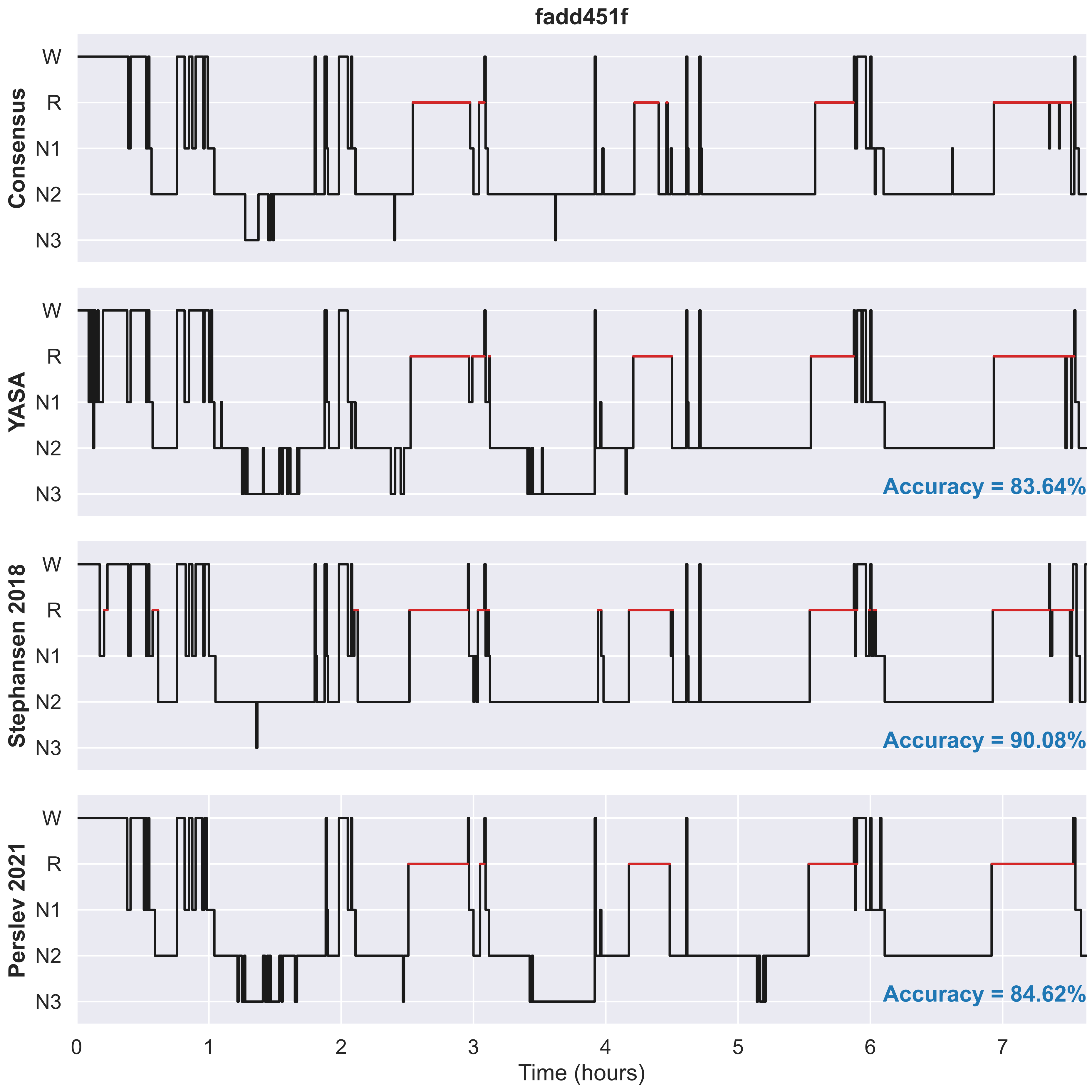
fadd451f
Consensus
YASA
Accuracy = 83.64%
Stephansen 2018
Accuracy = 90.08%
Perslev 2021
Accuracy = 84.62%
W
R
N1
N2
N3
0
1
2
3
4
5
6
7
Time (hours)

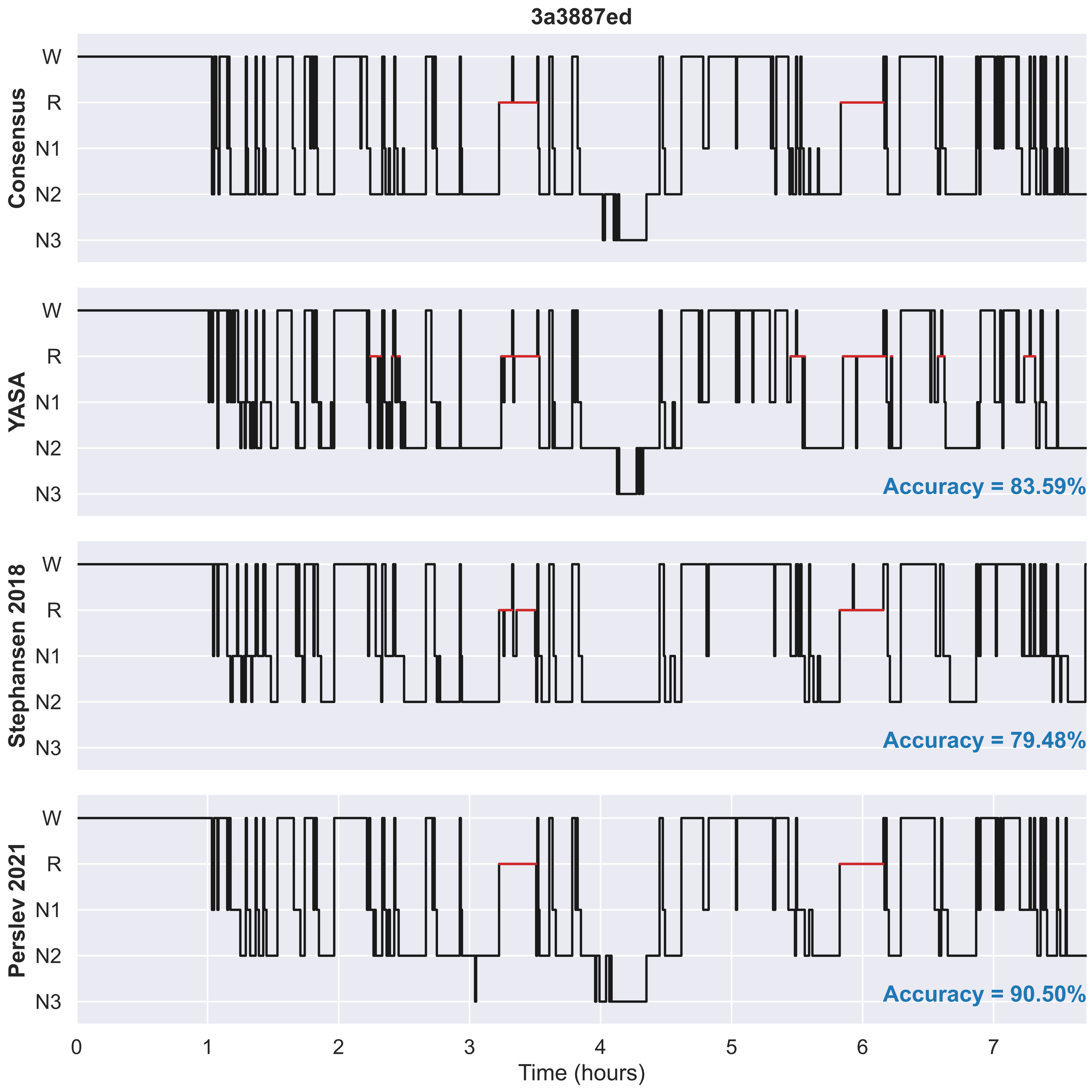
3a3887ed
Consensus
YASA
Stephansen 2018
Perslev 2021
W
R
N1
N2
N3
Accuracy = 83.59%
Accuracy = 79.48%
Accuracy = 90.50%
0
1
2
3
4
5
6
7
Time (hours)

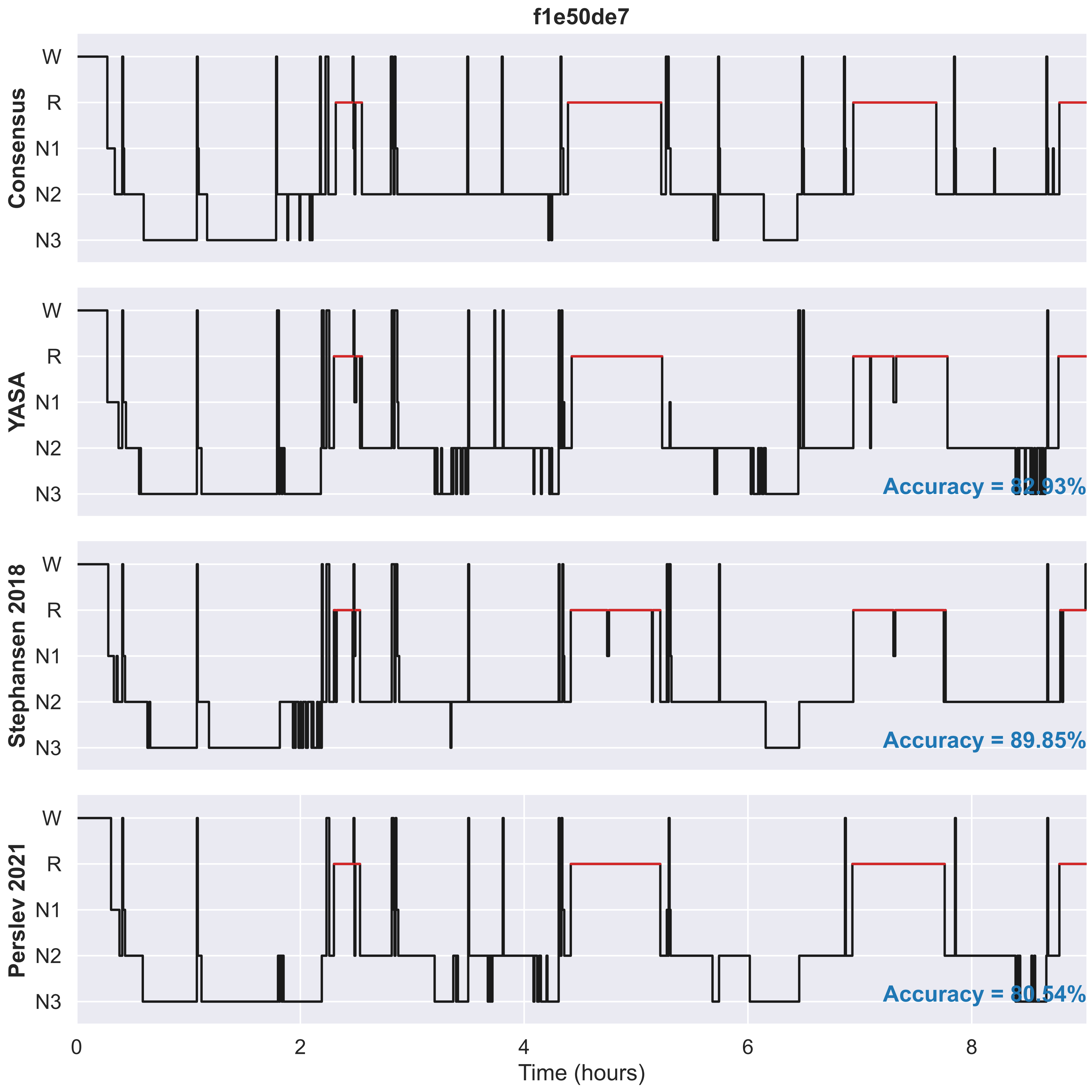
f1e50de7
Consensus
YASA
Accuracy = 82.93%
Stephansen 2018
Accuracy = 89.85%
Perslev 2021
Accuracy = 80.54%
W
R
N1
N2
N3
0
2
4
6
8
Time (hours)

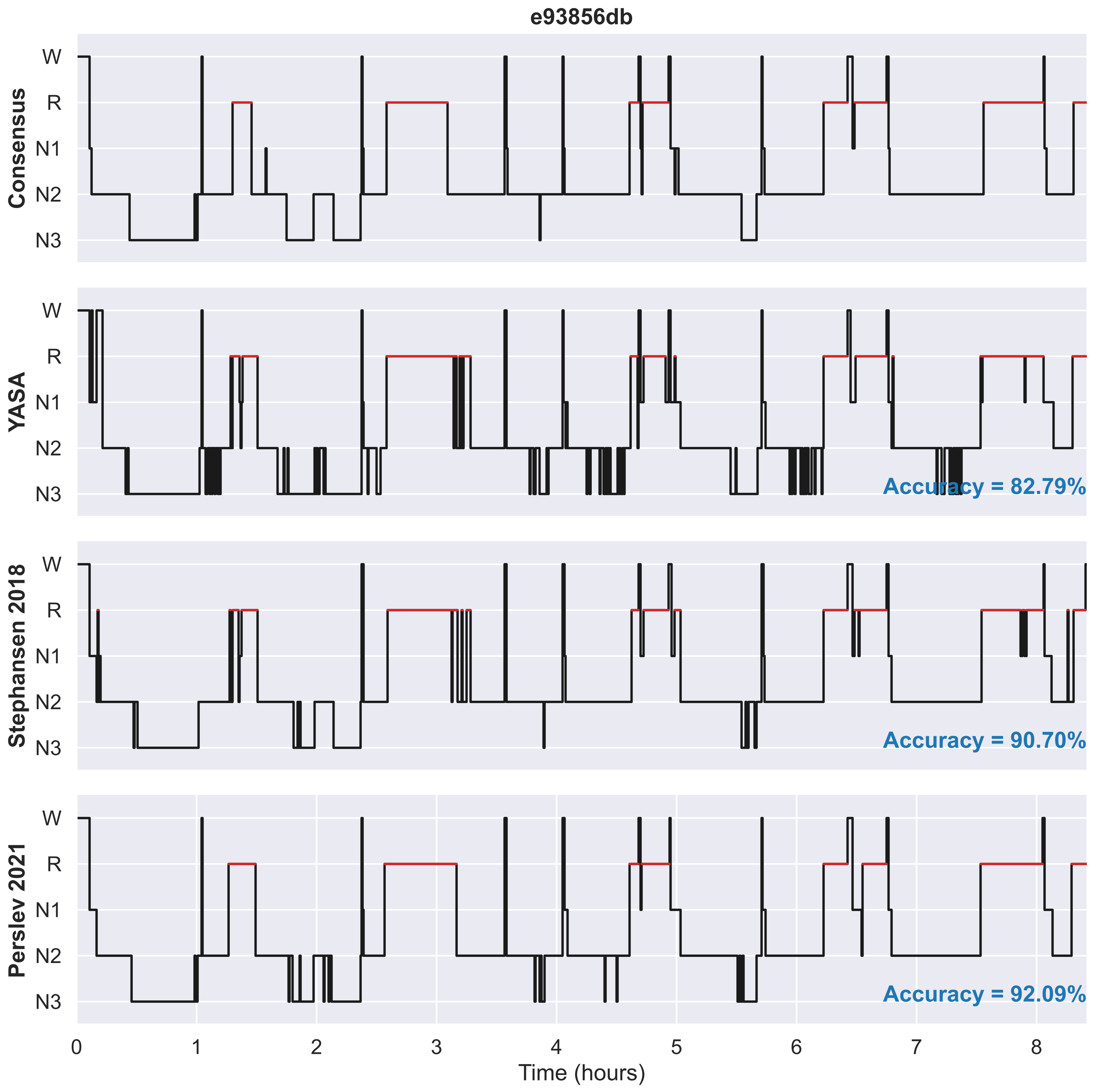

e93856db
Consensus
YASA
Stephansen 2018
Perslev 2021
W
R
N1
N2
N3
Accuracy = 82.79%
Accuracy = 90.70%
Accuracy = 92.09%
0
1
2
3
4
5
6
7
8
Time (hours)

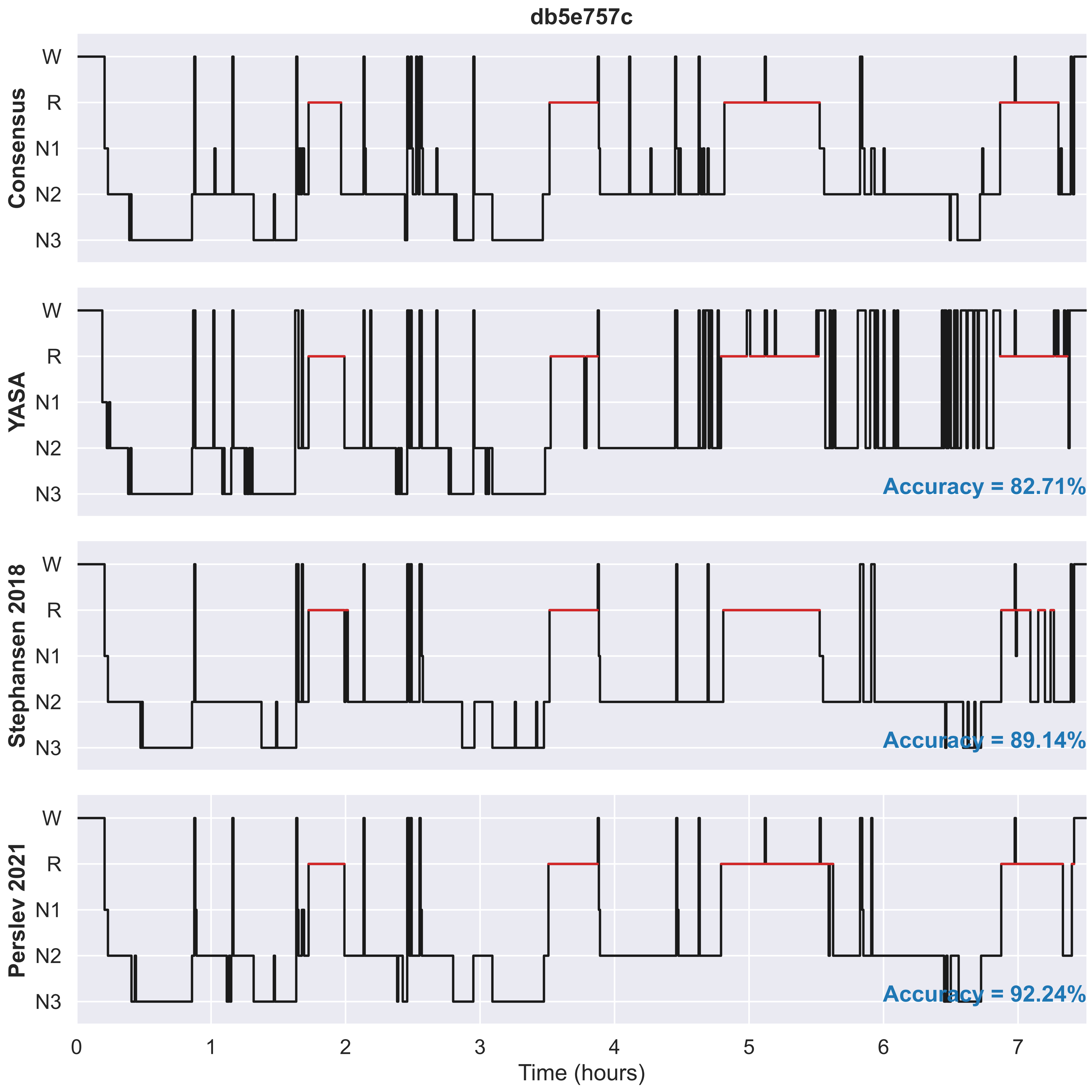

db5e757c
Consensus
YASA
Stephansen 2018
Perslev 2021
W
R
N1
N2
N3
Accuracy = 82.71%
Accuracy = 89.14%
Accuracy = 92.24%
0
1
2
3
4
5
6
7
Time (hours)

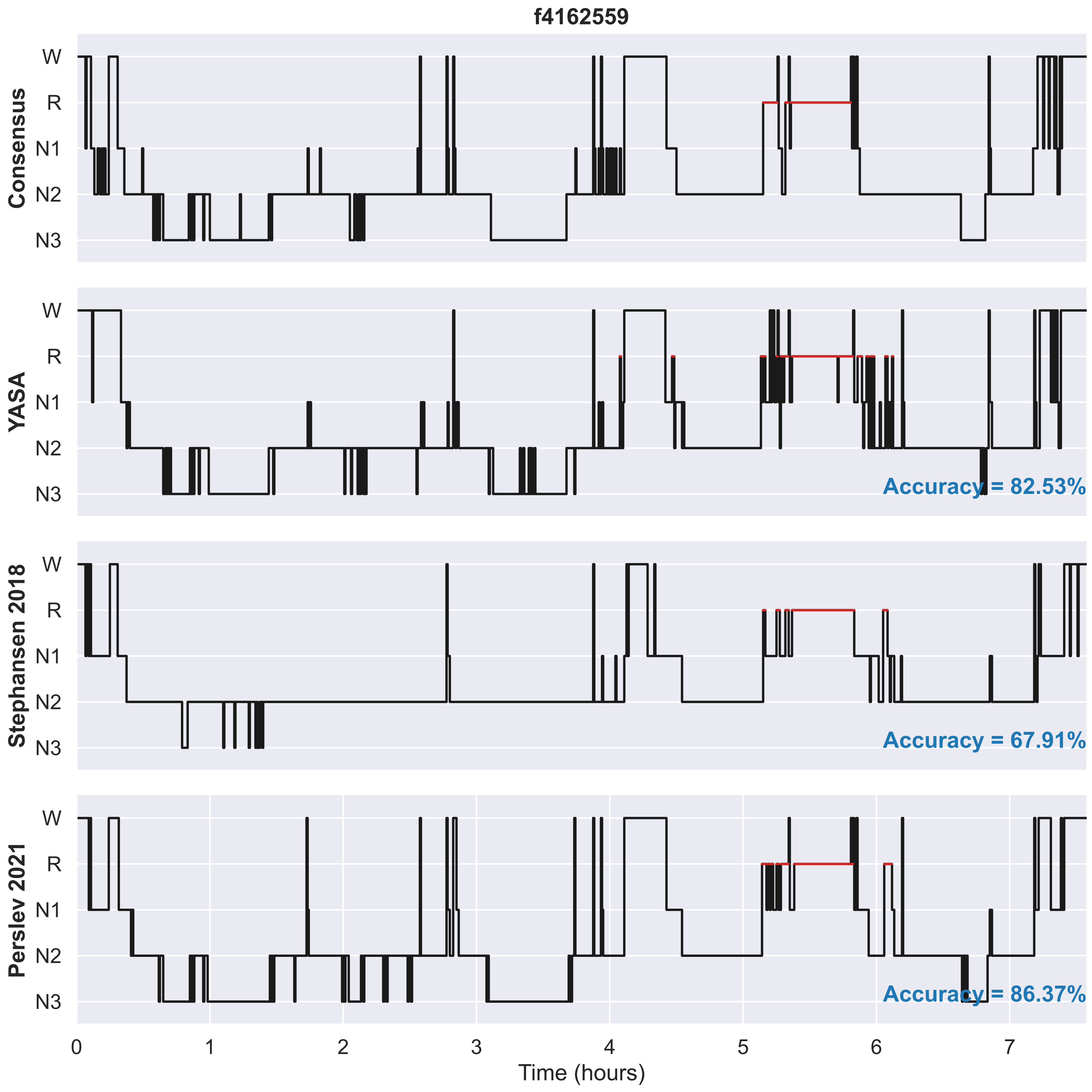
f4162559
Consensus
YASA
Accuracy = 82.53%
Stephansen 2018
Accuracy = 67.91%
Perslev 2021
Accuracy = 86.37%
W
R
N1
N2
N3
0
1
2
3
4
5
6
7
Time (hours)

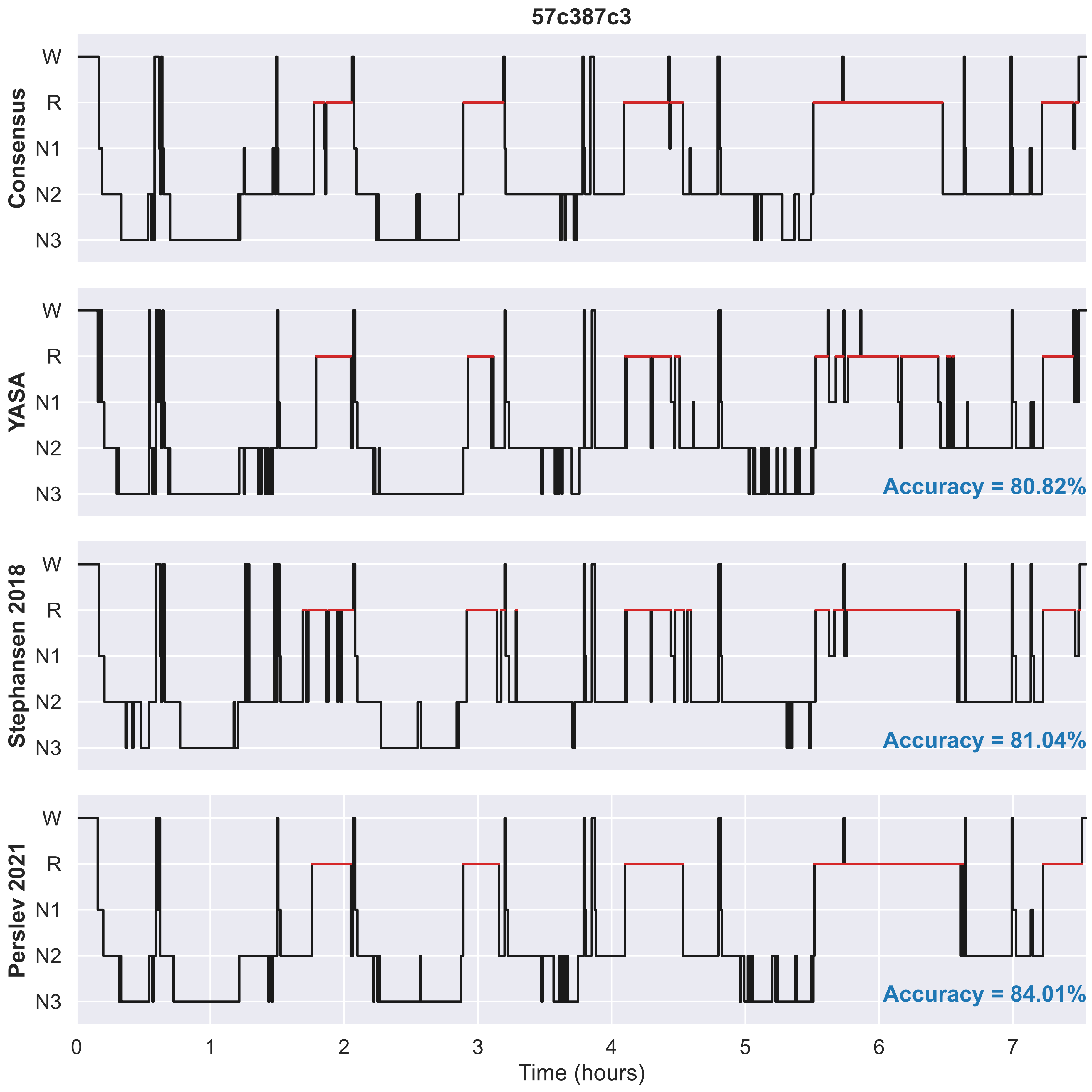
57c387c3
Consensus
YASA
Accuracy = 80.82%
Stephansen 2018
Accuracy = 81.04%
Perslev 2021
Accuracy = 84.01%
W
R
N1
N2
N3
Time (hours)

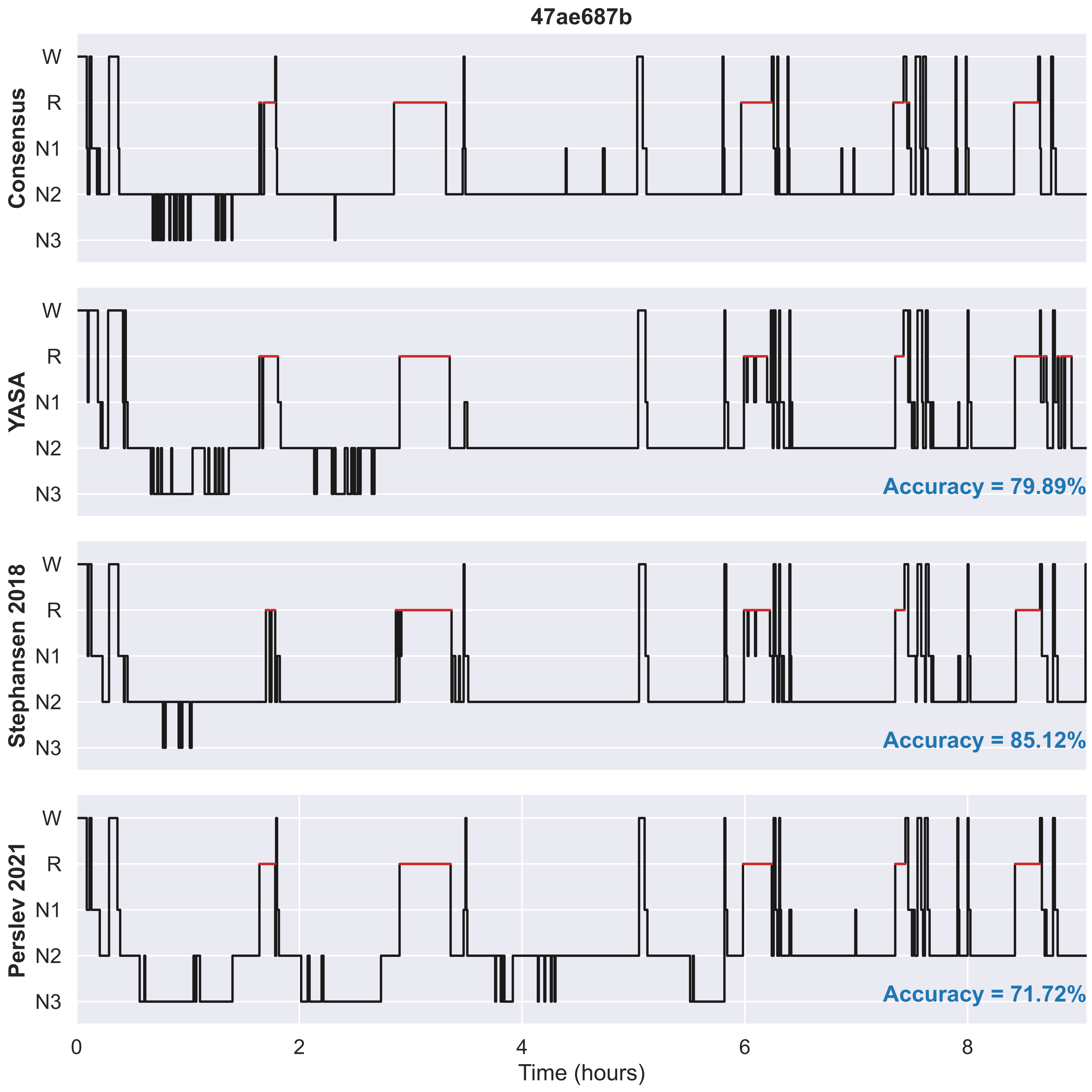

47ae687b
Consensus
YASA
Accuracy = 79.89%
Stephansen 2018
Accuracy = 85.12%
Perslev 2021
Accuracy = 71.72%
W
R
N1
N2
N3
0
2
4
6
8
Time (hours)

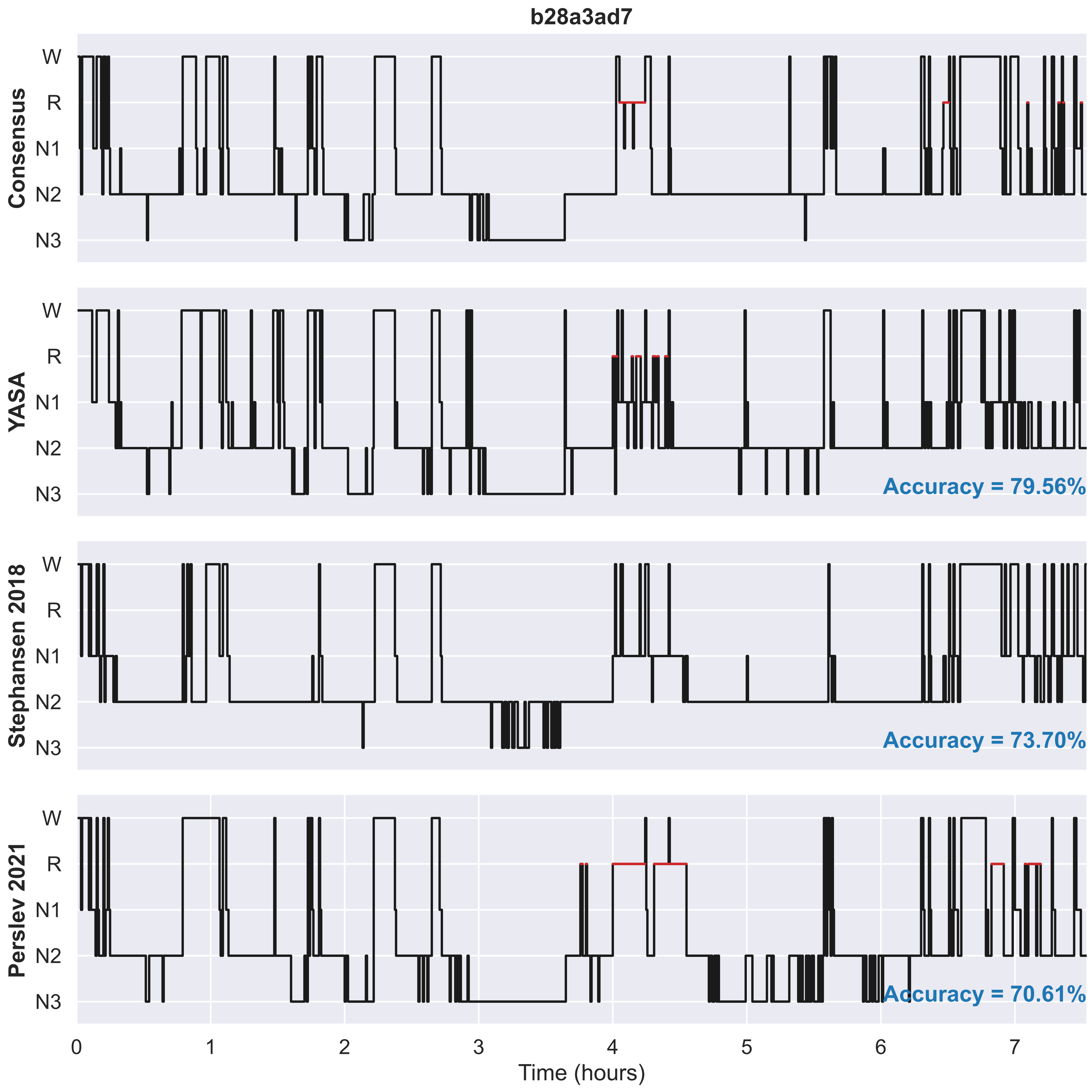

b28a3ad7
Consensus
YASA
Stephansen 2018
Perslev 2021
W
R
N1
N2
N3
Accuracy = 79.56%
Accuracy = 73.70%
Accuracy = 70.61%
0
1
2
3
4
5
6
7
Time (hours)

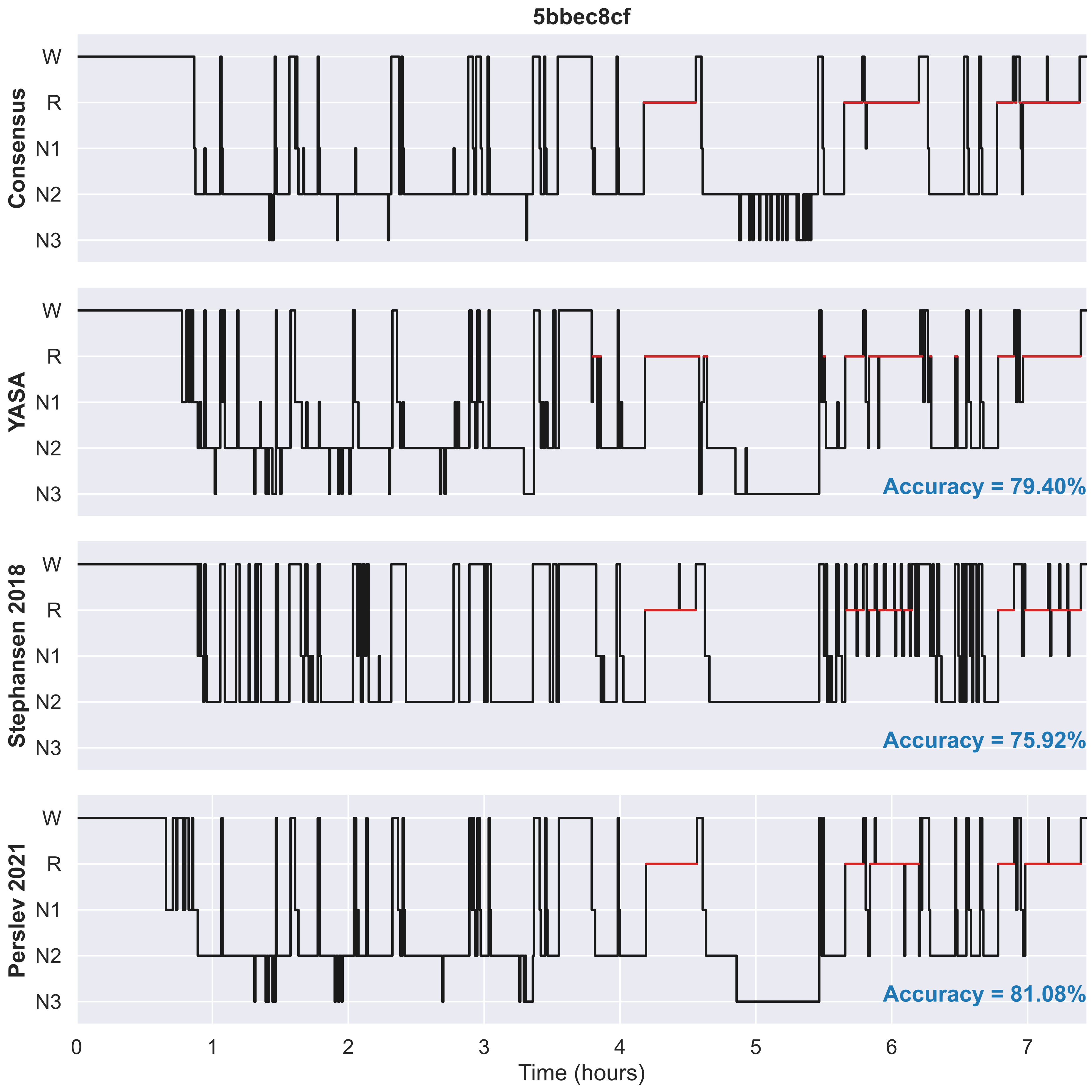
5bbec8cf
Consensus
YASA
Accuracy = 79.40%
Stephansen 2018
Accuracy = 75.92%
Perslev 2021
Accuracy = 81.08%
W
R
N1
N2
N3
0
1
2
3
4
5
6
7
Time (hours)

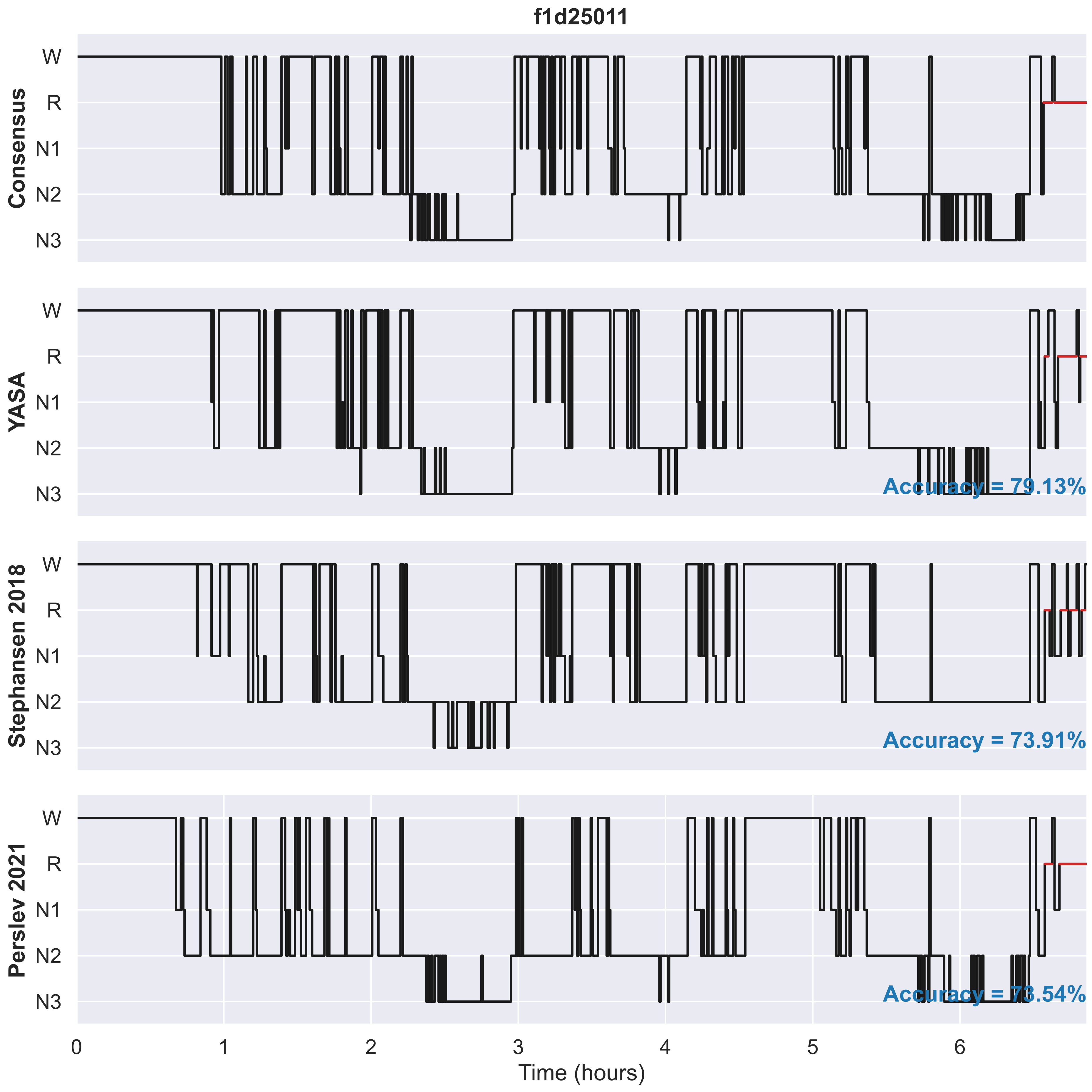

f1d25011
Consensus
W
R
N1
N2
N3
YASA
Accuracy = 79.13%
Stephansen 2018
Accuracy = 73.91%
Perslev 2021
Accuracy = 73.54%
0
1
2
3
4
5
6
Time (hours)

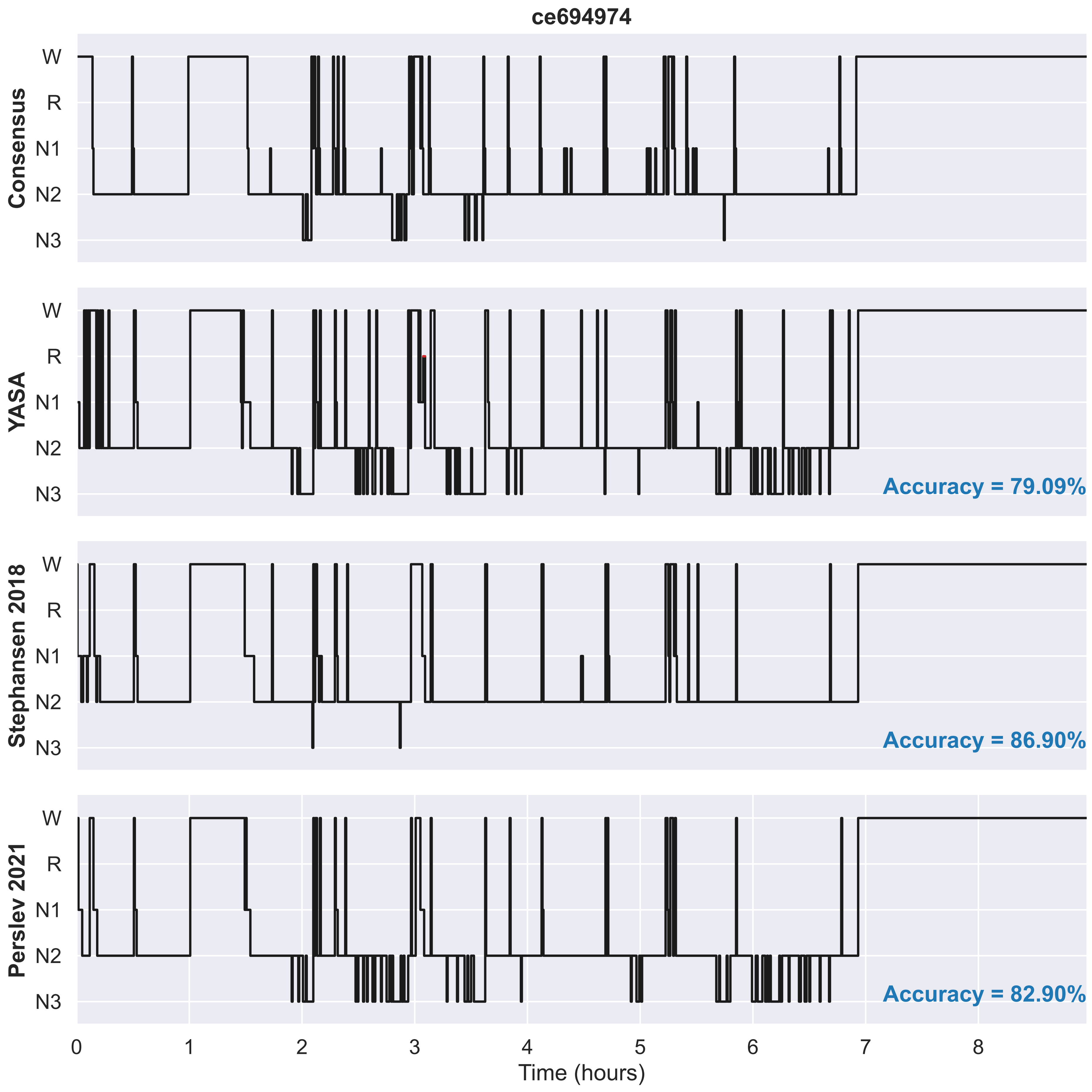
ce694974
Consensus
YASA
Stephansen 2018
Perslev 2021
W
R
N1
N2
N3
Accuracy = 79.09%
Accuracy = 86.90%
Accuracy = 82.90%
0
1
2
3
4
5
6
7
8
Time (hours)

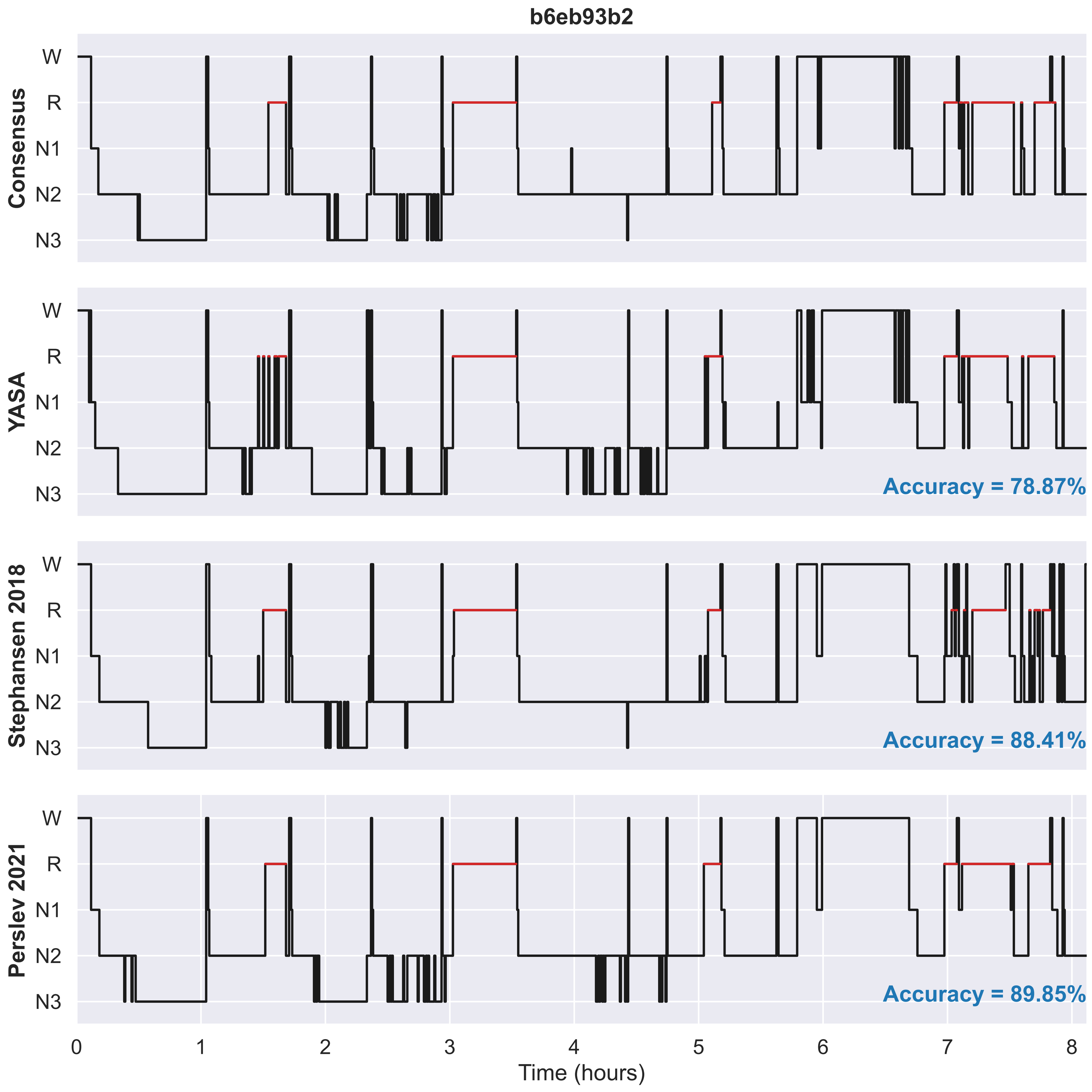

b6eb93b2
Consensus
YASA
Accuracy = 78.87%
Stephansen 2018
Accuracy = 88.41%
Perslev 2021
Accuracy = 89.85%
W
R
N1
N2
N3
0
1
2
3
4
5
6
7
8
Time (hours)

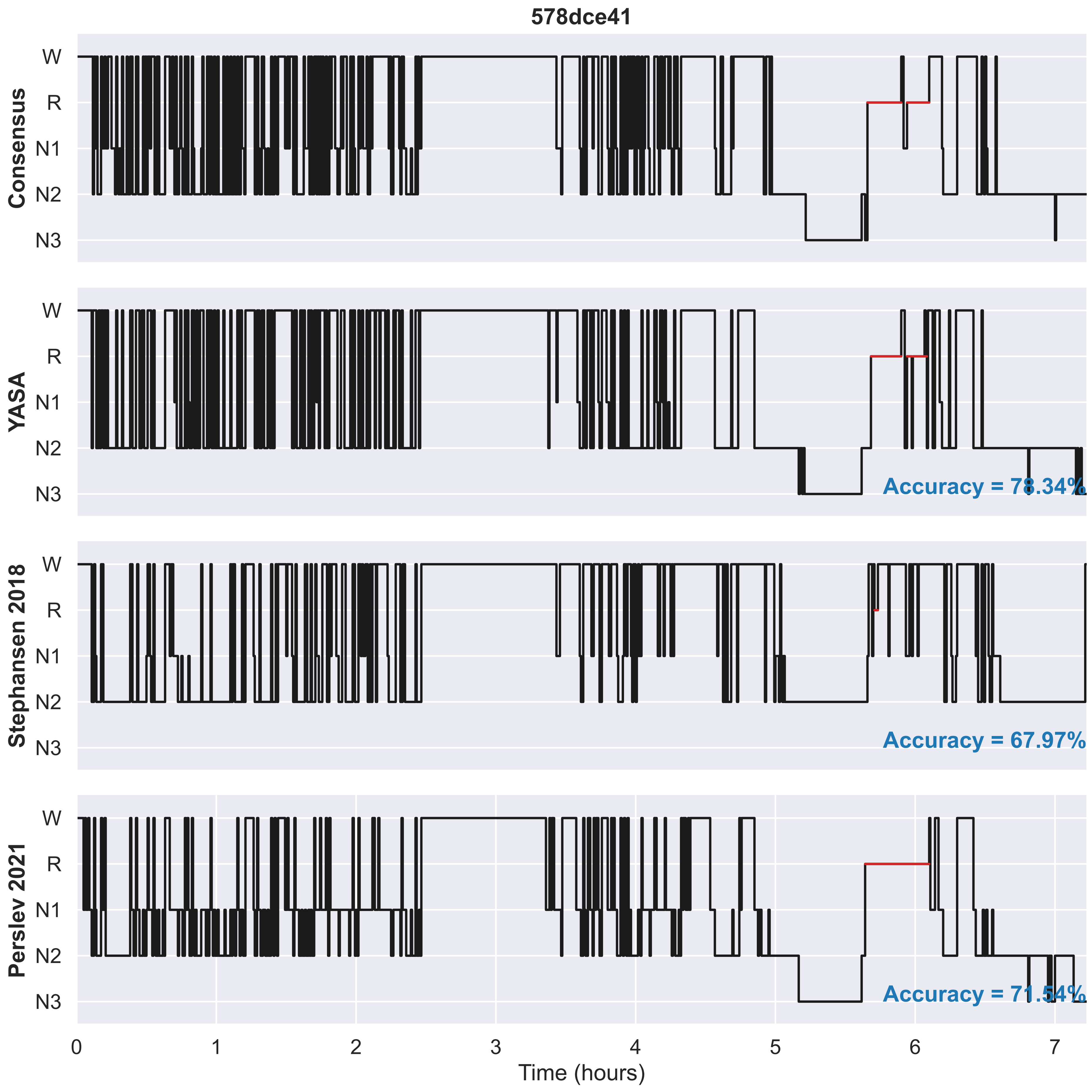
578dce41
Consensus
YASA
Stephansen 2018
Persler 2021
W
R
N1
N2
N3
Accuracy = 78.34%
Accuracy = 67.97%
Accuracy = 71.54%
0
1
2
3
4
5
6
7
Time (hours)

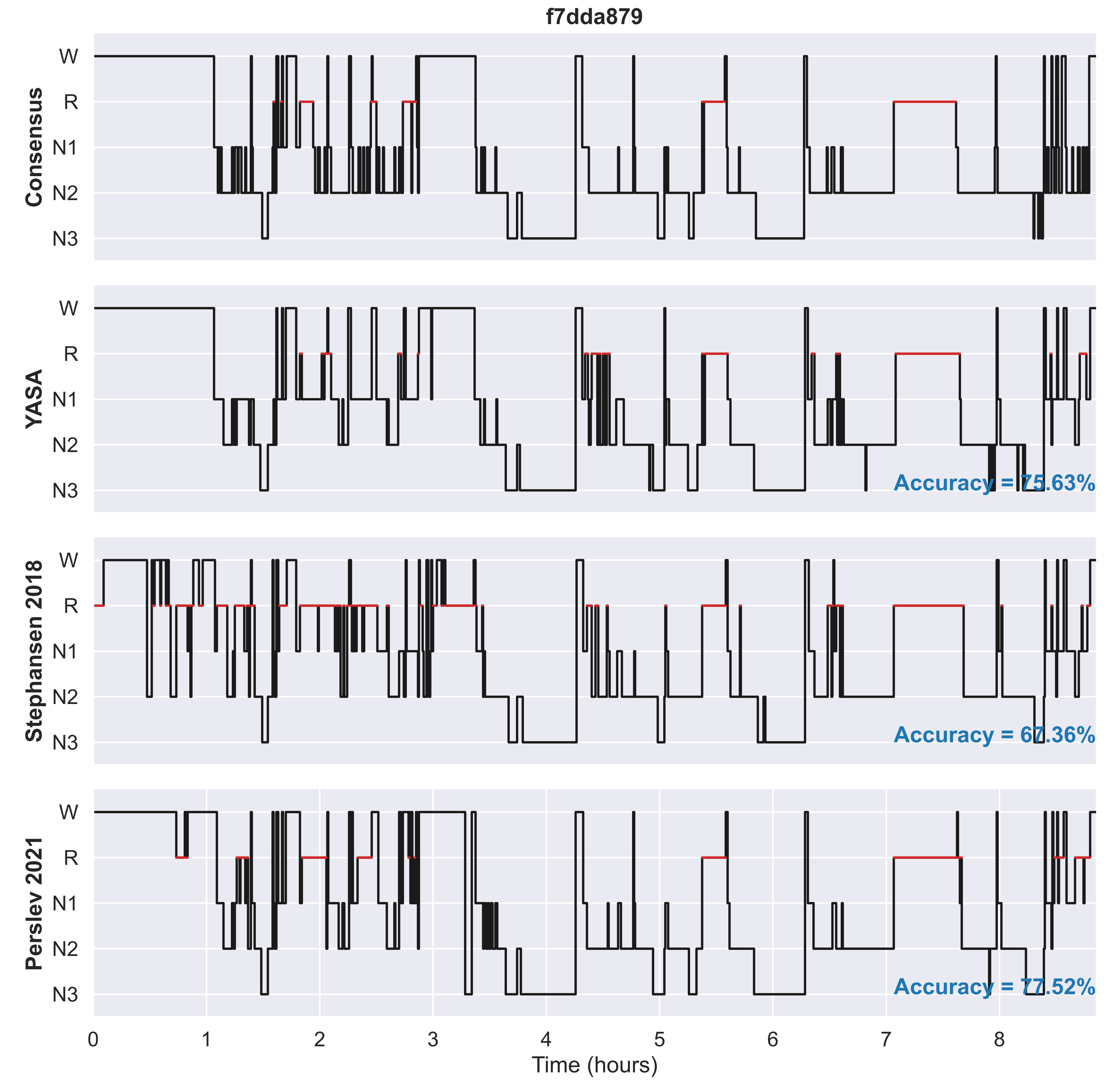
f7dda879
Consensus
YASA
Stephansen 2018
Perslev 2021
W
R
N1
N2
N3
Accuracy = 75.63%
Accuracy = 67.36%
Accuracy = 77.52%
0
1
2
3
4
5
6
7
8
Time (hours)

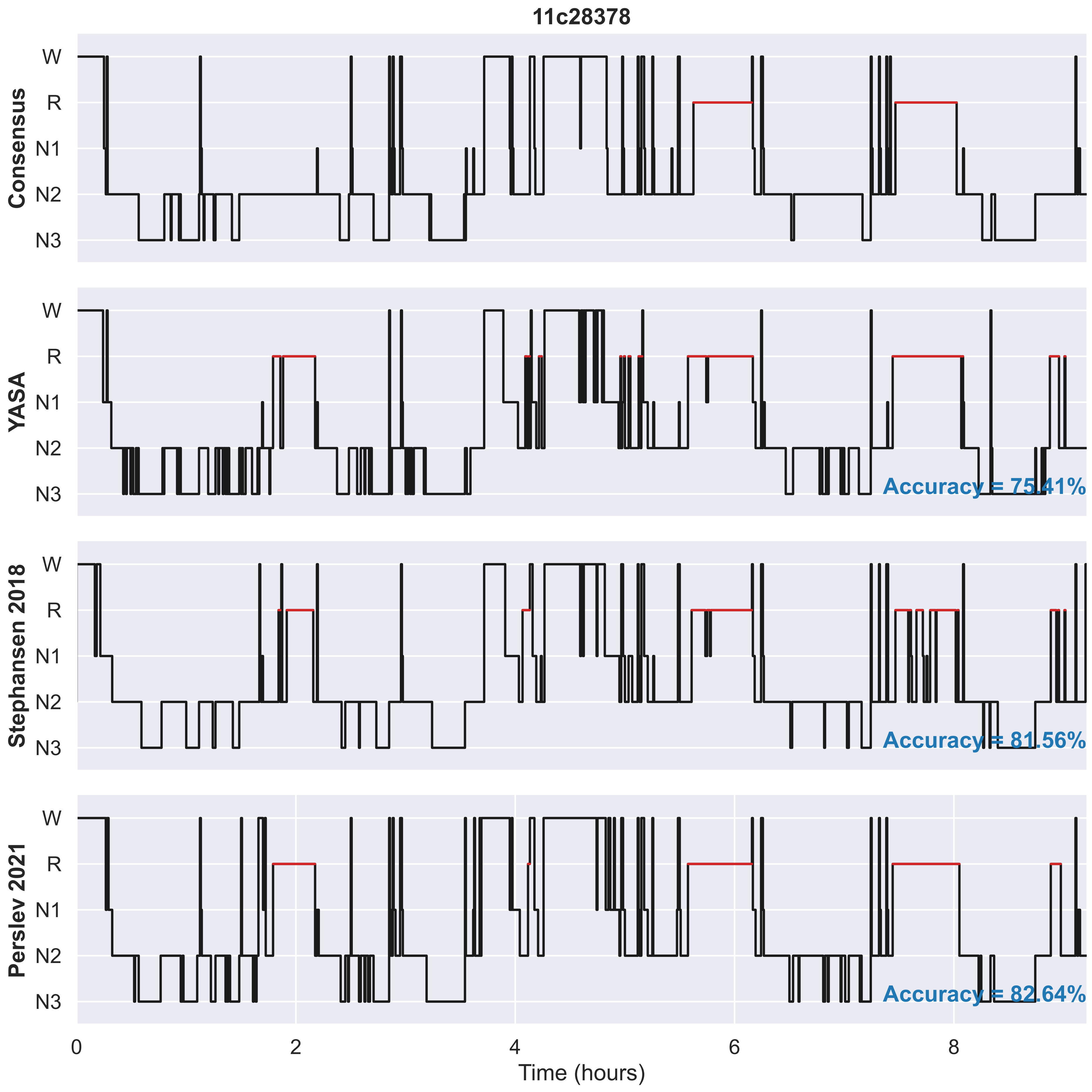
11c28378
Consensus
YASA
Stephansen 2018
Perslev 2021
W
R
N1
N2
N3
Accuracy = 75.41%
Accuracy = 81.56%
Accuracy = 82.64%
0
2
4
6
8
Time (hours)

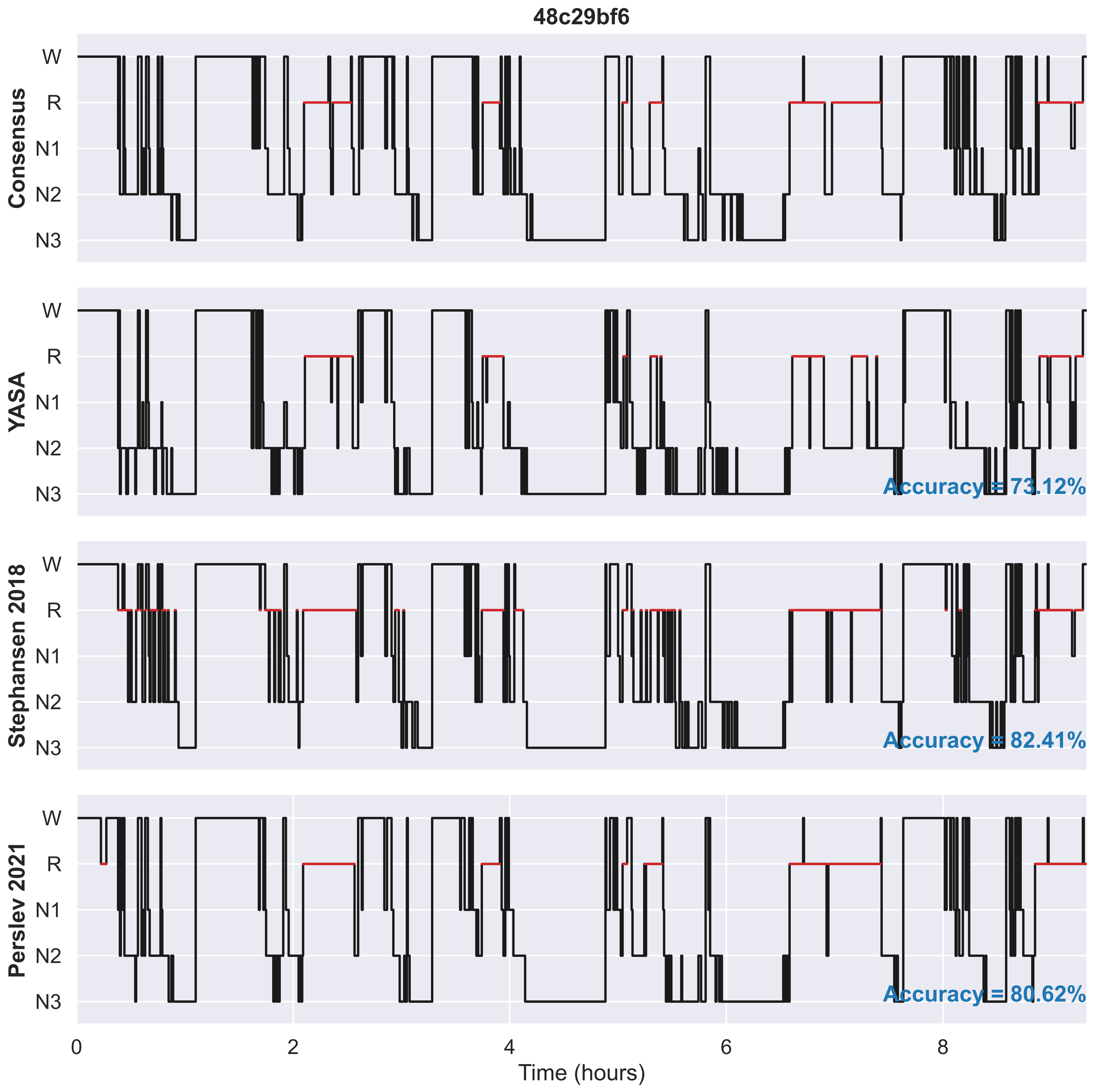
48c29bf6
Consensus
YASA
Stephansen 2018
Perslev 2021
W
R
N1
N2
N3
Accuracy = 73.12%
Accuracy = 82.41%
Accuracy = 80.62%
0
2
4
6
8
Time (hours)

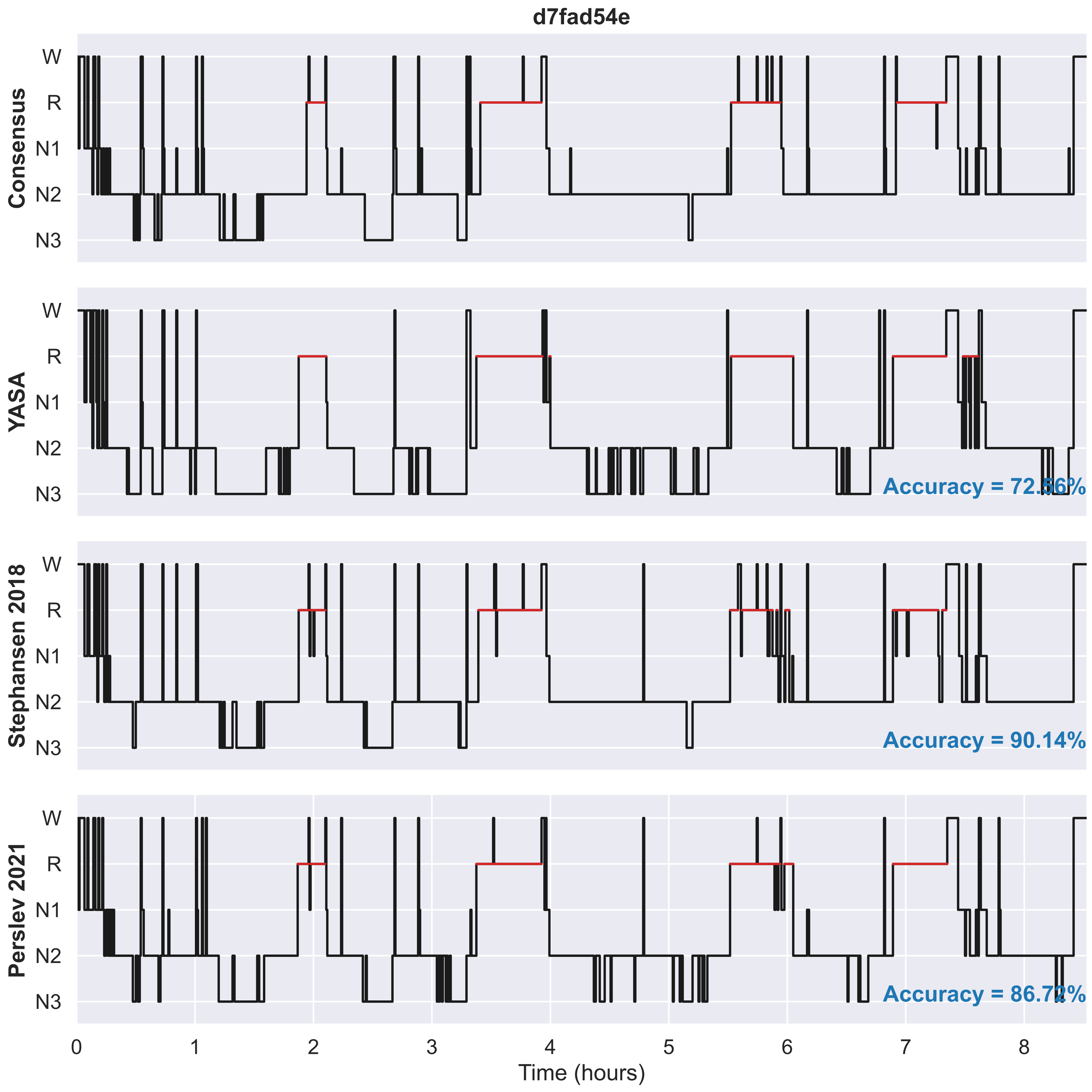
d7fad54e
Consensus
YASA
Stephansen 2018
Perslev 2021
W
R
N1
N2
N3
Accuracy = 72.56%
Accuracy = 90.14%
Accuracy = 86.72%
0
1
2
3
4
5
6
7
8
Time (hours)

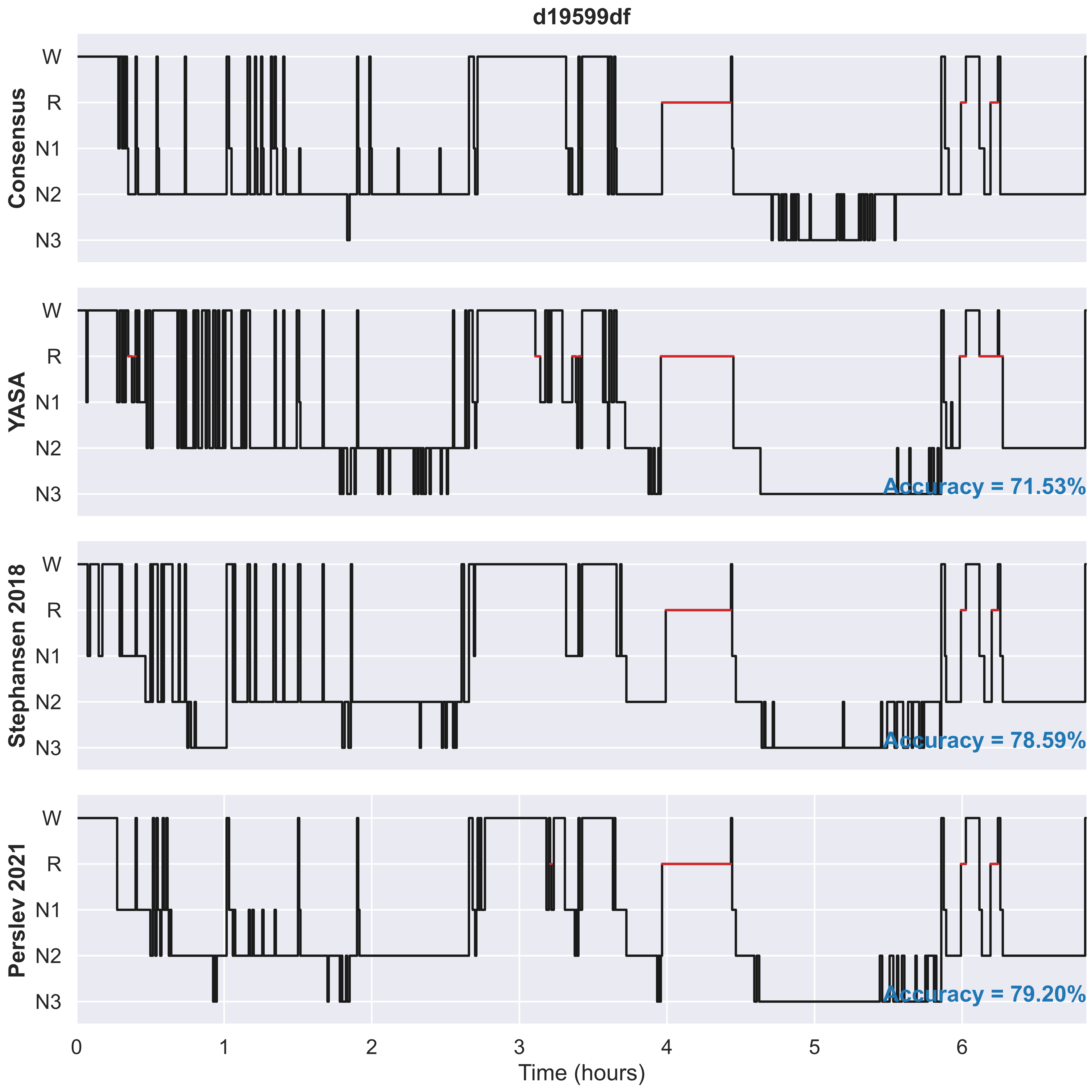
d19599df
Consensus
YASA
Accuracy = 71.53%
Stephansen 2018
Accuracy = 78.59%
Perslev 2021
Accuracy = 79.20%
W
R
N1
N2
N3
0
1
2
3
4
5
6
Time (hours)

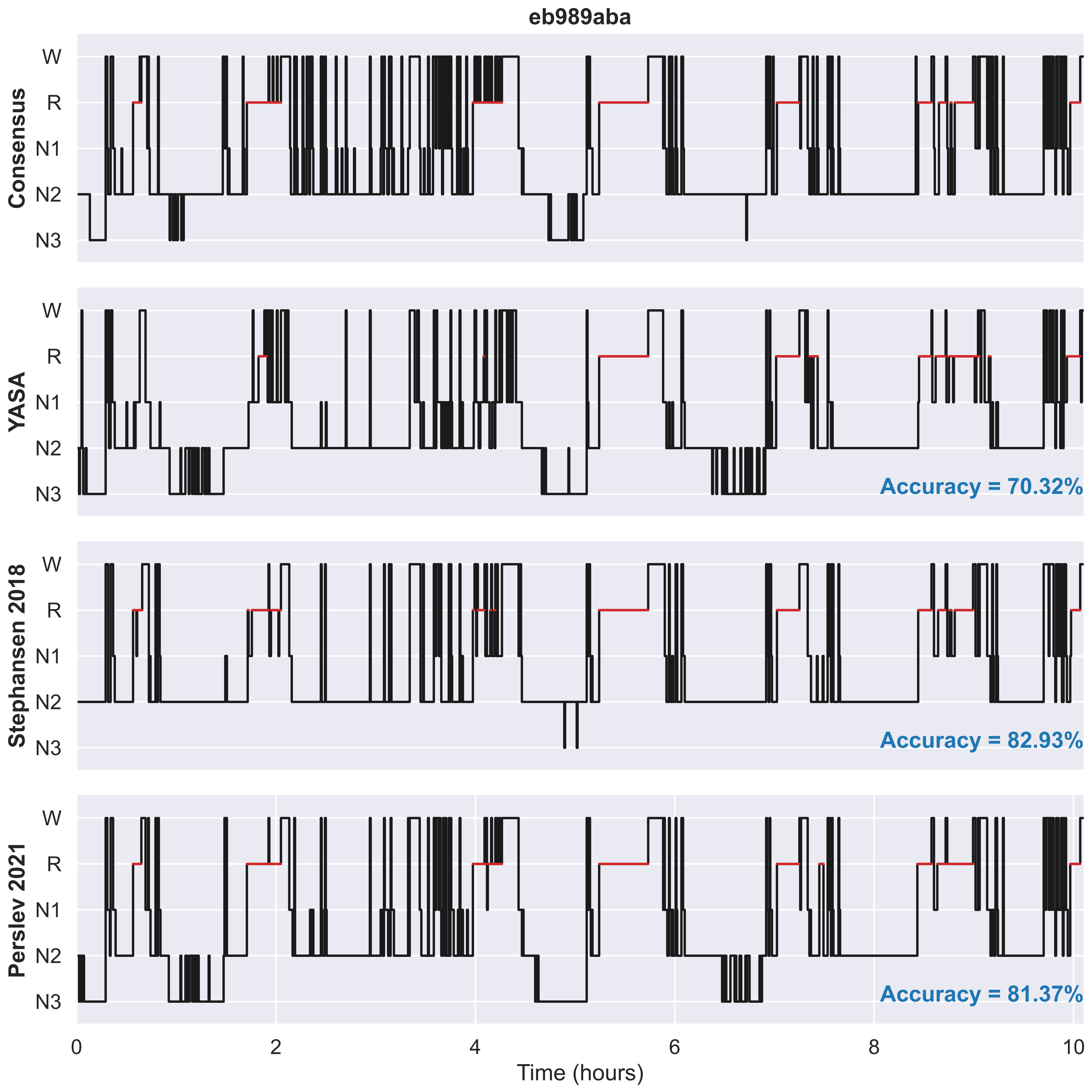
eb989aba
Consensus
YASA
Stephansen 2018
Perslev 2021
W
R
N1
N2
N3
Accuracy = 70.32%
Accuracy = 82.93%
Accuracy = 81.37%
0
2
4
6
8
10
Time (hours)

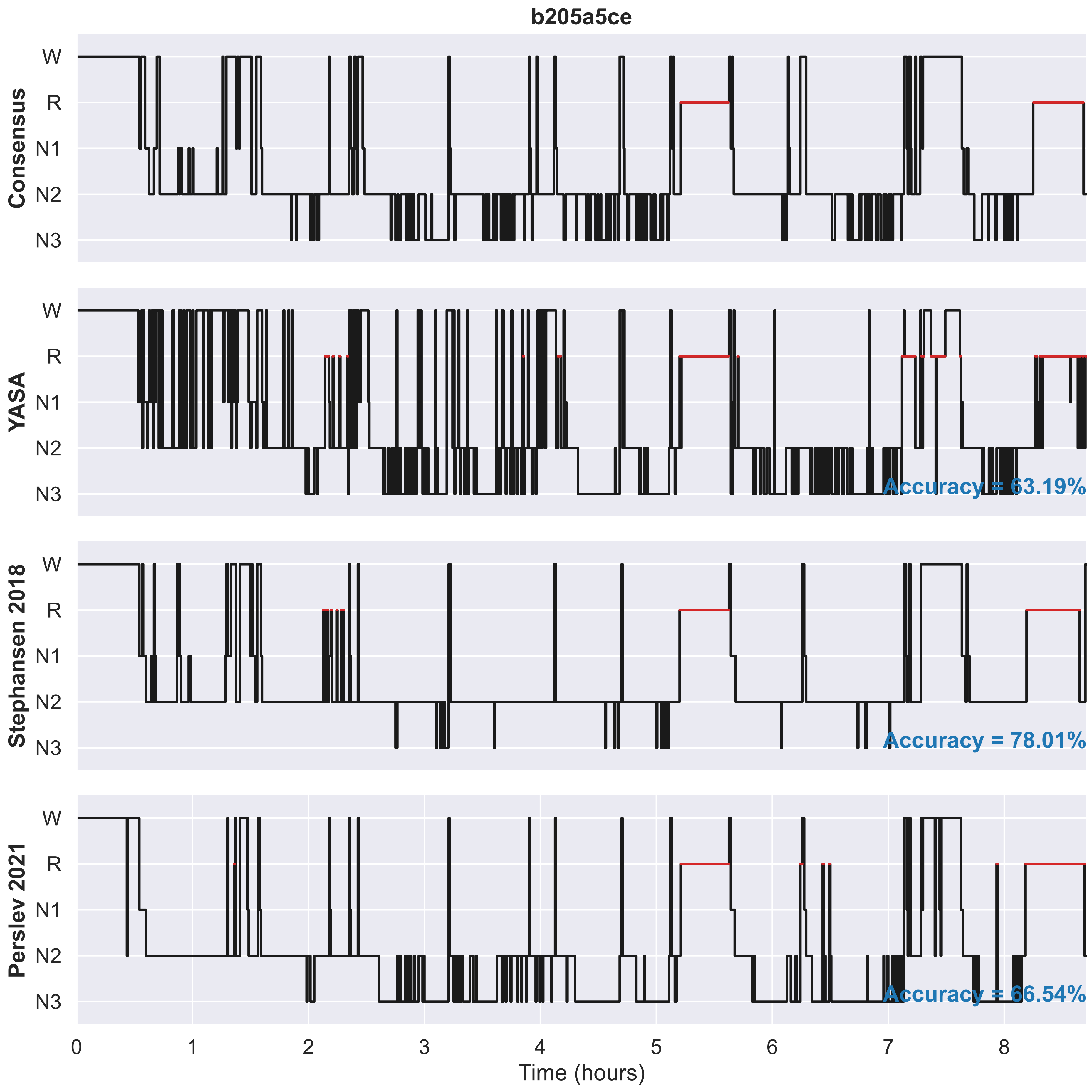

b205a5ce
Consensus
YASA
Stephansen 2018
Perslev 2021
W
R
N1
N2
N3
Accuracy = 63.19%
Accuracy = 78.01%
Accuracy = 66.54%
0
1
2
3
4
5
6
7
8
Time (hours)

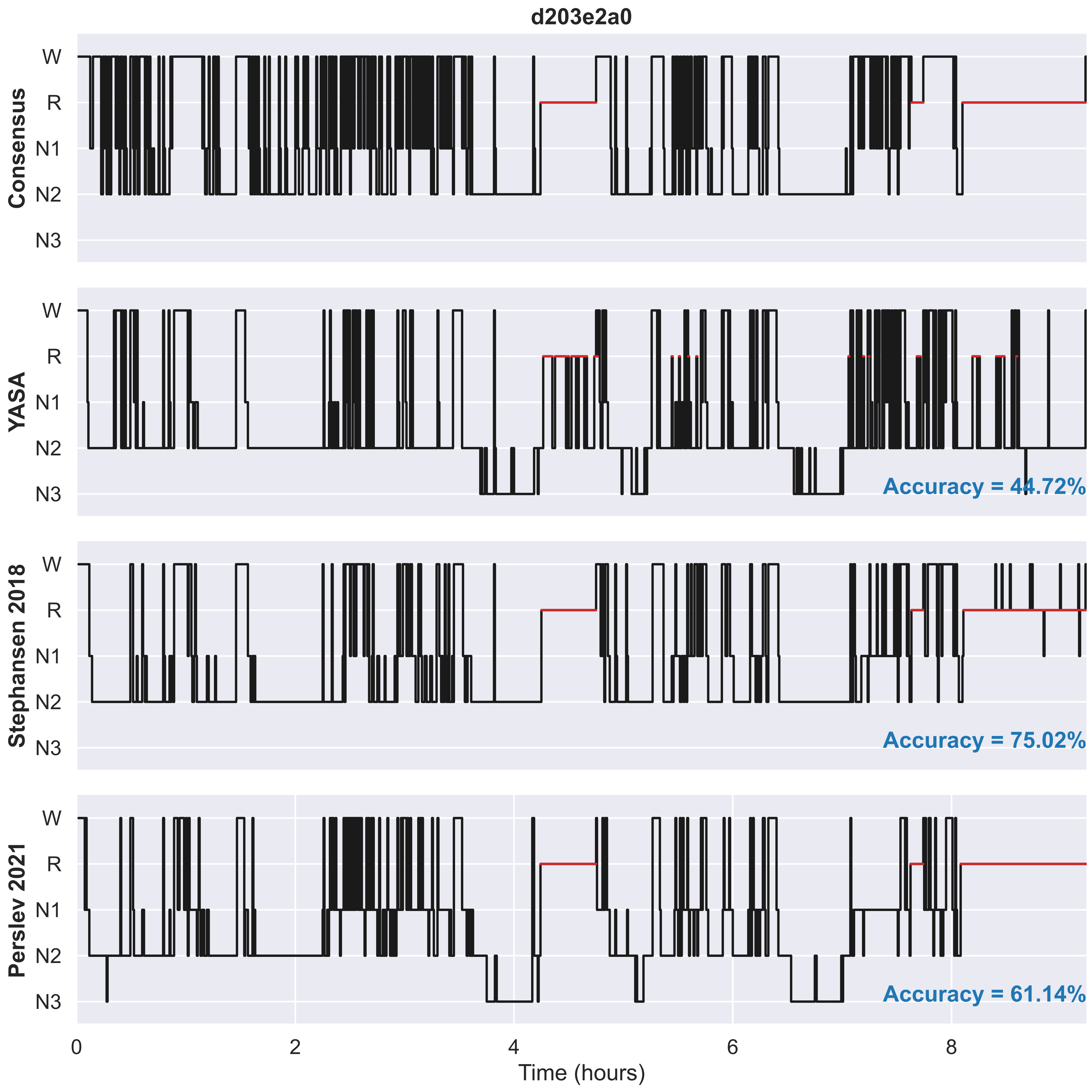

d203e2a0
Consensus
YASA
Stephansen 2018
Perslev 2021
W
R
N1
N2
N3
Accuracy = 44.72%
Accuracy = 75.02%
Accuracy = 61.14%
0
2
4
6
8
Time (hours)
