# Supplementary material for: An open-source, high-performance tool for automated sleep staging
Source: eLife. 2021 Oct 14;10:e70092. doi: 10.7554/eLife.70092 (PMC8516415; doi:10.7554/eLife.70092)
Supplement: Supplementary file 3. — A total of 49 combinations of past and centered rolling windows were tested, defined as the Cartesian product of the following time lengths for the past rolling average: [none, 1 min, 2 min, 3 min, 5 min, 7 min, 9 min] and the centered rolling weighted average: [none, 1.5 min, 2.5 min, 3.5 min, 5.5 min, 7.5 min, 9.5 min], where none indicates that no rolling window was applied. Cross-validation was performed using a threefold validation on the full training set, stratified by nights, such that a polysomnography (PSG) night was either present in the training and validation set, but never in both at the same time. For speed, only 50 trees were used in the classification algorithm. The ‘Mean’ column is the average of the accuracy and the five F1-scores. Note that the second best-ranked combination (9.5 min centered) has a slightly higher mean score; however, we chose to use a 7.5 min centered window (rank 1) in our final model because it had higher F1-scores for N2, N3, and rapid eye movement (REM) sleep. (B) Contributors of variability in accuracy. Relative importance (%) was estimated with a random forest on n = 585 nights from the testing set 1. The outcome variable of the model was the accuracy score of YASA against ground-truth sleep staging, calculated separately for each night. [file elife-70092-supp3.docx]

|  |  |  |  | F1 score | | | | |  |
| --- | --- | --- | --- | --- | --- | --- | --- | --- | --- |
| Rank | Past | Center | Accuracy | N1 | N2 | N3 | REM | Wake | Mean |
| 1 | 2 min | 7.5 min | 84.88 | 26.98 | 84.41 | 82.28 | 81.90 | 92.29 | 75.46 |
| 2 | 2 min | 9.5 min | 84.88 | 27.12 | 84.40 | 82.25 | 81.87 | 92.30 | 75.47 |
| 3 | 1 min | 7.5 min | 84.98 | 26.31 | 84.58 | 82.35 | 81.95 | 92.37 | 75.42 |
| 4 | 1 min | 9.5 min | 84.98 | 26.33 | 84.56 | 82.34 | 81.94 | 92.38 | 75.42 |
| 5 | 3 min | 1.5 min | 84.87 | 26.91 | 84.48 | 82.35 | 81.56 | 92.29 | 75.41 |
| 6 | 2 min | 5.5 min | 84.89 | 26.57 | 84.43 | 82.33 | 81.86 | 92.29 | 75.39 |
| 7 | 3 min | 2.5 min | 84.87 | 26.52 | 84.46 | 82.36 | 81.84 | 92.23 | 75.38 |
| 8 | 3 min | 3.5 min | 84.88 | 26.22 | 84.44 | 82.38 | 81.97 | 92.25 | 75.36 |
| 9 | 5 min | 2.5 min | 84.95 | 25.78 | 84.51 | 82.44 | 82.15 | 92.30 | 75.35 |
| 10 | 1 min | 5.5 min | 84.95 | 26.06 | 84.59 | 82.36 | 81.79 | 92.33 | 75.35 |
| 34 | 2 min | None | 84.45 | 26.33 | 84.11 | 82.28 | 79.99 | 92.09 | 74.88 |
| 39 | None | 5.5 min | 84.68 | 22.83 | 84.25 | 82.32 | 81.51 | 92.18 | 74.63 |
| 49 | None | None | 82.75 | 18.20 | 82.54 | 81.46 | 74.57 | 91.25 | 71.79 |

Supplementary File 3a: Cross-validation of the best time length for the temporal smoothing of the features.

A total of 49 combinations of past and centered rolling windows were tested, defined as the cartesian product of the following time lengths for the past rolling average: [None, 1 min, 2 min, 3 min, 5 min, 7 min, 9 min] and the centered rolling weighted average: [None, 1.5 min, 2.5 min, 3.5 min, 5.5 min, 7.5 min, 9.5 min], where None indicates that no rolling window was applied. Cross-validation was performed using a 3-fold validation on the full training set, stratified by nights, such that a PSG night was either present in the training and validation set, but never in both at the same time. For speed, only 50 trees were used in the classification algorithm. The “Mean” column is the average of the accuracy and the 5 F1-scores. Note that the second best-ranked combination (9.5 min centered) has a slightly higher mean score, however, we chose to use a 7.5 min centered window (rank 1) in our final model because it had higher F1-scores for N2, N3 and REM sleep.

| **Rank** | **Features** | **%Importance** |
| --- | --- | --- |
| 1 | % N1 sleep | 20.70 |
| 2 | % stage transitions | 20.19 |
| 3 | AHI | 8.32 |
| 4 | % N2 sleep | 8.16 |
| 5 | % N3 sleep | 7.92 |
| 6 | % wakefulness | 7.80 |
| 7 | % REM | 5.94 |
| 8 | Total duration | 5.77 |
| 9 | BMI | 4.95 |
| 10 | Age | 4.81 |
| 11 | Sex | 1.55 |
| 12 | Hypertension | 1.11 |
| 13 | Diabete | 0.91 |
| 14 | Race (caucasian) | 0.59 |
| 15 | Race (african) | 0.52 |
| 16 | Depression | 0.31 |
| 17 | Race (asian or pacific islander) | 0.24 |
| 18 | Race (hispanic) | 0.17 |
| 19 | Insomnia | 0.05 |

Supplementary File 3b: Contributors of variability in accuracy.

Relative importance (%) was estimated with a random forest on n=585 nights from the testing set 1. The outcome variable of the model was the accuracy score of YASA against ground-truth sleep staging, calculated separately for each night.
