# Supplementary material for: An open-source, high-performance tool for automated sleep staging
Source: eLife. 2021 Oct 14;10:e70092. doi: 10.7554/eLife.70092 (PMC8516415; doi:10.7554/eLife.70092)
Supplement: Supplementary file 4. — Importantly, the algorithm uses three different versions of all time-domain and frequency-domain features: (1) the raw feature, expressed in the original unit of data (e.g., µV for the standard deviation and interquartile range), (2) a smoothed and normalized version of that feature using a 7.5 min triangular-weighted rolling average, and (3) a smoothed and normalized version of that feature using a past 2 min rolling average. Normalization is done after smoothing on a per-night basis with a robust method based on the 5–95% percentiles. Frequency-domain features are based on a Welch’s periodogram with a 5 s window (= 0.2 Hz resolution). [file elife-70092-supp4.docx]

| **Features** | **Channel** | | |
| --- | --- | --- | --- |
|  | EEG | EOG | EMG |
| ***Time-domain features*** |  |  |  |
| Standard deviation | ✓ | ✓ | ✓ |
| Interquartile range | ✓ | ✓ | ✓ |
| Skewness | ✓ | ✓ | ✓ |
| Kurtosis | ✓ | ✓ | ✓ |
| Number of zero-crossings | ✓ | ✓ | ✓ |
| Hjorth mobility | ✓ | ✓ | ✓ |
| Hjorth complexity | ✓ | ✓ | ✓ |
| Higuchi fractal dimension | ✓ | ✓ | ✓ |
| Petrosian fractal dimension | ✓ | ✓ | ✓ |
| Permutation entropy | ✓ | ✓ | ✓ |
| ***Frequency-domain features*** |  |  |  |
| Absolute spectral power in the 0.4-30 Hz band | ✓ | ✓ | ✓ |
| Relative spectral power in the slow delta band (0.4-1 Hz) | ✓ | ✓ |  |
| Relative spectral power in the fast delta band (1-4 Hz) | ✓ | ✓ |  |
| Relative spectral power in the theta band (4-8 Hz) | ✓ | ✓ |  |
| Relative spectral power in the alpha band (8-12 Hz) | ✓ | ✓ |  |
| Relative spectral power in the sigma band (12-16 Hz) | ✓ | ✓ |  |
| Relative spectral power in the beta band (16-30 Hz) | ✓ | ✓ |  |
| Alpha / theta spectral power ratio | ✓ |  |  |
| Delta / beta spectral power ratio | ✓ |  |  |
| Delta / sigma spectral power ratio | ✓ |  |  |
| Delta / theta spectral power ratio | ✓ |  |  |
| ***Time features*** |  |  |  |
| Time, in hours from the beginning of the recording | N/A | | |
| Time, normalized from 0 (beginning of recording) to 1 (end) | N/A | | |
| ***Demographics features*** |  |  |  |
| Age | N/A | | |
| Sex | N/A | | |

Table S3: Features used in the final model.

All the features are calculated for each consecutive 30-sec epoch across the night, starting from the first sample of the polysomnography recording. Importantly, the algorithm uses 3 different versions of all time-domain and frequency-domain features: 1) the raw feature, expressed in the original unit of data (e.g. µV for the standard deviation and interquartile range), 2) a smoothed and normalized version of that feature using a 7.5 min triangular-weighted rolling average, and 3) a smoothed and normalized version of that feature using a past 2 min rolling average. Normalization is done after smoothing on a per-night basis with a robust method based on the 5-95% percentiles. Frequency-domain features are based on a Welch’s periodogram with a 5-sec window (= 0.2 Hz resolution).
