# Supplementary material for: An open-source, high-performance tool for automated sleep staging
Source: eLife. 2021 Oct 14;10:e70092. doi: 10.7554/eLife.70092 (PMC8516415; doi:10.7554/eLife.70092)
Supplement: Transparent reporting form [file elife-70092-transrepform1.docx]

***eLife’s* transparent reporting form**

We encourage authors to provide detailed information *within their submission* to facilitate the interpretation and replication of experiments. Authors can upload supporting documentation to indicate the use of appropriate reporting guidelines for health-related research (see [EQUATOR Network](http://www.equator-network.org/%20)), life science research (see the [BioSharing Information Resource](https://biosharing.org/)), or the [ARRIVE guidelines](http://www.plosbiology.org/article/info:doi/10.1371/journal.pbio.1000412) for reporting work involving animal research. Where applicable, authors should refer to any relevant reporting standards documents in this form.

If you have any questions, please consult our Journal Policies and/or contact us: [editorial@elifesciences.org](mailto:editorial@elifesciences.org).

**Sample-size estimation**

- You should state whether an appropriate sample size was computed when the study was being designed
- You should state the statistical method of sample size computation and any required assumptions
- If no explicit power analysis was used, you should describe how you decided what sample (replicate) size (number) to use

Please outline where this information can be found within the submission (e.g., sections or figure legends), or explain why this information doesn’t apply to your submission:

This study’s aim was to develop and validate a sleep staging algorithm on a large and representative database of polysomnography recordings. No explicit power analysis was used when this study was being designed, but the sample size of the current study is larger than typical for similar validation studies. More details about the final sample size of the training and testing sets can be found in the Descriptive statistics subsection of the Results section.

**Replicates**

- You should report how often each experiment was performed
- You should include a definition of biological versus technical replication
- The data obtained should be provided and sufficient information should be provided to indicate the number of independent biological and/or technical replicates
- If you encountered any outliers, you should describe how these were handled
- Criteria for exclusion/inclusion of data should be clearly stated
- High-throughput sequence data should be uploaded before submission, with a private link for reviewers provided (these are available from both GEO and ArrayExpress)

Please outline where this information can be found within the submission (e.g., sections or figure legends), or explain why this information doesn’t apply to your submission:

Validation of the algorithm was performed separately for each independent dataset. Criteria for inclusion and exclusion of polysomnography recordings are stated in the Methods section. The Python code to reproduce all the results and figures of this paper can be found at https://github.com/raphaelvallat/yasa_classifier.

**Statistical reporting**

- Statistical analysis methods should be described and justified
- Raw data should be presented in figures whenever informative to do so (typically when N per group is less than 10)
- For each experiment, you should identify the statistical tests used, exact values of N, definitions of center, methods of multiple test correction, and dispersion and precision measures (e.g., mean, median, SD, SEM, confidence intervals; and, for the major substantive results, a measure of effect size (e.g., Pearson's r, Cohen's d)
- Report exact p-values wherever possible alongside the summary statistics and 95% confidence intervals. These should be reported for all key questions and not only when the p-value is less than 0.05.

Please outline where this information can be found within the submission (e.g., sections or figure legends), or explain why this information doesn’t apply to your submission:

Statistical analysis methods are described within the Methods section, in the Performance evaluation subsection. Performance metrics and p-values (when applicable) are stated within the Results section and raw data is presented in the figures. The Python code to reproduce all the statistical results and figures of this paper can be found at https://github.com/raphaelvallat/yasa_classifier.

(For large datasets, or papers with a very large number of statistical tests, you may upload a single table file with tests, Ns, etc., with reference to sections in the manuscript.)

**Group allocation**

- Indicate how samples were allocated into experimental groups (in the case of clinical studies, please specify allocation to treatment method); if randomization was used, please also state if restricted randomization was applied
- Indicate if masking was used during group allocation, data collection and/or data analysis

Please outline where this information can be found within the submission (e.g., sections or figure legends), or explain why this information doesn’t apply to your submission:

Randomization and splitting of the polysomnography records into the training and testing sets is described within the Methods, in the Datasets subsection. The code used to generate the training and testing sets can be found at: https://github.com/raphaelvallat/yasa_classifier/blob/master/00_randomize_train_test.ipynb

**Additional data files (“source data”)**

- We encourage you to upload relevant additional data files, such as numerical data that are represented as a graph in a figure, or as a summary table
- Where provided, these should be in the most useful format, and they can be uploaded as “Source data” files linked to a main figure or table
- Include model definition files including the full list of parameters used
- Include code used for data analysis (e.g., R, MatLab)
- Avoid stating that data files are “available upon request”

Please indicate the figures or tables for which source data files have been provided:

All polysomnography data can be requested from the NSRR website (http://sleepdata.org). The Dreem Open Dataset can be found at https://github.com/Dreem-Organization/dreem-learning-open. The source code and documentation of the algorithm is available at https://github.com/raphaelvallat/yasa. The Python code to reproduce all the results and figures of this paper can be found at https://github.com/raphaelvallat/yasa_classifier. All analyses were conducted in Python 3.8 using scikit-learn 0.23.2 (Pedregosa et al., 2011) and lightgbm 3.0.0 (Ke et al., 2017).
